# Common Genetic Variation and the Control of HIV-1 in Humans

**DOI:** 10.1371/journal.pgen.1000791

**Published:** 2009-12-24

**Authors:** Jacques Fellay, Dongliang Ge, Kevin V. Shianna, Sara Colombo, Bruno Ledergerber, Elizabeth T. Cirulli, Thomas J. Urban, Kunlin Zhang, Curtis E. Gumbs, Jason P. Smith, Antonella Castagna, Alessandro Cozzi-Lepri, Andrea De Luca, Philippa Easterbrook, Huldrych F. Günthard, Simon Mallal, Cristina Mussini, Judith Dalmau, Javier Martinez-Picado, José M. Miro, Niels Obel, Steven M. Wolinsky, Jeremy J. Martinson, Roger Detels, Joseph B. Margolick, Lisa P. Jacobson, Patrick Descombes, Stylianos E. Antonarakis, Jacques S. Beckmann, Stephen J. O'Brien, Norman L. Letvin, Andrew J. McMichael, Barton F. Haynes, Mary Carrington, Sheng Feng, Amalio Telenti, David B. Goldstein, NIAID Center for HIV/AIDS Vaccine Immunology (CHAVI)

**Affiliations:** 1 Center for Human Genome Variation, Duke Institute for Genome Sciences and Policy, Duke University, Durham, North Carolina, United States of America; 2 Genomic Analysis Facility, Duke Institute for Genome Sciences and Policy, Duke University, Durham, North Carolina, United States of America; 3 Institute of Microbiology, University of Lausanne, Lausanne, Switzerland; 4 Division of Infectious Diseases and Hospital Epidemiology, University Hospital, University of Zürich, Zürich, Switzerland; 5 Behavioral Genetics Center, Institute of Psychology, Chinese Academy of Sciences, Beijing, China; 6 Clinic of Infectious Diseases, Vita-Salute San Raffaele University and Diagnostica and Ricerca San Raffaele, Milan, Italy; 7 Research Department of Infection and Population Health, University College London, London, United Kingdom; 8 Institute of Clinical Infectious Diseases, Catholic University of the Sacred Heart, Rome, Italy; 9 Academic Department of HIV/GUM, Kings College London at Guy's, King's, and St. Thomas' Hospitals, Weston Education Centre, London, United Kingdom; 10 Centre for Clinical Immunology and Biomedical Statistics, Institute for Immunology and Infectious Diseases, Royal Perth Hospital and Murdoch University, Perth, Australia; 11 Infectious Diseases Clinics, Azienda Ospedaliero-Universitaria, Modena, Italy; 12 IrsiCaixa Foundation and Hospital Germans Trias i Pujol, Badalona, Spain; 13 Institució Catalana de Recerca i Estudis Avançats (ICREA), Barcelona, Spain; 14 Hospital Clinic – IDIBAPS, University of Barcelona, Barcelona, Spain; 15 Department of Infectious Diseases, Copenhagen University Hospital, Rigshospitalet, Copenhagen, Denmark; 16 Division of Infectious Diseases, Northwestern University Feinberg School of Medicine, Chicago, Illinois, United States of America; 17 Infectious Diseases and Microbiology, Graduate School of Public Health, University of Pittsburgh, Pittsburgh, Pennsylvania, United States of America; 18 Department of Medicine, David Geffen School of Medicine, University of California Los Angeles, Los Angeles, California, United States of America; 19 Department of Molecular Microbiology and Immunology, Johns Hopkins Bloomberg School of Public Health, Baltimore, Maryland, United States of America; 20 Department of Epidemiology, Johns Hopkins Bloomberg School of Public Health, Baltimore, Maryland, United States of America; 21 Genomics Platform, National Centre of Competence in Research “Frontiers in Genetics,” University of Geneva, Geneva, Switzerland; 22 Department of Genetic Medicine and Development, University of Geneva Medical School, Geneva, Switzerland; 23 Department of Medical Genetics, University of Lausanne, and Service of Medical Genetics, Centre Hospitalier Universitaire Vaudois, Lausanne, Switzerland; 24 Laboratory of Genomic Diversity, National Cancer Institute, Frederick, Maryland, United States of America; 25 Division of Viral Pathogenesis, Beth Israel Deaconess Medical Center, Harvard Medical School, Boston, Massachusetts, United States of America; 26 MRC Human Immunology Unit, Weatherall Institute of Molecular Medicine, John Radcliffe Hospital, Oxford, United Kingdom; 27 Duke Human Vaccine Institute, Duke University, Durham, North Carolina, United States of America; 28 Cancer and Inflammation Program, Laboratory of Experimental Immunology, SAIC-Frederick, Inc., National Cancer Institute at Frederick, Frederick, Maryland, United States of America; 29 Ragon Institute of Massachusetts General Hospital, MIT and Harvard, Boston, Massachusetts, United States of America; University of Oxford, United Kingdom

## Abstract

To extend the understanding of host genetic determinants of HIV-1 control, we performed a genome-wide association study in a cohort of 2,554 infected Caucasian subjects. The study was powered to detect common genetic variants explaining down to 1.3% of the variability in viral load at set point. We provide overwhelming confirmation of three associations previously reported in a genome-wide study and show further independent effects of both common and rare variants in the Major Histocompatibility Complex region (MHC). We also examined the polymorphisms reported in previous candidate gene studies and fail to support a role for any variant outside of the MHC or the chemokine receptor cluster on chromosome 3. In addition, we evaluated functional variants, copy-number polymorphisms, epistatic interactions, and biological pathways. This study thus represents a comprehensive assessment of common human genetic variation in HIV-1 control in Caucasians.

## Author Summary

The ability to spontaneously control HIV-1 upon infection is highly variable between individuals. To evaluate the contribution of variation in human genes to differences in plasma viral load and in disease progression rates, we performed a genome-wide association study in >2,500 HIV–infected individuals. This study achieved two goals: it completed the analysis of common variation influencing viral control, and it re-assessed the majority of previously reported genetic associations. We show that genetic variants located near the *HLA-B* and *HLA-C* genes are the strongest determinants of viral control, and that other independent associations exist in the same region of chromosome 6, the Major Histocompatibility Complex, known to contain a large number of genes involved in immune defense. We could not replicate most of the previously published associations with HIV candidate genes in this large, well-characterized cohort. Overall, common human genetic variation, together with demographic variables, explains up to 22% of the variability in viral load in the Caucasian population.

## Introduction

The clinical outcome of HIV-1 infection is highly variable and determined by complex interactions between virus, host and environment. Several human genetic factors have been reported to modulate HIV-1 disease [1],[2], but current knowledge only explains a small fraction of the observed variability in the course of infection. Our first genome-wide association study (GWAS) of human genetic variants that associate with HIV-1 control analyzed 486 individuals of European ancestry and identified two genome-wide significant determinants of viremia at set point, and one determinant of disease progression [3]. The 3 single nucleotide polymorphisms (SNPs) collectively explained 14% of the variation in viral load at set point and 10% of the variation in disease progression. All 3 were located in the Major Histocompatibility Complex (MHC) region on chromosome 6, confirming, in a genome-wide context, the essential role played by the MHC region in HIV-1 control [4],[5],[6]. Since then, the findings have been replicated by several independent groups that used either targeted genotyping [7],[8],[9] or whole genome approaches [10],[11].

The power of our initial study was limited to the detection of common variants (with minor allele frequency of 5% or more) which explain a sizable fraction of the phenotypic variability: it had an 80% power of identifying SNPs that explain at least 5% of the variability. Here we have increased the sample size to 2554 which allows us to provide a much more thorough investigation of the role of common variation in the control of HIV-1: considering the final number of participants included in the analysis, the study was powered to detect the effects of common variants down to 1.3% of explained variability.

One surprising feature of the first genome-wide investigation was that it failed to identify any of the many non-MHC candidate gene variants that have been reported to associate with HIV-1 disease outcomes over the past 15 years. Candidate gene studies claimed associations for variants in genes selected for their known or suspected role in HIV-1 pathogenesis and in immune response [1],[2] [compiled in http://www.hiv-pharmacogenomics.org]. Collectively, this body of work has implicated various components of innate, adaptive, and intrinsic immunity in HIV-1 control, cellular co-factors important in viral life cycle, and quite unexpected candidates such as the vitamin D receptor. We here use our large sample size and the power of genome-wide data, which allows precise correction for population stratification, to evaluate most candidate gene discoveries.

Genome-wide analysis also makes it possible to assess genetic interactions, copy number polymorphisms, enrichment of gene sets and of functional variants: these analyses were pursued in the present work. Collectively, our study circumscribes the impact of common variation in the control of HIV-1 in an adult and predominantly male Caucasian population.

## Results

### Subjects

A total of 2554 HIV-1 infected individuals of self-reported Caucasian ancestry were genotyped on Illumina whole-genome chips (Table S1). Participants were recruited between 1984 and 2007 in one of the 9 cohorts forming the Euro-CHAVI Consortium (N = 1397, including 486 subjects that were included in our previous study [3], 75.7% male, median age: 33 years) or in the MACS cohort (N = 1157, 100% male, median age: 33 years). A subset of 1113 patients had a proven date of seroconversion (“seroconverters”) and the remainder had a confirmed stable viremia profile but no known date of infection (“seroprevalent” patients). Several quality control steps (*Text S1*) resulted in the exclusion of 115 individuals with insufficient genotype call rates, of 17 individuals that were found to be genetically related with another study subject, and of 5 individuals with a gender discrepancy between phenotype and genotype data. To control for population stratification, we performed a principal component analysis of the whole-genome genotyping data (Eigenstrat [12], *Text S1*), which identified 12 significant axes after exclusion of 55 outlier subjects. The most significant principal component is a north-to-south European axis that has already been described [13] (Figure S1). A total of 2362 individuals were included in the set point association analyses (including 486 subjects studied before [3]), and 1071 seroconverters were eligible for the analysis of disease progression (including 337 subjects studied before [3]). A subset of 1204 subjects had complete 4-digit HLA Class I results and could be included in models assessing the respective influences of SNPs and HLA alleles on HIV-1 control.

### Common variants and variation in viral load at set point

All QC-passed SNPs (Text S1, Table S11) were tested for association with HIV-1 viremia at set point in separate linear regression models that included gender, age, and the 12 significant PC axes as covariates. The global distribution of resulting p-values was very close to the null expectation (λ = 1.006, Figure S2) indicating that stratification was adequately controlled [14]. Male gender and older age both associated with higher set point (p = 1.9E-21 and p = 2.6E-05, respectively) and explained 4% of the inter-individual variability. The population sub-structure, reflected in the 12 significant PC axes, explained an additional 3.4%.

**Figure 1:**
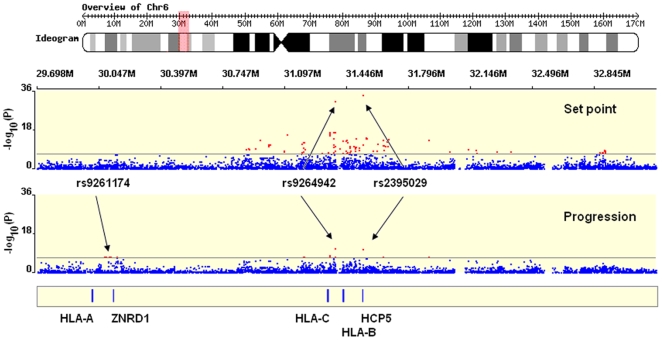
Significant hits in the MHC region. Representation of a 3 Mb stretch in the MHC region, encompassing the HLA Class I gene loci and the genome-wide significant SNPs identified in the study (red dots represents SNPs with p-value<5E–08). Results are shown for set point (upper plot) and for progression (lower plot). The figure was created with WGAViewer [40].

**Figure 2:**
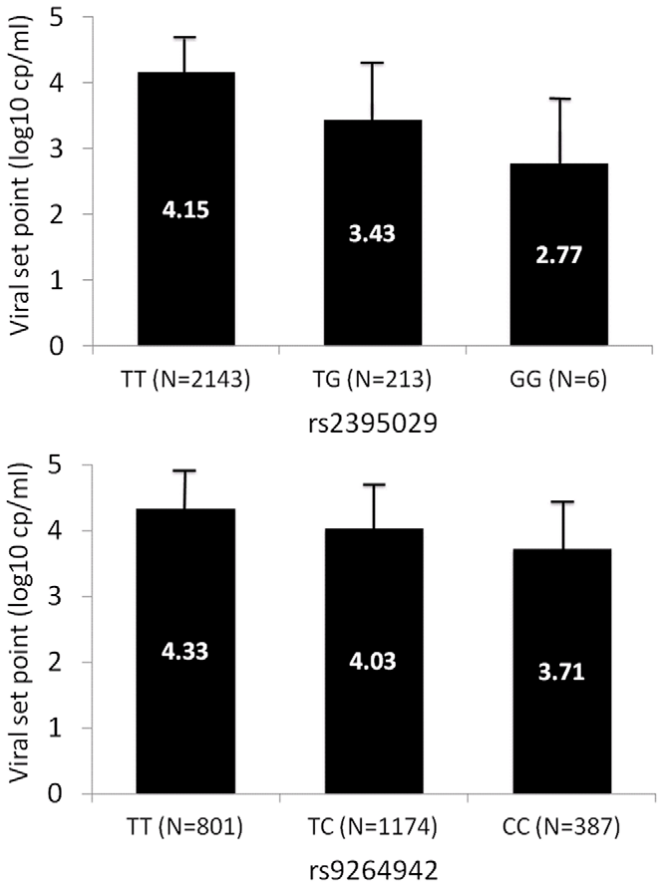
Correlation between HIV-1 set point and the genotypes of the top associated SNPs. HIV-1 viremia at set point strongly associates with rs2395029 (upper panel) and rs9264942 (lower panel) genotypes. The rs2395029 minor allele G has a frequency of 4.8% and each copy of this allele associates with a 0.7 log lower set point. The rs9264942 minor allele C has a frequency of 41.2%, and each copy of this allele associates with a 0.3 log lower set point. Mean and Standard Deviation (error bars) are represented for the respective genotypes.

The 2 SNPs previously reported as genome-wide significant [3] were confirmed to be the strongest determinants of variation in HIV-1 viral load (Figure 1 and Figure 2): rs2395029, an *HCP5* T>G variant, which is known to be an almost perfect proxy for HLA-B*5701 in Caucasians [15],[16] (p = 4.5E–35), and rs9264942, a T>C SNP located in the 5′ region of *HLA-C*, 35 kb away from transcription initiation (p = 5.9E–32). Of note, the association signals were also convincingly genome-wide significant in an analysis restricted to the subjects that were not included in our initial study (Table 1). Each SNP explained 5 to 6% of the variability in set point viremia, as determined by the increase in the r2 value of the respective linear regression models: the effect size estimates are smaller in the expanded data set than in the previously reported study. This is due, at least in part, to the inclusion of seroprevalent subjects with less stringent phenotype definition. Interestingly, we observed a break in the strong linkage disequilibrium (LD) between *HCP5* and *HLA-B* in 9/1204 subjects with HLA Class I results (0.7%, r2 = 0.93): the set point values were lower for the 4 patients that had B*5701 without the rs2395029 minor allele than for the 5 patients with a G at rs2395029 but without B*5701 (median [IQR] log10 HIV-1 cp/ml  =  3.14 [2.77–3.68] vs. 4.22 [4.14–4.28] respectively (Table S2); p = 0.05, Kruskal-Wallis rank test). Consequently, the association with set point was stronger for B*5701 (p = 3.8E–19) than it was for rs2395029 (p = 1.7E–17) in the same subset of patients.

**Table 1:** P-values and population effect sizes (or explained fraction of the inter-individual variability) for the strongest determinants of HIV-1 viremia at set point.

SNP	MAF	Gene	P-value (initial, N = 486)	Effect Size (initial)	P-value (replication, N = 1896)	Effect Size (replication)	P-value (all, N = 2362)	Effect Size (all)
rs2395029	4.8%	HCP5/B*5701	9.4E–12	9.6%	4.2E–23	4.9%	4.5E–35	5.8%
rs9264942	41.2%	HLA-C	3.8E–09	6.5%	1.6E–23	4.8%	5.9E–32	5.3%

Results are shown for the subjects that were included in our initial study [3] (initial), for the subset of subjects that are new to the present study (replication), and for the global study population (all). MAF: Minor allele frequency.

A major challenge in assessing association evidence in the MHC is the long range pattern of LD, complicating the definitive identification of causal variants and making it necessary to consider evidence for association in the region as a whole. For this reason, we evaluated the evidence for independent effects of the two reported associations. Since the *HCP5* and the *HLA-C* variants are in partial LD (r2 = 0.06, D′ = 0.86), the combined strength of their associations with set point is less than the sum of the signals measured separately. It is also interesting to note that in 95% of cases the rs2395029 minor allele, tagging B*5701, is found in combination with the controlling C allele at *HLA-C* rs9264942. This means that the protective effect in this haplotype is a combination of the effect of both alleles and that analyses that are not adjusted for the *HLA-C* variant overestimate the B*5701-related effect. Nonetheless, nested regression models clearly demonstrated that each of the variants is independently genome-wide significant (p = 1.8E–23 for rs2395029; p = 2.4E–20 for rs9264942). We therefore can conclude unequivocally that the two SNPs represent independent effects: they together explain 9% of the variability in HIV-1 set point.

### Further independent associations in the MHC region

We next addressed the question of whether there are any additional, independent and significant SNP effects in the MHC region. In addition to the top 2 associated variants, 86 other SNPs met the criteria of genome-wide significance (p<5E–08), all located in the MHC (Figure 1, Table S9).

We included a total of 331 MHC SNPs with p<1E–04 in a conservative forward selection algorithm within a linear regression model. SNPs were tested and selected into the model one at a time, independently of the top 2 associated variants and of the other covariates (gender, age and Eigenstrat axes). In order to control for multiple testing, a permutation procedure was performed to assess the empirical significance cut-off value. Four additional SNPs were found to significantly associate with set point in models including the previously associated variants (Table S3): [1] rs259919, located in an intron of the uncharacterized *C6orf12* gene, 3.5 kb away from the *ZNRD1* gene in the 5′ region; [2] rs9468692, located in the 3′ region of the *TRIM10* gene, in high LD (r2 = 0.87) with a non-synonymous coding SNP in the first exon of *TRIM10* - rs12212092: 279A>G; H65R, which is predicted to be a high-risk change by FastSNP [17] (non-conservative amino acid change; possibly splicing regulation); [3] rs9266409, located in the 3′ region of *HLA-B*, 12 kb away; [4] rs8192591, a non-synonymous coding SNP located in the 9th exon of the *NOTCH4* gene: 1739A>G; S534G (conservative amino acid change; possibly influencing splicing). We emphasize that these analyses demonstrate that there are further independent effects in the MHC region, but they do not prove that the specific SNPs implicated are responsible for those effects. Some or all of these SNPs are most likely markers for one or more variants that have not been genotyped and which provide aspects of viral control independent of the two previously reported associations.

As one possible contribution to these associations we assessed the HLA Class I alleles in the subset of 1204 subjects who had full MHC typing results. The 4-digit alleles were tested separately and in models including the identified MHC SNPs (Table 2, Table S4). 15 alleles were found to associate with set point, but only 4 of them (A*3201, B*1302, B*2705 and B*3502) had an independent effect that was still detectable in models including the top associated SNPs. Several *HLA-C* alleles associated significantly with viral control before but not after adjustment for the top SNPs (Table 2, Table S6). This is partly because all *HLA-C* alleles are in LD with the *HLA-C* -35 rs9264942. They can in fact be perfectly divided into 2 mutually exclusive groups on the basis of their LD with the rs9264942 C or T allele. The C-related alleles, as a group, strongly associated with lower setpoint (p = 2.8E–14) but failed to entirely recapitulate the rs9264942 association signal (p = 8.4E–16 in this group). Homozygosity for the HLA Class I loci also showed a weak independent association (p = 0.03 after adjustment for the SNPs). Altogether, a model including 6 SNPs, 4 alleles and homozygosity status shows that MHC variation explains 12% of the set point variability in this cohort.

**Table 2:** Associations between 4-digit HLA Class I alleles and HIV-1 set point in a subset of 1,204 subjects with full results.

HLA Allele	Model with HLA allele only	Model with HLA allele, rs2395029	Model with HLA allele, rs2395029, rs9264942	Model with HLA allele, rs2395029, rs9264942, rs259919, rs9468692, rs9266409, rs8192591	Effect
A*2402	3.4E–02	6.9E–02	1.1E–01	9.9E–01	
A*2501	2.8E–02	2.7E–02	1.2E–01	3.2E–01	
A*3201	5.0E–03	1.0E–03	6.0E–03	2.9E–02	protective
B*0702	7.0E–03	1.5E–02	4.3E–01	3.8E–01	
B*0801	7.0E–03	3.3E–02	5.8E–01	2.4E–01	
B*1302	2.0E–03	2.1E–04	1.2E–02	1.6E–02	protective
B*2705	5.2E–05	4.9E–06	2.0E–03	3.0E–03	protective
B*3502	1.9E–05	3.9E–05	6.2E–04	2.0E–03	deleterious
B*5601	4.2E–02	7.8E–02	1.9E–01	1.2E–01	
C*0202	1.0E–03	2.5E–04	6.0E–02	5.0E–02	
C*0401	5.0E–03	3.0E–02	9.3E–01	8.5E–01	
C*0602	1.2E–11	4.9E–02	7.1E–01	7.8E–01	
C*0701	5.4E–04	3.0E–03	1.8E–01	7.8E–01	
C*0702	3.4E–02	6.3E–02	9.9E–01	9.5E–01	
C*0802	1.2E–02	2.0E–03	1.6E–01	2.5E–01	

P-values are shown for all Class I alleles that have a nominally significant association with HIV-1 viral load at set point (with the exception of B*5701, discussed in the text). All linear regression models include gender, age and the 12 Eigenstrat axes as covariates. Most of the association signals disappear once the top associated SNPs are added. However, A*3201, B*1302, B*2705 and B*3502 still have an independent effect. See Table S4 for a complete list of all HLA Class I allele results. In addition, Table S5 lists all pairs of *HLA-B* and *HLA-C* alleles that are in LD (with an r2>0.1) and therefore can represent the same association signal (as for example in the case of HLA-C*0602, which is often on the same haplotype as HLA-B*5701).

### HIV-1 disease progression

We defined HIV-1 disease progression as the drop of CD4 T cell count to below 350 cells/ul or the initiation of potent antiretroviral treatment (cART) following a CD4 T cell count <500 cells/ul. A total of 1071 individuals with known date of seroconversion and at least two CD4 T cell determinations in absence of cART were included in a survival analysis. Of those, 765 (71.4%) progressed during follow-up: 612 (80%) because of a CD4 T cell value <350/ul and 153 (20%) because they started treatment with <500 CD4 T cells per ul.

**Figure 3:**
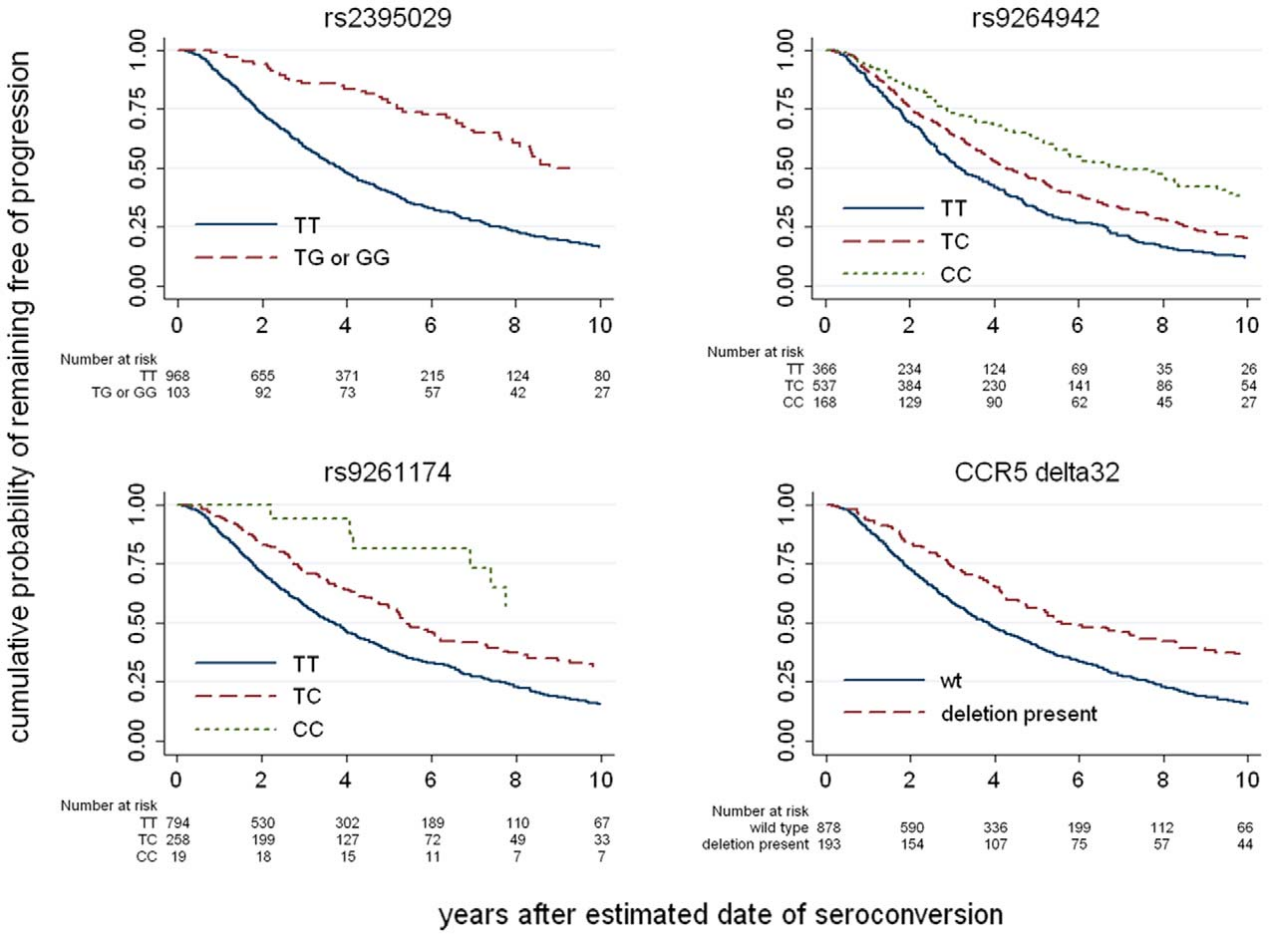
Kaplan-Meier survival estimates for the top associated variants. Results are shown for the 3 most associated SNPs indentified in the genome-wide progression scan and for *CCR5*-Δ32. The survival curves show, for each genotype, the proportion of the individuals that do not reach a progression outcome over the first 10 years after seroconversion.

The top associated variants were HCP5/B*5701 rs2395029 (p = 1.2E–11) and *HLA-C* rs9264942 (p = 6.4E–12) (Figure 1 and Figure 3, Table S10). If viral load at set point is added to the models, the association signals are much weaker (p = 0.001 for rs2395029 and p = 0.02 for rs9264942), demonstrating that the HCP5/B*5701 and *HLA-C* effects on disease progression are mainly driven by their impact on early viral control. Another set of variants reached genome-wide significance: rs9261174, rs3869068, rs2074480, rs7758512, rs9261129, rs2301753 and rs2074479 (p = 1.8E–08), in high-LD and located around the *ZNDR1* and *RNF39* genes, close to the *HLA-A* locus in the MHC region. This association is largely independent of viremia (p = 4.7E–05 in a model including set point as covariate), suggesting that a different mechanism of action is here modulating HIV disease progression. The causal variant(s) responsible for this association remains largely undetermined, and it seems possible that the association depends on the contribution of multiple causal sites. We do note, however, that no single HLA Class I allele can account for the association. Specifically, the recent claim that it is due entirely to the A10 serogroup of HLA-A alleles [7] is not supported by the LD data. When this claim is evaluated by including the A10 alleles in a regression model and testing the significance of the increased variation explained by the *ZNRD1* SNPs, we observe an independent additional effect of the SNPs (p = 0.03). Indeed, the identified SNPs still associate with progression in individuals without HLA-A10 (Figure S3). Conversely, HLA-A10 alleles do not significantly associate with progression in models that include the *ZNRD1* SNPs. The fact that A10 alleles (notably A*2501 and A*2601) are in LD with the *ZNRD1* SNPs (r2 = 0.46) is not sufficient evidence to assign responsibility for the association, although A10 may contribute to the association signal.

### Genetic variants in HIV candidate genes

We tested a total of 34 SNPs in 21 genes, representing 27 previously reported associations with HIV-1 control (individual SNPs or haplotypes) (Table 3) [1],[2],[18]. Nine SNPs were directly genotyped and 12 had a good proxy (r2>0.8) on all the chips that we used. Two were present on the Human1M chips only and 9 were not represented: these 11 SNPs were genotyped by TaqMan assays. The *CCL3L1* copy number polymorphism was also assessed and the absence of any association with HIV-1 control has been recently reported [19].

**Table 3:** Variants in HIV-1 candidate genes, previously reported to associate with viral control or disease progression.

group	SNP	gene	variant	genotyping	proxy	r2	model	p setpoint	*N*	p progression	*N*	effect
Chemokine receptors	rs333	CCR5	delta 32	TaqMan	no		dominant	1.7E–10	*2333*	3.5E–07	*1054*	*protective*
	rs1799988	CCR5	P1 - 627T>C	TaqMan	no		recessive	7.5E–05	*1791*	3.8E–04	*1012*	*deleterious*
	rs1799864	CCR2	V64I	TaqMan	no		dominant	8.1E–03	*2317*	1.5E–01	*1056*	*protective*
	rs3732378	CX3CR1	T280M	present on all chips	-		recessive	1.5E–01	*2362*	6.0E–01	1071	
Chemokines	rs1719134	CCL3/MIP1a	intron 459C>T	proxy on all chips	rs1634508	1	dominant	6.0E–01	*2362*	8.2E–01	*1071*	
	rs2107538	CCL5/RANTES	promoter -403G>A	proxy on all chips	rs2291299	1	haplotypes R1-R5: additive	2.9E–01	*2362*	2.3E–01	*1069*	
	rs2280788		promoter -28C>G	proxy on all chips	rs4251739	1						
	rs2280789		In1.1 T>C	proxy on all chips	rs2306630	1						
	rs1801157	SDF-1/CXCL12	SDF-1 3′A	proxy on all chips	rs10900029	1	recessive	8.6E–01	*2362*	9.2E–01	*1071*	
Cytokines	rs2243250	IL-4	promoter -589C>T	proxy on all chips	rs2243290	1	additive	3.4E–01	*2362*	6.3E–01	*1065*	
	rs1800872	IL-10	promoter -592C>A	proxy on all chips	rs3024490	1	dominant	6.5E–02	*2362*	1.4E–01	*1065*	
	rs1799946	DEFB1	promoter -52G>A	proxy on all chips	rs2741127	0.9	recessive	1.9E–01	*1830*	2.5E–01	*931*	
Intracellular life cycle	rs8177826	PPIA	promoter 1604C>G	TaqMan	no		dominant	6.0E–01	*1808*	6.3E–01	*1065*	
	rs6850		promoter 1650A>G	TaqMan	no		dominant	7.7E–01	*1753*	1.4E–01	*1065*	
	rs2292179	TSG101	promoter -183T>C	proxy on all chips	rs3781640	1	haplotypes	7.9E–01	*1792*	1.9E–01	*929*	
	rs1395319		intron 181A>C	TaqMan	no							
Intrisic immunity	rs8177832	APOBEC3G	NS coding H186R	present on all chips	-		additive	3.2E–01	*2362*	5.2E–01	*1071*	
	rs3740996	TRIM5a	NS coding H43Y	present on all chips	-		recessive	7.3E–01	*2362*	9.3E–01	*1071*	
	rs10838525		NS coding R136Q	present on all chips	-		additive	9.0E–01	*2362*	8.3E–01	*1071*	
Innate immunity	rs2287886	DC-SIGN/CD209	promoter -139T>C	present on all chips	-		additive	9.2E–01	*2362*	1.2E–01	*1065*	
	rs5030737	MBL2	NS coding R52C	1M chip + TaqMan	no		recessive	4.5E–01	*1735*	2.9E–01	*503*	
	rs1800450		NS coding G54D	present on all chips	-		recessive	4.3E–01	*2361*	9.0E–01	*1064*	
	rs1800451		NS coding G57E	1M chip + TaqMan	no		recessive	5.5E–01	*1728*	8.5E–01	*501*	
	rs352139	TLR9	intron 1174G>A	proxy on all chips	rs352163	0.9	additive	6.5E–01	*2362*	7.9E–01	*1065*	
	rs352140		syn coding P545P	proxy on all chips		0.9						
	rs3764880	TLR8	NS coding V1M	present on all chips	-		additive	5.0E–01	*2361*	9.3E–01	*1064*	
Others	rs601338	FUT2	W154stop	proxy on all chips	rs504963	0.8	dominant	1.1E–01	*2362*	7.0E–02	*1065*	
	rs1801274	FCGR2A	NS coding H131R	present on all chips	-		recessive	7.9E–01	*2360*	2.0E–01	*1065*	
	rs1544410	VDR	intron 8 variant	present on all chips	-		recessive	5.1E–01	*2360*	6.0E–01	*1064*	

See http://www.hiv-pharmacogenomics.org/pdf/ref_tbl_nat_history/The_complete_reference_table_for_HIV_natural_history_modifiers.pdf for references. Dominant or additive genetic models were used in the analyses for individual SNPs on the basis of their described effect and/or their minor allele frequencies. The P1 variant in the *CCR5* promoter region is defined by the SNP rs1799988 (627 C>T). Haplotypes R1 to R5 in the *CCL5* (*RANTES*) gene were defined using 2 promoter variants (−403C>G, defining haplotype R1, and −28C>G, defining haplotype R5), and 1 intronic variant (375T>C, or In1.1, present in haplotypes R3, R4 and R5). The haplotype R4 is defined by a −222T>C SNP that is monomorphic in Caucasians and was therefore absent in our study population. A combined variable was then defined and tested in additive models: 0 = putatively deleterious haplotypes (presence of an R3 haplotype in the absence of R1 and R5), 1 = neutral haplotypes (all other) and 2 =  haplotypes putatively protective (presence of an R1 or R5 haplotype in the absence of R3). For the *TSG101* gene, 2 SNPs defined haplotype B (−183T/181C), haplotype C (−183C/181C) and haplotype A (−183T/181A). Again, a combined variable was defined and tested in additive models: 0 = haplotypes putatively deleterious (AC or CC), 1 = neutral haplotypes (AA or BC) and 2 = haplotypes putatively protective (AB or BB). Only variants from the chromosome 3 *CCR5-CCR2* genomic region showed nominally significant association with the HIV-1-related outcomes under study. SNP: single nucleotide polymorphism. proxy: high-LD SNP (r2>0.8) that can be used as a tag for the original variant. r2: r-squared. p: p-value.

The *CCR5*-Δ32 variant, a 32 bp deletion in the main HIV-1 co-receptor that protects against HIV-1 acquisition when present in homozygous form [20],[21],[22], strongly associated with both set point (p = 1.7E–10) and progression (p = 3.5E–07). *CCR5*-Δ32 explained 1.7% of the variability in viral control. Only two other variants showed significant associations: The *CCR5* promoter variant P1 [23], found on a haplotype known to increase CCR5 expression [24], associated with higher set point and faster progression, whereas the *CCR2*-64I variant, a Valine to Isoleucine change in the HIV-1 minor receptor CCR2 [25],[26] associated with better viral control (Table 3). They together explained 1% of the variability in viral control. Partial association results for the two *CCR5* variants in a subset of our study population were reported elsewhere [19].

### Effect of identified genetic determinants throughout the full phenotype range

**Figure 4:**
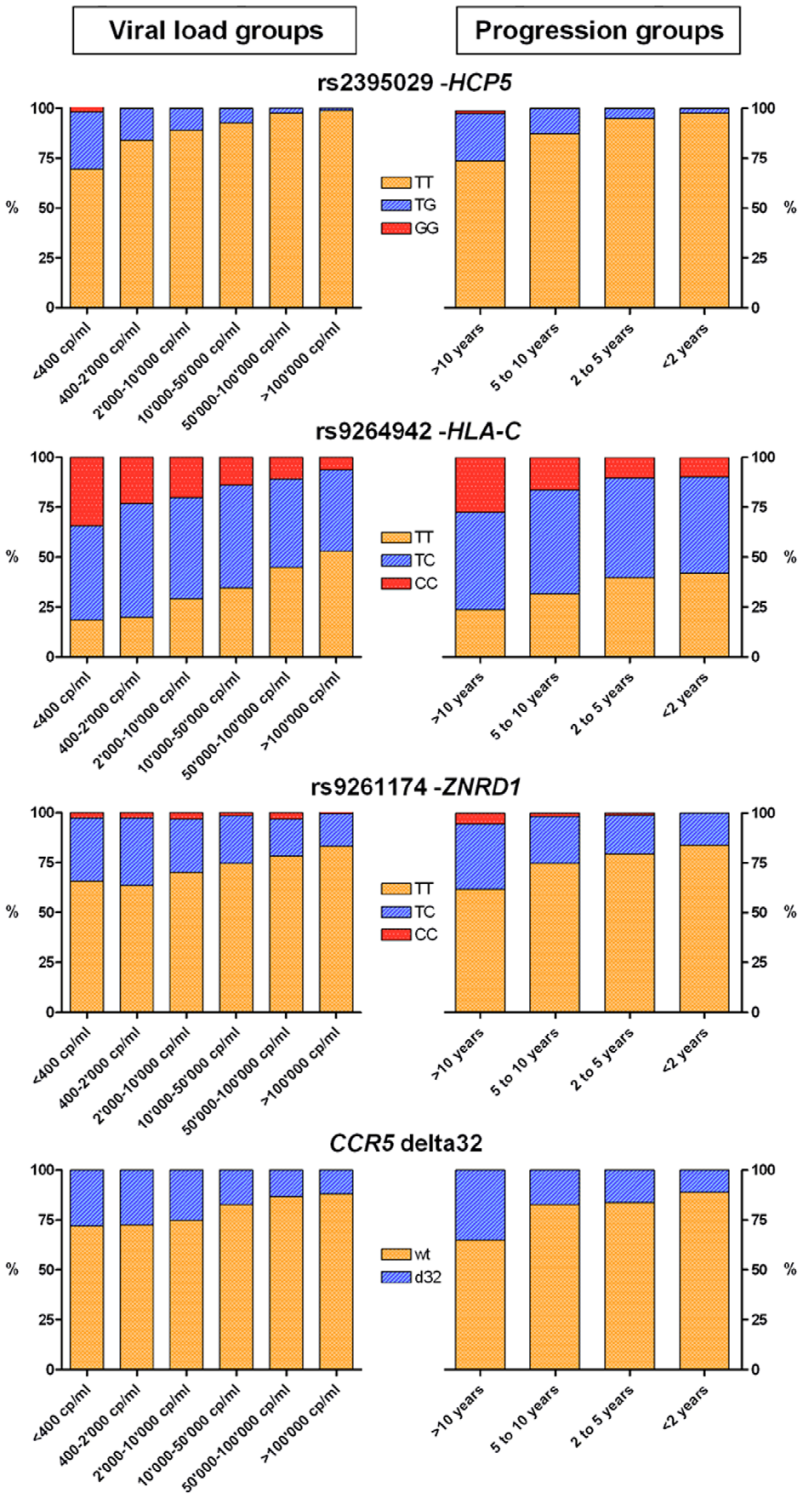
Allelic distribution of the significant variants in subsets of the study population. The bar graphs show the allelic distribution of the 4 variants that have a genome-wide significant association with HIV-1 set point and/or disease progression in subsets of the study population. Groups were defined according to HIV-1 set point (left-hand side graphs) and to progression time (right-hand side graphs).

By design, this study focused on the control of HIV-1 as a quantitative trait, with a particular focus on the amount of virus during the set point period. One important question to address therefore is whether the genetic determinants identified influence viral control throughout the full phenotypic range, from those with low to high viral loads, and from slow to fast progression times. To address this, we looked at allele frequencies in individuals that maintained variable degrees of viral control: we found a consistent enrichment of the protective alleles in categories of subjects with good viral control or slow disease progression rate in comparison to subjects with poor control or rapid progression (Figure 4).

### Additional analyses

The large genotypic and phenotypic data set generated in this study is a resource that allows a more in-depth exploration of the role of human genetic variation: we ran additional analyses to test whether there is any evidence from the existing data, which mainly represents common variants, for other determinants of viral control.

We first performed a genome-wide screen (*Text S1*) for SNPs that modify the effect of rs2395029 (HCP5/B*5701), rs9264942 (*HLA-C*) rs9261174 (*ZNRD1/RNF39*) and *CCR5*-Δ32: no interaction was large enough to reach genome-wide significance. To evaluate common structural variation, we tested 285 SNPs that were identified as tags for copy number polymorphisms (CNP) in HapMap CEU samples [27]. No significant association was observed when theses CNP-tagging SNPs were considered as a set. We also used a gene set enrichment analysis (GSEA) [28],[29] to ask whether groups of genes or pathways were enriched in SNPs with low association p-values: 5 gene sets were significant, some of them with suggestive evidence of involvement in HIV-1 pathogenesis (Table S7). Finally, we developed a permutation procedure to test whether genetic variants with known functional role were more likely to associate with differences in HIV-1 control than non-functional SNPs: we observed a significant effect that was however limited to the MHC region (p = 0.001) (Figure S4).

## Discussion

The results presented here reaffirm the central role of the MHC in HIV-1 control by first confirming with certainty the independence of two association signals in the genomic region that encompasses *HLA-B* and *HLA-C*. These common variants show the strongest association with both viral set point and progression. We initially speculated that the *HCP5* gene itself, which contains the top-associated SNP rs2395029, contributes to HIV-1 control [3], but recent epidemiologic and functional reports [30],[31],[32] suggest that the gene and the variant itself have no such effect. In addition, we here present data from a small number of subjects with a recombination event between *HCP5* and *HLA-B* indicating that HLA-B*5701 is the main contributor to the association signal: the *HCP5* variant is therefore likely to be only a marker for the effect of HLA-B*5701 and possibly of other protective variants present on the same haplotype. The *HLA-C* variant rs9264942, which is in partial LD with all *HLA-C* alleles (Table S6), associates with mRNA and protein expression levels of HLA-C [3],[33] (R. Thomas, M.C., personal communication): it is thus likely that this SNP is in fact a marker of the effect of *HLA-C* expression on HIV-1 control: more work is needed to understand the precise immunological and biological function of HLA-C in the context of HIV-1 infection.

Beyond the top 2 associated variants, we demonstrate that there are other, independent, genetic contributors to HIV-1 control in the MHC. The intricacy of the LD pattern in the region makes it difficult to find causal variants with certainty. Nevertheless, using both SNP and HLA Class I data, we identify additional variants that associate independently with viral set point and together explain at least 3.5% of the variability, on top of the 9% explained by the first 2 SNPs. We do not know at this stage whether any of the 4 SNPs identified in our permutation analysis have a direct functional role, though a non-synonymous coding change in a TRIM gene represents an attractive candidate [34]. Several of the HLA alleles that independently associate with control have good functional support for their involvement in HIV-1 pathogenesis: B*2705 presents epitopes that lead to efficient viral restriction [35], while B*3502 is a member of the B35Px group [36] that, according to recent data obtained on B*3503, has a preferential binding to the inhibitory myelomonocytic MHC class I receptor ILT4 on myeloid dendritic cells, which results in dysfunctional antigen-presenting properties of these cells (XG Yu, personal communication). Functional studies of the gene variants identified in the MHC and deeper understanding of the structure of the associations between SNPs and surrounding HLA alleles [16] are warranted.

We show data that question most previously reported associations in HIV-1 candidate genes (Table 3 and [19]). The chemokine/chemokine receptor locus on chromosome 3 is the only non-MHC region with convincing association evidence for an impact of genetic variation on HIV-1 phenotypes. Homozygosity for CCR5Δ32 is known to confer almost complete protection against infection [20],[21],[22] and we here show that heterozygosity for the 32 bp deletion is the strongest protective factor for VL control and progression outside of MHC. While it is possible that our analyses missed some real candidate gene associations, our failure to replicate most previous reports confirms the critical importance of adopting stringent standards for significance level and stratification control [14],[37],[38].

Our study was powered to detect a single marker association that explains just above 1% of the inter-individual variability in HIV-1 control. In the absence of further significant association at the individual SNP level, we sought to comprehensively assess the impact of common variation by using the genotyping results in several additional ways: these analyses provide no evidence for strong interactions between the MHC or *CCR5* polymorphisms and any other common variant, or for CNP-related effects. We also used a more global approach that shows enrichment for associated variants in some interesting gene sets, but was not designed to identify novel genetic determinants. Limitations to these additional analyses include [1] the deliberately limited scope of our interaction screen, in which only pairs of SNPs including one that is significant have been tested (*Text S1*): we did not run any exhaustive SNP by SNP or haplotype analyses; [2] the use of SNP tags for copy number assessment, which limits the analysis to previously described deletions and duplications that are in LD with common SNPs.

All variants securely identified in this study together explain 13% of the observed variability in HIV-1 viremia in a population of mostly male Caucasian adults. The addition of gender, age and residual population structure to the genetic model pushes this figure up to 22%. These fractions compare favorably to what is known for other complex traits, where dozens of SNPs often explain only a few percent of the variance. Comparable studies are certainly needed in additional populations, notably in other ethnic groups, in women and in children to fully assess the impact of common human genetic variation in HIV-1 control.

Many factors certainly contribute to the large unexplained portion of the inter-individual variability (viral genetics/fitness, environment, stochastic biological variation, noise in phenotype determination), but it is also expected that much more is attributable to human genetic variation. The data presented here suggest that common polymorphisms are unlikely to add much, unless more complex gene by gene and gene-environment interactions play a major role in the genetic architecture of HIV-1 control. After an era of candidate genes studies and a first wave of large-scale genomics projects that could only interrogate common genetic variation, resequencing strategies to identify rare causal variants, as well as integration of multiple genome-level data (genomic DNA, epigenetic marks, transcriptome, siRNA screens) will prove essential to better appreciate the global contribution of the human genome to HIV-1 control.

## Methods

### Ethics statement

All participating centers provided local institutional review board approval for genetic analysis, and each participant provided informed consent for genetic testing.

### Patients/cohorts

Patients have been included from two sources (*Text S1*): [1] Euro-CHAVI, a Consortium of 8 European and 1 Australian Cohorts/Studies; and [2] MACS, the Multicenter AIDS Cohort Study that enrolled homosexual and bisexual men in 4 US cities. In general, patients had viral load (VL) and CD4 count monitoring at least 6 monthly. Eligible patients had 3 or more stable plasma HIV RNA results in the absence of antiretroviral treatment, and met one of the following criteria: a valid seroconversion date estimation proven by documents or biological markers; or, for seroprevalent patients, VL data over a period of at least 3 years, diverging by no more than 0.5 log. Only individuals with known date of seroconversion and at least 2 CD4 T cell determinations in absence of potent antiretroviral treatment (cART) were included in the progression analysis.

### Determination of phenotypes

HIV-1 set point was defined as the average of the remaining VL results after careful assessment of each individual data and elimination of VL outliers: see *Text S1* for criteria used to identify and exclude outlier VL data. Of note, our phenotype definition leads to the exclusion rapid progressors that never reach a stable VL plateau. The disease progression phenotype was defined as [1] the drop of CD4 T cells below 350/ul, or [2] the initiation of cART, but only if the last CD4 T cell count before cART start was <500/ul. This later criterion was made necessary by the different rationales behind treatment initiation between patients and over time: a large part of patients starting cART with CD4 T cell counts approaching the 350/ul threshold did so because their CD4 slope actually showed a significant decrease, whereas most of patients who started treatment with normal or subnormal CD4 T cell counts had stable CD4 T cell profiles. Since progression has been represented in a number of different ways in genetic studies, we used the known genetic determinants to confirm that our measure is the most accurate, by comparing it to survival analyses that used cART start either as a censoring event (i.e. all patients starting cART are considered non-progressors) or as a progression event (i.e. all patients starting cART are considered progressors) (Table S8).

### Genotyping

All participants were genotyped using Illumina BeadChips: We used HumanHap550 Beadchips for 1633 samples and Human1M Beadchips for 921 samples. Most common SNPs found in the HapMap CEU population are readily covered by both chips; however, it is not clear yet whether common variants that have not been genotyped in the HapMap project will be measured equally well. To increase the coverage of the MHC region in the samples genotyped with the 550K chip, we designed a customized MHC-chip that contained an additional 8000 SNPs, largely overlapping with the variants that are present on the Human1M chip. We carried out a series of data cleaning and quality control procedures: SNPs were filtered based on missingness (drop if call rate <99%), MAF (drop if <0.006) and Hardy-Weinberg Equilibrium deviation. Participants were filtered based on call rate, gender check (heterozygosity testing), cryptic relatedness and population structure (see EIGENSTRAT below). The genetic variants located in HIV-1 candidate genes that were not represented directly or indirectly on the genome-wide chips were independently genotyped with TaqMan assays.

### Gender check

The quality control of the genotyping data included a check on the gender specification obtained from the phenotype database, using the observed genotypes of SNPs on chromosome X and Y. Subjects that were identified as “male” in the phenotype file but had a significant amount of heterozygous X genotypes (> = 1%), as well as subjects that was identified as “female” in the phenotype file but had a high frequency of homozygous X genotypes (> = 80%) and Y genotype readings were excluded.

### Control for population stratification

To control for the possibility of spurious associations resulting from population stratification we used a modified EIGENSTRAT approach. This method derives the principal components of the correlations among gene variants and corrects for those correlations in the association tests. In principle therefore the principal components in the analyses should reflect population ancestry. Having noticed however that some of the leading axes depend on other sources of correlation, such as sets of variants near one another that show extended association (LD), we inspected the SNP loadings and followed a series of pruning procedures to ensure that EIGENSTRAT axes reflected only effects that applied equally across the whole genome (*Text S1*).

### Genome-wide association analysis

The core association analyses on HIV-1 setpoint focused on single-marker genotype-trend tests of the quality control–passed SNPs using linear regression and including age, gender, and the significant EIGENSTRAT axes values as covariates. Associations with progression were tested using a Cox proportional hazards model. We assessed significance with a Bonferroni correction taking into account 1 million tests (p-value cutoff  =  5E–08).

### Search for independent associations in the MHC

To search for additional SNPs effects in the MHC region we used a forward selection algorithm to investigate all MHC SNPs with p<1E–04 (N = 331). The top 2 associated variants and the standard covariates were fixed in a linear regression model, while new SNPs were added and selected into the model one at a time. In the forward selection process, a newly selected SNP is expected to explain a certain proportion of the set point variation independently of all variables already in the model. To control for multiple testing, a permutation procedure was used to assess the empirical significance cut-off value. In the permutation, the LD patterns among SNPs were retained while the associations between SNPs and set point were permuted. 1000 permutation runs were performed and the 5^th^ percentile of the empirical distributions of the partial R-squares and the p-values of the 1^st^, 2^nd^, 3^rd^, etc. selected SNPs were recorded and compared to the observed sample statistics. The permutation p-value was defined as the probability that the statistics observed in the permutation was more extreme than the statistics observed in the real sample. The forward selection algorithm is expected to be conservative. To verify this, a large-scale statistical simulation was performed, considering different effect sizes, various LD patterns among SNPs and different sample sizes (*Text S1*). The simulation results confirmed that the forward selection algorithm with permutation procedure is conservative.

### Interaction analysis

We performed a two-way interaction test between each QC-passed SNP and the top associated variants. For each genome-wide significant polymorphism, the screening procedure involved the calculation of the p-value associated with each of the ∼1 million multivariate linear models incorporating the 2 polymorphisms. The p-value associated with the interaction term for this model was retained and then the top p-values for all ∼1 million tests considered. This approach followed recommendations that the search space for interactions can be appropriately reduced and therefore the power can be improved by focusing the search on pairs of polymorphisms including at least one that is known to be significant [39].

### Gene set enrichment analysis (GSEA)

The SNPs that were represented in all genotyping chips were mapped to their closest gene (if <500 kb away) and used in the GSEA analysis if minor allele frequency was >0.05, Hardy-Weinberg Equilibrium test p-value was >0.001, and at least 90% individuals were successfully genotyped. Among the 639 canonical pathway gene sets that are represented in the Molecular Signatures Database (MsigDB, http://www.broad.mit.edu/gsea/msigdb/collections.jsp), we tested the 298 gene sets that had at least 20 and at most 200 genes represented in the study (*Text S1*).

### Functional variants

A permutation procedure was developed to test whether genetic variants with known functional role were more likely to have a lower p-values distribution than supposedly neutral variants (*Text S1*). We compared the overall p-value distribution of 12,535 putatively functional polymorphisms, including stop-gained, stop-lost, frame-shift coding, non-synonymous coding, and essential splicing site genetic variants, with the global distribution of p-values of all genotyped SNPs (with 10,000 permutations). We also ran the same analysis for the MHC region only (318 functional variants) and for the rest of the genome (12217 functional variants).

## Supporting Information

Figure S1 Distribution of individual eigen values along the first axis identified by principal component analysis of the genotyping data (Eigenstrat). Participants are grouped by country of recruitment. The most important contributor to population stratification in our Caucasian population is a North-to-South European ancestry axis. Participants from the USA and Australia are most similar to northern Europeans.
(0.05 MB DOC)

Figure S2 QQ-plot of observed versus expected -log(p-values) for all SNPs in the set point association analysis. The plot shows no deviation from the expected line, except for the top 3000 SNPs (0.3%).
(0.03 MB DOC)

Figure S3 Associations between the SNPs identified in the *ZNRD1/RNF39* region and disease progression in individuals with and without HLA-A alleles belonging to the serogroup A10. The rs9261174 minor allele C shows the strongest association with progression in individuals that also have an HLA-A10 (green), but it also associates when HLA-A10 is absent (red). The LD (r2) between the *ZNRD1* SNP rs9261174 and HLA-A10 as a group is 0.46. It is 0.17 for HLA-A*2501, 0.25 for A*2601, 0.00 for A*3402 and 0.02 for A*6601.
(0.06 MB DOC)

Figure S4 P-value distribution of functional variants. P-value distributions of 12,535 functional genetic variants (red, A) in comparison with 12,535 randomly selected intergenic variants (red, B), both in the background of p-value distributions generated from 10,000 permutations (blue, A and B). The upper figures show the distributions of ranked -log10(P) for each of the observed and permutated p-value series. The lower figures show the distributions of the sum -log10(P) generated from the permutated series (blue), their 95% cutoff of empirical probability (cyan), and the relative position and probability of the observed sum -log10(P) (red) in these distributions. The sum -log10(P) from the observed functional genetic variants (red, A) is higher than what would be expected if there were no enrichment of low p-values in this series, and this phenomenon is very unlikely to be due to chance (p = 0.004), while the data from randomly selected 12,535 intergenic variants shows no difference (p = 0.76). The randomly selected 12,535 intergenic variant series in (B) can be approximately viewed as one of the permutated data series in (A) except that they are restricted to be annotated as intergenic, and is presented here for better illustration of the results. Functional genetic variants are those annotated as falling into one or more of these categories: stop-gained, stop-lost, fame-shift coding, non-synonymous coding, and essential splicing site genetic variants. Ensembl database version 50_36i was used for these annotations.
(0.13 MB DOC)

Table S1 Participants included in the study.
(0.04 MB DOC)

Table S2 VL setpoint values for groups of individuals with or without a recombination event between *HCP5* and *HLA-B*.
(0.03 MB DOC)

Table S3 Results of the stepwise forward selection of MHC SNPs.
(0.04 MB DOC)

Table S4 Associations between 4-digit HLA Class I alleles and HIV-1 set point in the subset of 1204 subjects with complete SNP and HLA typing results.
(0.10 MB DOC)

Table S5 Pairs of *HLA-B* and *HLA-C* alleles that are in linkage disequilibrium (with an r2 of at least 0.1) in the subset of 1204 individuals with complete HLA typing results.
(0.07 MB DOC)

Table S6 Associations between *HLA-C* alleles and HIV-1 set point in the subset of 1204 subjects with complete SNP and HLA typing results.
(0.06 MB DOC)

Table S7 Results of the GSEA analysis.
(0.04 MB DOC)

Table S8 Comparison of the strength of the association results for polymorphisms with clear association with HIV outcomes using different definitions of progression in survival analyses.
(0.03 MB DOC)

Table S9 Top 500 SNPs in the setpoint analysis.
(0.74 MB DOC)

Table S10 Top 500 SNPs in the progression analysis.
(0.72 MB DOC)

Table S11 Number of SNPs discarded during quality control procedures.
(0.03 MB DOC)

Text S1 Supplementary text.
(0.09 MB DOC)
