# Supplementary figures and images for: Common Genetic Variation and the Control of HIV-1 in Humans
Source: PLoS Genet. 2009 Dec 24;5(12):e1000791. doi: 10.1371/journal.pgen.1000791 (PMC2791220; doi:10.1371/journal.pgen.1000791)

**
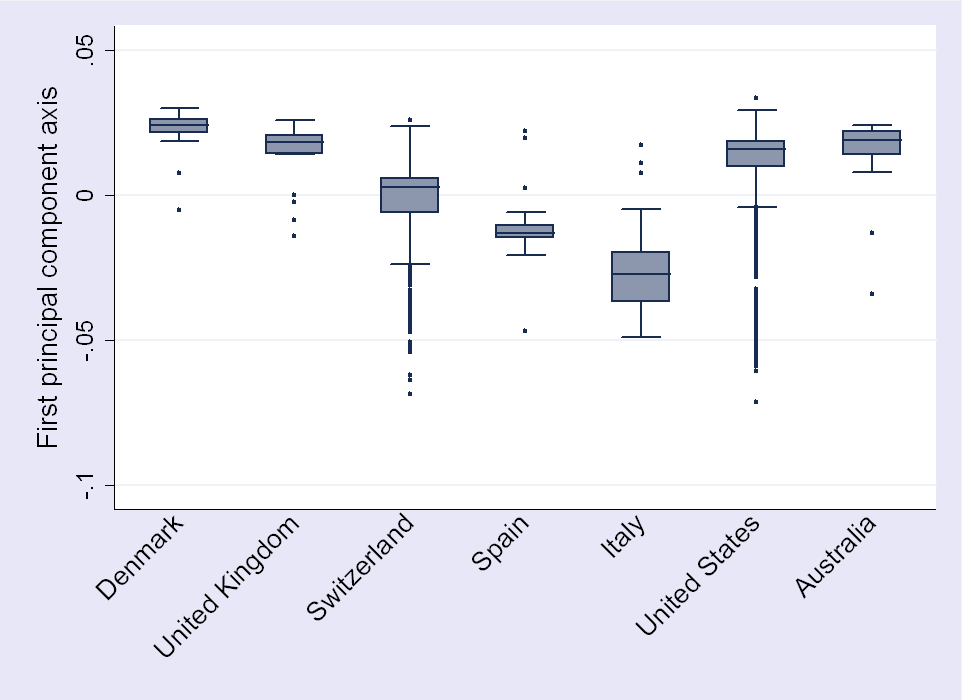
**

Supplement: Figure S1 — Distribution of individual eigen values along the first axis identified by principal component analysis of the genotyping data (Eigenstrat). Participants are grouped by country of recruitment. The most important contributor to population stratification in our Caucasian population is a North-to-South European ancestry axis. Participants from the USA and Australia are most similar to northern Europeans. (0.05 MB DOC) [file pgen.1000791.s001.doc]

**
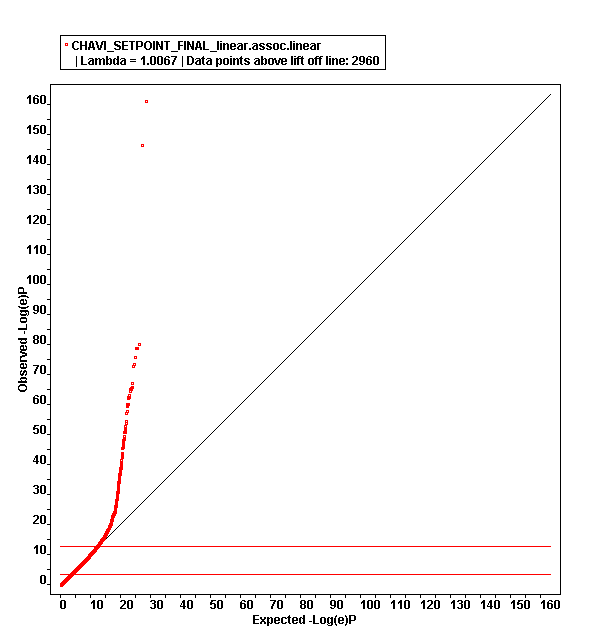
**

Supplement: Figure S2 — QQ-plot of observed versus expected -log(p-values) for all SNPs in the set point association analysis. The plot shows no deviation from the expected line, except for the top 3000 SNPs (0.3%). (0.03 MB DOC) [file pgen.1000791.s002.doc]

**
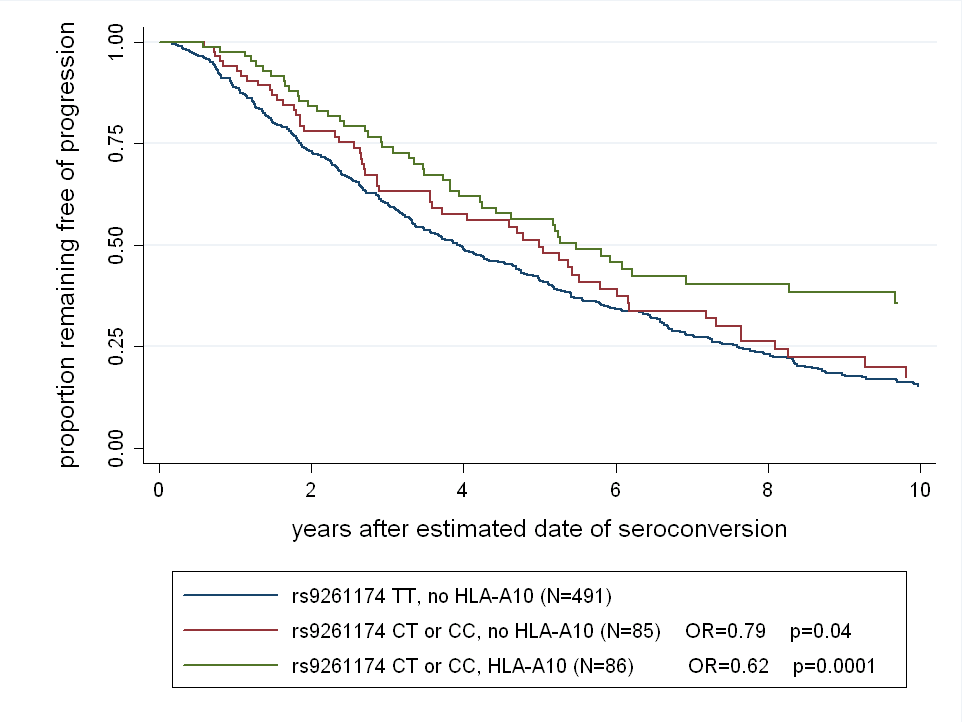
**

Supplement: Figure S3 — Associations between the SNPs identified in the ZNRD1/RNF39 region and disease progression in individuals with and without HLA-A alleles belonging to the serogroup A10. The rs9261174 minor allele C shows the strongest association with progression in individuals that also have an HLA-A10 (green), but it also associates when HLA-A10 is absent (red). The LD (r2) between the ZNRD1 SNP rs9261174 and HLA-A10 as a group is 0.46. It is 0.17 for HLA-A*2501, 0.25 for A*2601, 0.00 for A*3402 and 0.02 for A*6601. (0.06 MB DOC) [file pgen.1000791.s003.doc]

**
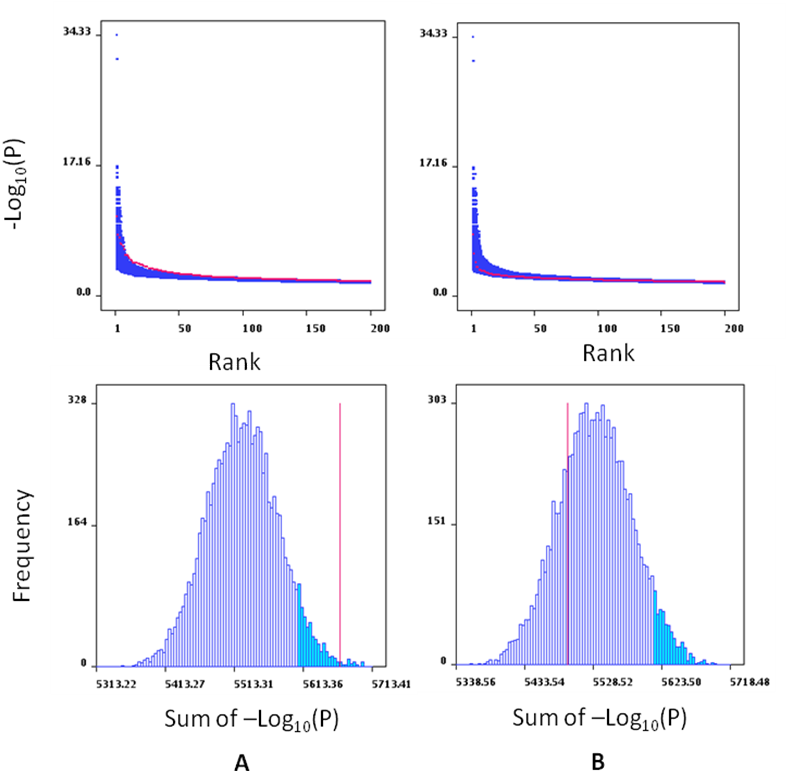
**

Supplement: Figure S4 — P-value distribution of functional variants. P-value distributions of 12,535 functional genetic variants (red, A) in comparison with 12,535 randomly selected intergenic variants (red, B), both in the background of p-value distributions generated from 10,000 permutations (blue, A and B). The upper figures show the distributions of ranked -log10(P) for each of the observed and permutated p-value series. The lower figures show the distributions of the sum -log10(P) generated from the permutated series (blue), their 95% cutoff of empirical probability (cyan), and the relative position and probability of the observed sum -log10(P) (red) in these distributions. The sum -log10(P) from the observed functional genetic variants (red, A) is higher than what would be expected if there were no enrichment of low p-values in this series, and this phenomenon is very unlikely to be due to chance (p = 0.004), while the data from randomly selected 12,535 intergenic variants shows no difference (p = 0.76). The randomly selected 12,535 intergenic variant series in (B) can be approximately viewed as one of the permutated data series in (A) except that they are restricted to be annotated as intergenic, and is presented here for better illustration of the results. Functional genetic variants are those annotated as falling into one or more of these categories: stop-gained, stop-lost, fame-shift coding, non-synonymous coding, and essential splicing site genetic variants. Ensembl database version 50_36i was used for these annotations. (0.13 MB DOC) [file pgen.1000791.s004.doc]
