# Supplementary material for: Common Genetic Variation and the Control of HIV-1 in Humans
Source: PLoS Genet. 2009 Dec 24;5(12):e1000791. doi: 10.1371/journal.pgen.1000791 (PMC2791220; doi:10.1371/journal.pgen.1000791)
Supplement: Table S1 — Participants included in the study. (0.04 MB DOC) [file pgen.1000791.s005.doc]

**Table S1**: Participants included in the study

**A. Characteristics of study participants, by cohort**

|  |  | Euro-CHAVI (N=1397) | MACS  (N=1157) | Total  (N=2554) |
| --- | --- | --- | --- | --- |
| Gender [N (%)] | Male | 1057 (75.7) | 1157 (100) | 2214 (86.7) |
|  | Female | 340 (24.3) | 0 | 340 (13.3) |
| Age at seroconversion [median (IQR)] |  | 33 (27-39) | 33 (28-38) | 33 (28-39) |
| Mode of HIV infection [N (%)] | Homosexual | 626 (44.8) | 1157 (100) | 1783 (69.8) |
|  | Heterosexual | 431 (30.8) | 0 | 431 (16.9) |
|  | Intravenous drug use | 286 (20.5) | 0 | 286 (11.2) |
|  | Other /unknown | 54 (3.9) | 0 | 54 (2.1) |
| Inclusion criteria [N (%)] | Proven seroconversion | 675 (48.4) | 438 (37.9) | 1113 (43.6) |
|  | Stable viral load | 722 (51.6) | 719 (62.1) | 1441 (56.4) |
| Excluded during quality control [N (%)] |  | 117 (8.4) | 75 (6.5) | 192 (7.5) |

**B. Participants included in the association analyses and genotyping chips that were used**

|  |  | Euro-CHAVI | MACS | Total |
| --- | --- | --- | --- | --- |
| Included in setpoint analysis [N] |  | 1280 | 1082 | 2362 |
| Included in progression analysis [N] |  | 634 | 437 | 1071 |
| Genotyping chip used [N (%)] | 550k | 1147 (89.6) | 358 (33.1) | 1505 (63.7) |
|  | 1M | 133 (10.4) | 724 (66.9) | 857 (36.3) |
|  | HLA chip | 847 (66.2) | 323 (29.9) | 1170 (49.5) |

550k: Illumina HumanHap550 Beadchip

1M: Illumina Human1M Beadchip

HLA chip: customized MHC-chip containing 8000 SNPs, largely overlapping with the variants that are present on the Human1M Beadchip
