# Supplementary material for: Common Genetic Variation and the Control of HIV-1 in Humans
Source: PLoS Genet. 2009 Dec 24;5(12):e1000791. doi: 10.1371/journal.pgen.1000791 (PMC2791220; doi:10.1371/journal.pgen.1000791)
Supplement: Table S2 — VL setpoint values for groups of individuals with or without a recombination event between HCP5 and HLA-B. (0.03 MB DOC) [file pgen.1000791.s006.doc]

**Table S2:** VL setpoint values for groups of individuals with or without a recombination event between *HCP5* and *HLA-B*

| rs2395029 minor allele C | HLA-B*5701 | N | mean | SD | median | IQR |
| --- | --- | --- | --- | --- | --- | --- |
| Yes | No | 5 | 4.06 | 0.57 | 4.22 | 4.14-4.28 |
| No | Yes | 4 | 3.22 | 0.68 | 3.14 | 2.77-3.68 |
| No | No | 1078 | 4.22 | 0.83 | 4.35 | 3.86-4.77 |
| Yes | Yes | 117 | 3.51 | 0.98 | 3.77 | 2.84-4.22 |

A break in the strong linkage disequilibrium between *HCP5* and *HLA-B* was observed in 9/1204 subjects with HLA Class I results (0.7%, r2=0.93): the set point values were lower for the 4 patients that had B*5701 without the rs2395029 minor allele than for the 5 patients with a G at rs2395029 but without B*5701 (p=0.05, Kruskal-Wallis rank test). For comparison, Setpoint values are also shown for groups of individuals with and without the conserved protective haplotype consisting of rs2395029 minor allele C and HLA-B*5701.

N: number of individuals in each group

SD: standard deviation

IQR: interquartile range
