# Supplementary material for: Common Genetic Variation and the Control of HIV-1 in Humans
Source: PLoS Genet. 2009 Dec 24;5(12):e1000791. doi: 10.1371/journal.pgen.1000791 (PMC2791220; doi:10.1371/journal.pgen.1000791)
Supplement: Table S3 — Results of the stepwise forward selection of MHC SNPs. (0.04 MB DOC) [file pgen.1000791.s007.doc]

**Table S3**: Results of the stepwise forward selection of MHC SNPs.

**A.**

| **Covariates in model** | **rs259919** | **rs9468692** | **rs9266409** | **rs8192591** |
| --- | --- | --- | --- | --- |
| Gender, age, 12 Eigenstrat axes | 5.3E-04 | 7.6E-04 | 4.9E-14 | 9.0E-09 |
| + rs2395029 | 6.3E-09 | 2.3E-03 | 9.1E-04 | 2.3E-04 |
| + rs9264942 | 3.0E-07 | 3.0E-05 | 5.1E-05 | 3.9E-05 |
| + rs259919 |  | 1.1E-06 | 9.1E-05 | 6.5E-05 |
| + rs9468692 |  |  | 3.6E-05 | 4.9E-05 |
| +rs9266409 |  |  |  | 5.5E-05 |

B.

| **Markers** | **R-square** | **P (permutation)** |
| --- | --- | --- |
| Basic model* | 0.1598 |  |
| rs259919 | 0.1682 | 0.000 |
| rs9468692 | 0.1749 | 0.003 |
| rs9266409 | 0.1816 | 0.003 |
| rs8192591 | 0.1869 | 0.012 |

P-values for association with HIV-1 viral load at set point for the 4 variants that showed an independent association signal in the stepwise forward selection of MHC SNPs (**A**). The 4 highlighted p-values represent the independent association of each of the 4 SNPs with the phenotype in combined models: they are not strictly genome-wide significant, but in our permutation analysis (1000 permutation runs were performed), they had p-values that were less than the 5^th^ percentile from all permuted p-values (**B**), showing that the variants actually add something to the previous model. R-square represents the fraction of the variability in the phenotype that is explained by the model. *****Basic model contains gender, age, 12 Eigenstrat axes, as well as rs2395029 and rs9264942 as covariates.
