# Supplementary material for: Common Genetic Variation and the Control of HIV-1 in Humans
Source: PLoS Genet. 2009 Dec 24;5(12):e1000791. doi: 10.1371/journal.pgen.1000791 (PMC2791220; doi:10.1371/journal.pgen.1000791)
Supplement: Table S4 — Associations between 4-digit HLA Class I alleles and HIV-1 set point in the subset of 1204 subjects with complete SNP and HLA typing results. (0.10 MB DOC) [file pgen.1000791.s008.doc]

**Table S4.** Associations between 4-digit HLA Class I alleles and HIV-1 set point in the subset of 1204 subjects with complete SNP and HLA typing results.

| **HLA allele** | ***N*** | **set point VL (mean)** | **p-value^#^** | **p-value^#^ in a model including rs2395029 and rs9264942** |
| --- | --- | --- | --- | --- |
| **A*0101** | *343* | 4.18 | 0.5 | 0.07 |
| **A*0201** | *566* | 4.15 | 0.2 | 0.04 |
| **A*0301** | *315* | 4.16 | 0.7 | 0.4 |
| **A*0302** | *7* | 3.50 | 0.09 | 0.05 |
| **A*1101** | *165* | 4.06 | 0.1 | 0.01 |
| **A*2301** | *55* | 4.30 | 0.3 | 0.5 |
| **A*2402** | *204* | 4.25 | 0.03 | 0.1 |
| **A*2501** | *60* | 3.88 | 0.03 | 0.1 |
| **A*2601** | *94* | 4.00 | 0.2 | 0.6 |
| **A*2901** | *9* | 4.29 | 0.4 | 0.5 |
| **A*2902** | *69* | 4.35 | 0.1 | 0.4 |
| **A*3001** | *32* | 4.06 | 0.9 | 0.9 |
| **A*3002** | *11* | 4.33 | 0.5 | 0.2 |
| **A*3101** | *68* | 3.96 | 0.05 | 0.03 |
| **A*3201** | *96* | 3.95 | 0.005 | 0.006 |
| **A*3301** | *22* | 3.64 | 0.02 | 0.06 |
| **A*6601** | *13* | 4.36 | 0.2 | 0.3 |
| **A*6801** | *66* | 4.13 | 0.7 | 0.9 |
| **A*6901** | *6* | 4.34 | 0.5 | 0.6 |
| **B*0702** | *292* | 4.26 | 0.007 | 0.4 |
| **B*0801** | *224* | 4.31 | 0.007 | 0.6 |
| **B*1302** | *63* | 3.74 | 0.002 | 0.01 |
| **B*1401** | *30* | 3.87 | 0.07 | 0.3 |
| **B*1402** | *69* | 3.87 | 0.05 | 0.2 |
| **B*1501** | *155* | 4.11 | 0.6 | 0.02 |
| **B*1801** | *110* | 4.17 | 0.4 | 0.2 |
| **B*2702** | *13* | 3.97 | 0.7 | 0.8 |
| **B*2705** | *105* | 3.79 | 0.00005 | 0.002 |
| **B*3501** | *136* | 4.20 | 0.2 | 0.7 |
| **B*3502** | *30* | 4.68 | 0.00002 | 0.0006 |
| **B*3503** | *66* | 4.19 | 0.3 | 0.8 |
| **B*3701** | *46* | 4.29 | 0.4 | 0.09 |
| **B*3801** | *64* | 4.29 | 0.07 | 0.004 |
| **B*3901** | *37* | 4.06 | 0.7 | 0.7 |
| **B*4001** | *114* | 4.33 | 0.05 | 0.6 |
| **B*4002** | *47* | 3.87 | 0.1 | 0.2 |
| **B*4101** | *11* | 4.33 | 0.6 | 1.0 |
| **B*4102** | *9* | 4.19 | 0.6 | 1.0 |
| **B*4402** | *214* | 4.19 | 0.1 | 0.001 |
| **B*4403** | *121* | 4.12 | 0.7 | 0.3 |
| **B*4501** | *8* | 4.63 | 0.2 | 0.1 |
| **B*4701** | *11* | 4.02 | 0.9 | 0.6 |
| **B*4901** | *44* | 4.30 | 0.3 | 0.7 |
| **B*5001** | *30* | 4.08 | 0.9 | 0.2 |
| **B*5101** | *168* | 4.03 | 0.3 | 0.1 |
| **B*5201** | *32* | 3.74 | 0.06 | 0.05 |
| **B*5301** | *16* | 4.19 | 0.8 | 0.8 |
| **B*5501** | *35* | 4.37 | 0.2 | 0.7 |
| **B*5601** | *17* | 4.65 | 0.04 | 0.2 |
| **B*5701** | *138* | 3.52 | 4E-19 | † |
| **B*5801** | *22* | 4.09 | 0.7 | 0.9 |
| **C*0102** | *106* | 4.00 | 0.3 | 0.2 |
| **C*0202** | *135* | 3.85 | 0.001 | 0.06 |
| **C*0302** | *26* | 4.35 | 0.06 | 0.08 |
| **C*0303** | *133* | 4.25 | 0.2 | 1.0 |
| **C*0304** | *158* | 4.20 | 0.6 | 0.3 |
| **C*0401** | *293* | 4.23 | 0.005 | 0.9 |
| **C*0501** | *198* | 4.21 | 0.2 | 0.001 |
| **C*0602** | *279* | 3.86 | 1E-11 | 0.7 |
| **C*0701** | *339* | 4.28 | 0.0005 | 0.2 |
| **C*0702** | *322* | 4.24 | 0.03 | 1.0 |
| **C*0802** | *95* | 3.92 | 0.01 | 0.2 |
| **C*1202** | *30* | 3.70 | 0.2 | 0.3 |
| **C*1203** | *161* | 4.03 | 0.3 | 0.9 |
| **C*1402** | *42* | 3.89 | 0.2 | 0.3 |
| **C*1502** | *67* | 4.00 | 0.5 | 0.09 |
| **C*1601** | *76* | 4.22 | 0.5 | 0.6 |
| **C*1602** | *11* | 4.13 | 0.7 | 0.8 |
| **C*1701** | *18* | 4.41 | 0.3 | 0.6 |

Only HLA Class I alleles that were observed in more than 5 individuals are included (with the exception of B*5701, discussed in the text).

^#^ All linear regression models include gender, age and the 12 Eigenstrat axes as covariates.

† Not calculated because of co-linearity between rs2395029 and HLA-B*5701.
