# Supplementary material for: Common Genetic Variation and the Control of HIV-1 in Humans
Source: PLoS Genet. 2009 Dec 24;5(12):e1000791. doi: 10.1371/journal.pgen.1000791 (PMC2791220; doi:10.1371/journal.pgen.1000791)
Supplement: Table S5 — Pairs of HLA-B and HLA-C alleles that are in linkage disequilibrium (with an r2 of at least 0.1) in the subset of 1204 individuals with complete HLA typing results. (0.07 MB DOC) [file pgen.1000791.s009.doc]

**Table S5**: Pairs of *HLA-B* and *HLA-C* alleles that are in linkage disequilibrium (with an r2 of at least 0.1) in the subset of 1204 individuals with complete HLA typing results.

| **HLA-B allele** | ***MAF (%)*** | **HLA-C allele** | ***MAF (%)*** | **r2** |
| --- | --- | --- | --- | --- |
| 0702 | *11.4* | **0702** | *12.7* | **0.80** |
| 0705 | *0.4* | **1505** | *0.5* | **0.56** |
| 0801 | *8.8* | **0701** | *13.8* | **0.62** |
| 1302 | *2.4* | **0602** | *11.1* | **0.15** |
| 1401 | *1.1* | **0802** | *3.7* | **0.25** |
| 1402 | *2.7* | **0802** | *3.7* | **0.61** |
| 1501 | *5.9* | **0303** | *4.9* | **0.31** |
| 1801 | *4.2* | **1203** | *6.1* | **0.10** |
| 2705 | *4.1* | **0102** | *4.1* | **0.17** |
| 2705 | *4.1* | **0202** | *5.2* | **0.18** |
| 3501 | *5.3* | **0401** | *11.6* | **0.38** |
| 3503 | *2.5* | **0401** | *11.6* | **0.11** |
| 3701 | *1.7* | **0602** | *11.1* | **0.13** |
| 3801 | *2.4* | **1203** | *6.1* | **0.32** |
| 4001 | *4.3* | **0304** | *6.1* | **0.49** |
| 4002 | *1.8* | **0202** | *5.2* | **0.11** |
| 4101 | *0.4* | **1701** | *0.7* | **0.28** |
| 4102 | *0.3* | **1701** | *0.7* | **0.41** |
| 4402 | *8.3* | **0501** | *7.6* | **0.60** |
| 4403 | *4.7* | **1601** | *2.8* | **0.44** |
| 4901 | *1.7* | **0701** | *13.8* | **0.11** |
| 5101 | *6.6* | **1402** | *1.5* | **0.18** |
| 5101 | *6.6* | **1502** | *2.7* | **0.21** |
| 5201 | *1.2* | **1202** | *1.2* | **0.85** |
| 5501 | *1.3* | **0303** | *4.9* | **0.15** |
| 5601 | *0.7* | **0102** | *4.1* | **0.17** |
| 5701 | *5.3* | **0602** | *11.1* | **0.41** |
| 5801 | *0.7* | **0302** | *1.0* | **0.18** |

The HLA-B and HLA-C genes represent a very tight two-locus haplotype: certain B and C alleles are therefore in significant LD and reflect at least partially the same signal in association studies.
