# Supplementary material for: Common Genetic Variation and the Control of HIV-1 in Humans
Source: PLoS Genet. 2009 Dec 24;5(12):e1000791. doi: 10.1371/journal.pgen.1000791 (PMC2791220; doi:10.1371/journal.pgen.1000791)
Supplement: Table S6 — Associations between HLA-C alleles and HIV-1 set point in the subset of 1204 subjects with complete SNP and HLA typing results. (0.06 MB DOC) [file pgen.1000791.s010.doc]

**Table S6:** Associations between *HLA-C* alleles and HIV-1 set point in the subset of 1204 subjects with complete SNP and HLA typing results.

| **HLA-C allele** | **Frequency** | **rs9264942 allele** | **r2** | **p (LD)** | **p (setpoint)** | **p with rs9264942** | **p with rs2395029, rs9264942** | **p with rs2395029, rs9264942, HLA-B*27** |
| --- | --- | --- | --- | --- | --- | --- | --- | --- |
| ***0102** | 0.041 | **C** | 0.01 | 0.005 | 0.27 | 0.70 | 0.24 | 0.49 |
| ***0202** | 0.052 | **C** | 0.08 | <0.0001 | 1.0E-03 | 0.18 | 0.058 | 0.32 |
| ***0501** | 0.076 | **C** | 0.12 | <0.0001 | 0.21 | 3.87E-06 | 1.01E-03 | 0.01 |
| ***0602** | 0.111 | **C** | 0.19 | <0.0001 | 1.18E-11 | 2.53E-04 | 0.71 | 0.79 |
| ***0802** | 0.037 | **C** | 0.06 | <0.0001 | 0.01 | 0.62 | 0.16 | 0.09 |
| ***all Cw8*** | *0.039* | ***C*** | *0.06* | *<0.0001* | *0.01* | *0.58* | *0.16* | *0.08* |
| ***1202** | 0.012 | **C** | 0.01 | <0.0001 | 0.19 | 0.58 | 0.27 | 0.20 |
| ***1203** | 0.061 | **C** | 0.08 | <0.0001 | 0.26 | 0.27 | 0.86 | 0.77 |
| ***all Cw12*** | *0.074* | ***C*** | *0.10* | *<0.0001* | *0.05* | *0.70* | *0.46* | *0.51* |
| ***1402** | 0.015 | **C** | 0.02 | <0.0001 | 0.16 | 0.65 | 0.31 | 0.08 |
| ***0303** | 0.049 | **T** | 0.02 | <0.0001 | 0.18 | 0.90 | 0.97 | 0.45 |
| ***0304** | 0.061 | **T** | 0.04 | <0.0001 | 0.57 | 0.93 | 0.29 | 0.21 |
| ***all Cw3*** | *0.119* | ***T*** | *0.06* | *<0.0001* | *0.06* | *0.35* | *0.81* | *0.85* |
| ***0401** | 0.116 | **T** | 0.11 | <0.0001 | 4.6E-03 | 0.85 | 0.93 | 0.85 |
| ***0701** | 0.138 | **T** | 0.10 | <0.0001 | 5.39E-04 | 0.22 | 0.18 | 0.14 |
| ***0702** | 0.127 | **T** | 0.09 | <0.0001 | 0.03 | 0.76 | 0.99 | 0.93 |
| ***0704** | 0.014 | **T** | 0.01 | <0.0001 | 0.37 | 0.99 | 0.82 | 0.69 |
| ***all Cw7*** | *0.280* | ***T*** | *0.25* | *<0.0001* | *7.45-06* | *0.46* | *0.25* | *0.27* |
| ***1502** | 0.027 | **T** | 0.01 | <0.0001 | 0.54 | 0.13 | 0.09 | 0.13 |
| ***all Cw15*** | *0.032* | ***T*** | *0.02* | *<0.0001* | *0.49* | *0.10* | *0.07* | *0.11* |
| ***1601** | 0.028 | **T** | 0.02 | <0.0001 | 0.46 | 0.82 | 0.61 | 0.52 |
| ***all Cw16*** | *0.035* | ***T*** | *0.02* | *<0.0001* | *0.68* | *0.49* | *0.31* | *0.21* |
| ***1701** | 0.007 | **T** | 0.01 | 0.05 | 0.27 | 0.54 | 0.63 | 0.88 |

The *HLA-C* alleles can be perfectly divided in 2 groups according to LD with the C or the T allele of the *HLA-C* -35 SNP rs9264942: the ‘r2’ and ‘p (LD)’ columns indicate the strength of LD between *HLA-C* alleles and -35 SNP genotype. Significant p-values for association between *HLA-C* alleles and set point are shown in blue: most of the significant signals disappear after adjustment for the top associated SNPs. All models include gender, age and 12 Eigenstrat axes as covariates. All Cw3 = *0302, *0303, *0304; all Cw7 = *0701, *0702, *0704, *0716; all Cw8 = *0801, *0802, *0804; all Cw12 = *1202, *1203; all Cw15 = *1502, *1504, *1505; all Cw16 = *1601, *1602, *1604.
