# Supplementary material for: Common Genetic Variation and the Control of HIV-1 in Humans
Source: PLoS Genet. 2009 Dec 24;5(12):e1000791. doi: 10.1371/journal.pgen.1000791 (PMC2791220; doi:10.1371/journal.pgen.1000791)
Supplement: Table S7 — Results of the GSEA analysis. (0.04 MB DOC) [file pgen.1000791.s011.doc]

**Table S7:** Results of the GSEA analysis.

| **Gene set** | **Number of SNPs** | **Number of genes** | **Top SNP (rank)** | **Top gene (rank, chromosome)** | **Nominal p** | **FDR**  **q** |
| --- | --- | --- | --- | --- | --- | --- |
| Hsa00624 1 and 2 methylnaphthalene  degradation | 302 | 13 / 22 / 25 * | rs1680777 (1584^th^) | ACAD9  (784^th^, 3) | 0.006 | 0.17 |
|  |  |  |  |  |  |  |
| Hsa04514 cell adhesion  molecules | 5918 | 74 / 121 / 134 | rs9264942 (1^st^) | HLA-C  (1^st^, 6) | 0.003 | 0.20 |
|  |  |  |  |  |  |  |
| galactose metabolism | 477 | 13 / 22 / 24 | rs13225343 (614^th^) | AKR1B1  (307^th^, 7) | 0.003 | 0.20 |
|  |  |  |  |  |  |  |
| inflampathway | 599 | 12 / 28 / 29 | rs3093662 (11^th^) | TNF  (6^th^, 6) | 0.006 | 0.22 |
|  |  |  |  |  |  |  |
| cskpathway | 393 | 11 / 22 / 24 | rs7356880 (98^th^) | HLA-DRA  (31^st^, 6) | 0.009 | 0.24 |

* 13 genes with p < 0.05, 22 genes with SNP(s) mapped, total 25 genes in the gene set.

5 gene sets had an FDR q-value < 0.25. The genes present in these gene sets were enriched in SNPs with low p-values in the set point association analysis.

hsa00624 1 & 2 methylnaphthalene degradation: <http://www.kegg.jp/dbget-bin/show_pathway?HSA00624>

hsa04514 cell adhesion molecules: <http://www.kegg.jp/dbget-bin/show_pathway?HSA04514>

galactose metabolism: <http://www.genome.jp/kegg/pathway/map/map00052.html>

inflampathway: <http://www.biocarta.com/pathfiles/h_INFLAMPATHWAY.asp>

cskpathway: <http://www.biocarta.com/pathfiles/h_CSKPATHWAY.asp>
