# Supplementary material for: Common Genetic Variation and the Control of HIV-1 in Humans
Source: PLoS Genet. 2009 Dec 24;5(12):e1000791. doi: 10.1371/journal.pgen.1000791 (PMC2791220; doi:10.1371/journal.pgen.1000791)
Supplement: Table S8 — Comparison of the strength of the association results for polymorphisms with clear association with HIV outcomes using different definitions of progression in survival analyses. (0.03 MB DOC) [file pgen.1000791.s012.doc]

**Table S8:** Comparison of the strength of the association results for polymorphisms with clear association with HIV outcomes using different definitions of progression in survival analyses.

| ***Genetic variant*** | ***MAF*** | ***cART start ⬄***  ***Censoring event*** | | ***cART start ⬄ Progression event*** | | ***cART start ⬄ Progression if CD4<500***  ***Censoring if CD4>500*** | |
| --- | --- | --- | --- | --- | --- | --- | --- |
|  |  | ***P*** | ***HR*** | ***P*** | ***HR*** | ***P*** | ***HR*** |
| rs2395029 | 4.8% | 2.7E-09 | 0.40 | 1.6E-10 | 0.45 | **1.2E-11** | **0.39** |
| rs9264942 | 41.2% | 2.0E-09 | 0.69 | 6.4E-10 | 0.72 | **7.4E-12** | **0.68** |
| rs9261174 | 14.0% | 2.2E-05 | 0.68 | 5.0E-08 | 0.66 | **3.8E-08** | **0.65** |
| CCR5 d32 | 9.9% | 1.7E-05 | 0.63 | 4.9E-06 | 0.64 | **2.6E-06** | **0.62** |

Potent antiretroviral treatment (cART) start was considered either as a censoring event (i.e. all patients starting treatment are considered non-progressors until that date) or a progression event (i.e. all patients starting treatment are considered progressors); in the last column, showing the strongest association signals, we split the patients starting cART between non-progressors and progressors on the basis of the last CD4 T cell count measured before treatment.

P-values for association and Hazard Ratio (HR) are represented for the different progression outcomes. All models include gender, age and the significant Eigenstrat axes as covariates.

MAF: minor allele frequency
