# Supplementary material for: Common Genetic Variation and the Control of HIV-1 in Humans
Source: PLoS Genet. 2009 Dec 24;5(12):e1000791. doi: 10.1371/journal.pgen.1000791 (PMC2791220; doi:10.1371/journal.pgen.1000791)
Supplement: Table S9 — Top 500 SNPs in the setpoint analysis. (0.74 MB DOC) [file pgen.1000791.s013.doc]

**Table S9**: top 500 SNPs in the setpoint analysis

| SNP | rank | P value | chromosome | coordinate | type | gene |
| --- | --- | --- | --- | --- | --- | --- |
| rs2395029 | 1 | 4.48E-35 | 6 | 31431780 | WITHIN_NON_CODING_GENE | HCP5 |
| rs9264942 | 2 | 5.85E-32 | 6 | 31274380 | UPSTREAM | HLA-C |
| rs10484554 | 3 | 8.39E-18 | 6 | 31274555 | UPSTREAM | HLA-C |
| rs2249742 | 4 | 1.56E-17 | 6 | 31240721 | UPSTREAM | HLA-C |
| rs13191519 | 5 | 1.57E-17 | 6 | 31265752 | UPSTREAM | HLA-C |
| rs13207315 | 6 | 7.06E-17 | 6 | 31241127 | UPSTREAM | HLA-C |
| rs13210132 | 7 | 1.99E-16 | 6 | 31001143 | WITHIN_NON_CODING_GENE | AL669830.2 |
| rs2395471 | 8 | 3.01E-16 | 6 | 31240692 | UPSTREAM | HLA-C |
| rs2248462 | 9 | 5.12E-15 | 6 | 31446796 | DOWNSTREAM | HCP5 |
| rs2516513 | 10 | 8.46E-15 | 6 | 31447588 | DOWNSTREAM | HCP5 |
| rs9468932 | 11 | 1.07E-14 | 6 | 31264823 | INTERGENIC |  |
| rs4418214 | 12 | 1.19E-14 | 6 | 31391401 | WITHIN_NON_CODING_GENE | N/A |
| rs2516509 | 13 | 1.35E-14 | 6 | 31449994 | DOWNSTREAM | HCP5 |
| rs9378200 | 14 | 1.88E-14 | 6 | 31572927 | INTERGENIC |  |
| rs3093662 | 15 | 3.42E-14 | 6 | 31544189 | INTRONIC | TNF |
| rs9266409 | 16 | 4.86E-14 | 6 | 31336568 | UPSTREAM | AL671883.4 |
| rs9368699 | 17 | 5.00E-14 | 6 | 31802541 | 5PRIME_UTR | C6orf48 |
| rs7756521 | 18 | 5.55E-14 | 6 | 30848253 | UPSTREAM | DDR1 |
| rs2523619 | 19 | 1.52E-13 | 6 | 31318144 | DOWNSTREAM | HLA-B |
| rs9266395 | 20 | 1.57E-13 | 6 | 31335566 | UPSTREAM | AL671883.6 |
| rs2844480 | 21 | 1.72E-13 | 6 | 31564821 | UPSTREAM | NCR3 |
| rs9348876 | 22 | 2.07E-13 | 6 | 31575276 | DOWNSTREAM | AL662801.1 |
| rs7770216 | 23 | 4.80E-13 | 6 | 31340611 | UPSTREAM | AL671883.4 |
| rs9263715 | 24 | 5.94E-13 | 6 | 31095801 | INTRONIC | N/A |
| rs3815087 | 25 | 6.67E-13 | 6 | 31093587 | 5PRIME_UTR | N/A |
| rs9267487 | 26 | 2.30E-12 | 6 | 31511350 | INTRONIC | ATP6V1G2 |
| rs3094212 | 27 | 3.23E-12 | 6 | 31085770 | INTRONIC | CDSN |
| rs2284178 | 28 | 5.01E-12 | 6 | 31432125 | WITHIN_NON_CODING_GENE | HCP5 |
| rs3873332 | 29 | 7.45E-12 | 6 | 30895990 | DOWNSTREAM | VARS2 |
| rs2894207 | 30 | 7.66E-12 | 6 | 31263751 | INTERGENIC |  |
| rs12697941 | 31 | 8.34E-12 | 6 | 30904714 | UPSTREAM | DPCR1 |
| rs3093661 | 32 | 1.33E-11 | 6 | 31543758 | INTRONIC | TNF |
| rs3093668 | 33 | 1.33E-11 | 6 | 31546495 | DOWNSTREAM | TNF |
| rs28732144 | 34 | 2.77E-11 | 6 | 31556205 | INTRONIC | LST1 |
| rs17207190 | 35 | 2.79E-11 | 6 | 31569520 | INTERGENIC |  |
| rs3873334 | 36 | 2.82E-11 | 6 | 30896147 | DOWNSTREAM | VARS2 |
| rs1051794 | 37 | 3.55E-11 | 6 | 31379109 | NON_SYNONYMOUS_CODING | MICA |
| rs6457374 | 38 | 4.38E-11 | 6 | 31272261 | UPSTREAM | HLA-C |
| rs13437082 | 39 | 4.89E-11 | 6 | 31354560 | UPSTREAM | XXbac-BPG248L24.8 |
| rs4711269 | 40 | 6.00E-11 | 6 | 31354819 | UPSTREAM | XXbac-BPG248L24.8 |
| rs2247056 | 41 | 9.57E-11 | 6 | 31265490 | INTERGENIC |  |
| rs2844511 | 42 | 1.07E-10 | 6 | 31389784 | WITHIN_NON_CODING_GENE | N/A |
| rs2596531 | 43 | 1.22E-10 | 6 | 31387557 | WITHIN_NON_CODING_GENE | MICA |
| rs4711268 | 44 | 1.30E-10 | 6 | 31354504 | UPSTREAM | XXbac-BPG248L24.8 |
| rs7772549 | 45 | 1.59E-10 | 6 | 31407643 | WITHIN_NON_CODING_GENE | AL645933.3 |
| rs3131003 | 46 | 1.80E-10 | 6 | 31093482 | 5PRIME_UTR | N/A |
| rs9295928 | 47 | 4.28E-10 | 6 | 30823630 | INTERGENIC |  |
| rs2844513 | 48 | 5.09E-10 | 6 | 31388214 | WITHIN_NON_CODING_GENE | MICA |
| rs12212594 | 49 | 5.30E-10 | 6 | 31300819 | INTERGENIC |  |
| rs4713380 | 50 | 6.45E-10 | 6 | 30785273 | WITHIN_NON_CODING_GENE | C6orf214 |
| rs12198173 | 51 | 7.26E-10 | 6 | 32026808 | INTRONIC | TNXB |
| rs3823418 | 52 | 7.46E-10 | 6 | 31100942 | INTRONIC | PSORS1C2 |
| rs4713385 | 53 | 8.96E-10 | 6 | 30787593 | WITHIN_NON_CODING_GENE | N/A |
| rs13198118 | 54 | 1.43E-09 | 6 | 30770732 | WITHIN_NON_CODING_GENE | C6orf214 |
| rs13199524 | 55 | 1.96E-09 | 6 | 32066765 | INTRONIC | TNXB |
| rs9469003 | 56 | 1.99E-09 | 6 | 31407828 | WITHIN_NON_CODING_GENE | AL645933.3 |
| rs3093553 | 57 | 2.30E-09 | 6 | 31549556 | INTRONIC | LTB |
| rs241452 | 58 | 2.97E-09 | 6 | 32796346 | 3PRIME_UTR | TAP2 |
| rs4959079 | 59 | 3.72E-09 | 6 | 31488879 | UPSTREAM | AL663061.2 |
| rs241447 | 60 | 4.35E-09 | 6 | 32796751 | NON_SYNONYMOUS_CODING | TAP2 |
| rs3871466 | 61 | 4.71E-09 | 6 | 30983683 | INTERGENIC |  |
| rs10947207 | 62 | 4.71E-09 | 6 | 31361485 | INTERGENIC |  |
| rs9501106 | 63 | 4.84E-09 | 6 | 31388109 | WITHIN_NON_CODING_GENE | MICA |
| rs241453 | 64 | 5.59E-09 | 6 | 32796226 | 3PRIME_UTR | TAP2 |
| rs2844509 | 65 | 6.40E-09 | 6 | 31510924 | INTRONIC | ATP6V1G2 |
| rs2523554 | 66 | 6.48E-09 | 6 | 31331829 | DOWNSTREAM | AL671883.6 |
| rs13216197 | 67 | 6.85E-09 | 6 | 31271018 | DOWNSTREAM | XXbac-BPG248L24.1 |
| rs241440 | 68 | 7.80E-09 | 6 | 32797361 | INTRONIC | TAP2 |
| rs8192591 | 69 | 9.02E-09 | 6 | 32185796 | NON_SYNONYMOUS_CODING | NOTCH4 |
| rs4151664 | 70 | 1.10E-08 | 6 | 31920873 | INTRONIC | RDBP |
| rs3094205 | 71 | 1.31E-08 | 6 | 31091862 | INTRONIC | CDSN |
| rs6906662 | 72 | 1.32E-08 | 6 | 32266506 | INTRONIC | N/A |
| rs3094204 | 73 | 1.34E-08 | 6 | 31091992 | INTRONIC | CDSN |
| rs9391701 | 74 | 1.37E-08 | 6 | 30983263 | INTERGENIC |  |
| rs2857106 | 75 | 1.87E-08 | 6 | 32787570 | INTRONIC | TAP2 |
| rs3130542 | 76 | 2.05E-08 | 6 | 31232111 | DOWNSTREAM | HLA-C |
| rs1062470 | 77 | 2.13E-08 | 6 | 31084435 | SYNONYMOUS_CODING | CDSN |
| rs6932730 | 78 | 2.20E-08 | 6 | 31354182 | UPSTREAM | XXbac-BPG248L24.8 |
| rs2219893 | 79 | 2.24E-08 | 6 | 32769663 | INTERGENIC |  |
| rs2524123 | 80 | 3.06E-08 | 6 | 31265314 | INTERGENIC |  |
| rs6933050 | 81 | 3.74E-08 | 6 | 31343632 | UPSTREAM | AL671883.3 |
| rs2071474 | 82 | 4.02E-08 | 6 | 32782582 | INTRONIC | HLA-DOB |
| rs241448 | 83 | 4.04E-08 | 6 | 32796685 | STOP_LOST;SPLICE_SITE | TAP2 |
| rs2071472 | 84 | 4.10E-08 | 6 | 32784620 | INTRONIC | HLA-DOB |
| rs2523535 | 85 | 4.49E-08 | 6 | 31336250 | UPSTREAM | AL671883.6 |
| rs2156875 | 86 | 4.75E-08 | 6 | 31317347 | DOWNSTREAM | HLA-B |
| rs3130473 | 87 | 5.00E-08 | 6 | 31199208 | INTERGENIC |  |
| rs12153855 | 88 | 6.35E-08 | 6 | 32074804 | INTRONIC | TNXB |
| rs2856997 | 89 | 6.59E-08 | 6 | 32781776 | INTRONIC | TAP2 |
| rs2071473 | 90 | 8.36E-08 | 6 | 32782605 | INTRONIC | HLA-DOB |
| rs3095320 | 91 | 9.87E-08 | 6 | 31087934 | INTRONIC | CDSN |
| rs2523608 | 92 | 9.95E-08 | 6 | 31322559 | INTRONIC | HLA-B |
| rs2523674 | 93 | 1.06E-07 | 6 | 31436789 | WITHIN_NON_CODING_GENE | HCP5 |
| rs3130981 | 94 | 1.09E-07 | 6 | 31083813 | NON_SYNONYMOUS_CODING | CDSN |
| rs9266845 | 95 | 1.11E-07 | 6 | 31384792 | WITHIN_NON_CODING_GENE | MICA |
| rs9266825 | 96 | 1.37E-07 | 6 | 31382882 | NON_SYNONYMOUS_CODING | MICA |
| rs2523467 | 97 | 1.43E-07 | 6 | 31362930 | UPSTREAM | MICA |
| rs12191877 | 98 | 1.57E-07 | 6 | 31252925 | DOWNSTREAM | AL671883.7 |
| rs2844529 | 99 | 1.60E-07 | 6 | 31353593 | UPSTREAM | XXbac-BPG248L24.8 |
| rs2248372 | 100 | 1.75E-07 | 6 | 31446466 | DOWNSTREAM | HCP5 |
| rs2428486 | 101 | 1.99E-07 | 6 | 31354104 | UPSTREAM | XXbac-BPG248L24.8 |
| rs2621373 | 102 | 2.49E-07 | 6 | 32765130 | INTERGENIC |  |
| rs9295924 | 103 | 2.66E-07 | 6 | 30782361 | WITHIN_NON_CODING_GENE | C6orf214 |
| rs2534657 | 104 | 2.86E-07 | 6 | 31472459 | INTRONIC | MICB |
| rs9261290 | 105 | 3.26E-07 | 6 | 30038647 | SYNONYMOUS_CODING | RNF39 |
| rs719654 | 106 | 3.31E-07 | 6 | 32752139 | INTERGENIC |  |
| rs2516448 | 107 | 3.34E-07 | 6 | 31390410 | WITHIN_NON_CODING_GENE | N/A |
| rs9366778 | 108 | 4.42E-07 | 6 | 31269173 | INTERGENIC |  |
| rs720465 | 109 | 4.53E-07 | 6 | 31125777 | SPLICE_SITE;5PRIME_UTR | CCHCR1 |
| rs9378109 | 110 | 4.58E-07 | 6 | 30774474 | WITHIN_NON_CODING_GENE | N/A |
| rs8321 | 111 | 4.72E-07 | 6 | 30032522 | REGULATORY_REGION;3PRIME_UTR | ZNRD1 |
| rs2596542 | 112 | 4.78E-07 | 6 | 31366595 | REGULATORY_REGION;UPSTREAM | MICA |
| rs2256175 | 113 | 4.94E-07 | 6 | 31380449 | INTRONIC | MICA |
| rs3828917 | 114 | 5.11E-07 | 6 | 31465917 | 5PRIME_UTR | MICB |
| rs1894406 | 115 | 5.21E-07 | 6 | 32787056 | INTRONIC | HLA-DOB |
| rs3130380 | 116 | 5.40E-07 | 6 | 30279130 | WITHIN_NON_CODING_GENE | N/A |
| rs3130424 | 117 | 6.60E-07 | 6 | 31218239 | INTERGENIC |  |
| rs3134899 | 118 | 6.76E-07 | 6 | 31473286 | INTRONIC | MICB |
| rs9263870 | 119 | 7.14E-07 | 6 | 31170514 | WITHIN_NON_CODING_GENE | HCG27 |
| rs3094187 | 120 | 8.22E-07 | 6 | 31126944 | 5PRIME_UTR | TCF19 |
| rs2516400 | 121 | 8.75E-07 | 6 | 31481105 | DOWNSTREAM | MICB |
| rs2534678 | 122 | 9.78E-07 | 6 | 31463963 | UPSTREAM | AL663061.4 |
| rs12185555 | 123 | 9.99E-07 | 2 | 227559317 | INTERGENIC |  |
| rs13201769 | 124 | 1.01E-06 | 6 | 30756066 | WITHIN_NON_CODING_GENE | AL662797.4 |
| rs3130544 | 125 | 1.06E-06 | 6 | 31058340 | INTERGENIC |  |
| rs1052248 | 126 | 1.13E-06 | 6 | 31556581 | 3PRIME_UTR | LST1 |
| rs7356880 | 127 | 1.16E-06 | 6 | 32401327 | INTERGENIC |  |
| rs1265159 | 128 | 1.24E-06 | 6 | 31140047 | REGULATORY_REGION;UPSTREAM | PSORS1C3 |
| rs3869129 | 129 | 1.31E-06 | 6 | 31410649 | WITHIN_NON_CODING_GENE | AL645933.3 |
| rs9264508 | 130 | 1.33E-06 | 6 | 31233214 | DOWNSTREAM | HLA-C |
| rs12190030 | 131 | 1.33E-06 | 6 | 30921364 | 3PRIME_UTR | DPCR1 |
| rs12557137 | 132 | 1.41E-06 | X | 31348807 | INTRONIC | N/A |
| rs28732100 | 133 | 1.53E-06 | 6 | 31104593 | INTRONIC | PSORS1C1 |
| rs185819 | 134 | 1.83E-06 | 6 | 32050067 | SYNONYMOUS_CODING | TNXA |
| rs9264536 | 135 | 1.84E-06 | 6 | 31234541 | DOWNSTREAM | HLA-C |
| rs1235162 | 136 | 1.86E-06 | 6 | 29537224 | INTRONIC | OR2H5P |
| rs2857204 | 137 | 1.98E-06 | 6 | 32744347 | INTERGENIC |  |
| rs13195441 | 138 | 2.07E-06 | 6 | 32743298 | INTERGENIC |  |
| rs10484399 | 139 | 2.20E-06 | 6 | 27534528 | INTERGENIC |  |
| rs2858332 | 140 | 2.27E-06 | 6 | 32681161 | UPSTREAM | AL662789.1 |
| rs17749927 | 141 | 2.41E-06 | 6 | 27669976 | INTERGENIC |  |
| rs3132610 | 142 | 2.47E-06 | 6 | 30544401 | INTRONIC | ABCF1 |
| rs9378127 | 143 | 2.55E-06 | 6 | 32922459 | INTRONIC | HLA-DMA |
| rs3095329 | 144 | 2.65E-06 | 6 | 30693816 | DOWNSTREAM | TUBB |
| rs12111032 | 145 | 2.72E-06 | 6 | 31242191 | UPSTREAM | USP8P |
| rs3130685 | 146 | 2.86E-06 | 6 | 31206206 | INTERGENIC |  |
| rs9677779 | 147 | 2.87E-06 | 2 | 71903036 | INTRONIC | N/A |
| rs11800642 | 148 | 3.23E-06 | 1 | 212909744 | INTRONIC | NSL1 |
| rs9262143 | 149 | 3.72E-06 | 6 | 30652781 | NON_SYNONYMOUS_CODING | KIAA1949 |
| rs3130055 | 150 | 3.74E-06 | 6 | 31497399 | INTRONIC | MCCD1 |
| rs9263871 | 151 | 3.99E-06 | 6 | 31170528 | WITHIN_NON_CODING_GENE | HCG27 |
| rs2596503 | 152 | 4.06E-06 | 6 | 31320810 | UPSTREAM | HLA-B |
| rs3749971 | 153 | 4.10E-06 | 6 | 29342775 | SYNONYMOUS_CODING | OR12D3 |
| rs2516424 | 154 | 4.34E-06 | 6 | 31448315 | DOWNSTREAM | HCP5 |
| rs6931921 | 155 | 4.63E-06 | 6 | 31095003 | INTRONIC | N/A |
| rs2229094 | 156 | 5.01E-06 | 6 | 31540556 | NON_SYNONYMOUS_CODING | LTA |
| rs13194781 | 157 | 5.05E-06 | 6 | 27815639 | INTERGENIC |  |
| rs9264602 | 158 | 5.38E-06 | 6 | -9 | N/A |  |
| rs3735118 | 159 | 5.68E-06 | 7 | 2944317 | DOWNSTREAM | CARD11 |
| rs2394950 | 160 | 5.79E-06 | 6 | 31230701 | INTERGENIC |  |
| rs13204672 | 161 | 5.89E-06 | 6 | 32582796 | UPSTREAM | HLA-DRB1 |
| rs2395488 | 162 | 5.96E-06 | 6 | 31445909 | DOWNSTREAM | HCP5 |
| rs2248617 | 163 | 6.09E-06 | 6 | 31448533 | DOWNSTREAM | HCP5 |
| rs2071469 | 164 | 6.17E-06 | 6 | 32784783 | 5PRIME_UTR | HLA-DOB |
| rs12196597 | 165 | 6.21E-06 | 6 | 32941214 | INTRONIC | BRD2 |
| rs4711249 | 166 | 6.39E-06 | 6 | 30908266 | UPSTREAM | DPCR1 |
| rs9501035 | 167 | 6.39E-06 | 6 | 30912414 | INTRONIC | DPCR1 |
| rs3131064 | 168 | 7.21E-06 | 6 | 30763893 | DOWNSTREAM | C6orf214 |
| rs3828914 | 169 | 7.57E-06 | 6 | 31465819 | UPSTREAM | MICB |
| rs3909130 | 170 | 7.82E-06 | 6 | 30874165 | UPSTREAM | GTF2H4 |
| rs6702101 | 171 | 7.86E-06 | 1 | 212913590 | INTRONIC | NSL1 |
| rs3213644 | 172 | 8.38E-06 | 6 | 30861225 | INTRONIC | DDR1 |
| rs2284175 | 173 | 8.56E-06 | 6 | 30875145 | UPSTREAM | GTF2H4 |
| rs2074510 | 174 | 8.56E-06 | 6 | 30876034 | 5PRIME_UTR | VARS2 |
| rs1052693 | 175 | 8.56E-06 | 6 | 30876152 | 5PRIME_UTR | VARS2 |
| rs916920 | 176 | 8.56E-06 | 6 | 30877202 | INTRONIC | VARS2 |
| rs3218815 | 177 | 8.56E-06 | 6 | 30878769 | INTRONIC | GTF2H4 |
| rs2074512 | 178 | 8.56E-06 | 6 | 30878919 | INTRONIC | GTF2H4 |
| rs28383344 | 179 | 8.63E-06 | 6 | 32605067 | INTRONIC | HLA-DQA1 |
| rs6932590 | 180 | 8.73E-06 | 6 | 27248931 | UPSTREAM | AL021808.1 |
| rs2746150 | 181 | 8.95E-06 | 6 | 29442701 | DOWNSTREAM | MAS1LP |
| rs6540762 | 182 | 8.97E-06 | 1 | 212925857 | INTRONIC | N/A |
| rs12404885 | 183 | 8.97E-06 | 1 | 212927167 | INTRONIC | N/A |
| rs11120014 | 184 | 8.97E-06 | 1 | 212933545 | INTRONIC | NSL1 |
| rs11808846 | 185 | 8.97E-06 | 1 | 212943581 | INTRONIC | N/A |
| rs3738798 | 186 | 8.97E-06 | 1 | 212965469 | INTRONIC | TATDN3 |
| rs7904001 | 187 | 9.07E-06 | 10 | 66762723 | INTERGENIC |  |
| rs2284176 | 188 | 9.22E-06 | 6 | 30875622 | UPSTREAM | GTF2H4 |
| rs2516511 | 189 | 9.23E-06 | 6 | 31448625 | DOWNSTREAM | HCP5 |
| rs3094054 | 190 | 9.27E-06 | 6 | 30333505 | UPSTREAM | AL662795.3 |
| rs16959323 | 191 | 9.40E-06 | 17 | 10006097 | INTRONIC | N/A |
| rs3093978 | 192 | 9.46E-06 | 6 | 31498497 | INTRONIC | BAT1 |
| rs2894055 | 193 | 9.75E-06 | 6 | 30868628 | DOWNSTREAM | DDR1 |
| rs3778639 | 194 | 9.76E-06 | 6 | 31093776 | INTRONIC | N/A |
| rs9468843 | 195 | 9.77E-06 | 6 | 30867958 | DOWNSTREAM | DDR1 |
| rs28732157 | 196 | 9.78E-06 | 6 | 31628216 | 5PRIME_UTR | AL662899.2 |
| rs7751505 | 197 | 9.92E-06 | 6 | 31360255 | INTERGENIC |  |
| rs7751725 | 198 | 1.00E-05 | 6 | 31360433 | INTERGENIC |  |
| rs6905949 | 199 | 1.02E-05 | 6 | 30140525 | DOWNSTREAM | TRIM15 |
| rs2516393 | 200 | 1.02E-05 | 6 | 31506744 | INTRONIC | BAT1 |
| rs3093983 | 201 | 1.04E-05 | 6 | 31496925 | SYNONYMOUS_CODING | MCCD1 |
| rs2523705 | 202 | 1.05E-05 | 6 | 31451680 | INTERGENIC |  |
| rs2904600 | 203 | 1.06E-05 | 6 | 31453113 | INTERGENIC |  |
| rs3869086 | 204 | 1.08E-05 | 6 | 30870168 | DOWNSTREAM | DDR1 |
| rs4713462 | 205 | 1.10E-05 | 6 | 31347816 | UPSTREAM | AL671883.2 |
| rs7743661 | 206 | 1.15E-05 | 6 | 30858254 | INTRONIC | DDR1 |
| rs9257809 | 207 | 1.15E-05 | 6 | 29356331 | INTRONIC | N/A |
| rs3130350 | 208 | 1.15E-05 | 6 | 30327839 | UPSTREAM | AL662795.2 |
| rs6917363 | 209 | 1.16E-05 | 6 | 31247169 | UPSTREAM | USP8P |
| rs12128410 | 210 | 1.17E-05 | 1 | 212972599 | INTRONIC | TATDN3 |
| rs9501032 | 211 | 1.18E-05 | 6 | 30850191 | INTRONIC | DDR1 |
| rs3094127 | 212 | 1.19E-05 | 6 | 30697447 | INTRONIC | FLOT1 |
| rs9295930 | 213 | 1.19E-05 | 6 | 30849822 | INTRONIC | DDR1 |
| rs9266440 | 214 | 1.19E-05 | 6 | 31337815 | UPSTREAM | AL671883.4 |
| rs2239518 | 215 | 1.21E-05 | 6 | 30865725 | INTRONIC | DDR1 |
| rs2286655 | 216 | 1.21E-05 | 6 | 30899746 | INTRONIC | SFTA2 |
| rs9461638 | 217 | 1.22E-05 | 6 | 30851305 | INTRONIC | DDR1 |
| rs6901464 | 218 | 1.22E-05 | 6 | 30854090 | INTRONIC | DDR1 |
| rs4618569 | 219 | 1.22E-05 | 6 | 30855251 | INTRONIC | DDR1 |
| rs6924600 | 220 | 1.22E-05 | 6 | 30857542 | INTRONIC | DDR1 |
| rs2239517 | 221 | 1.22E-05 | 6 | 30865115 | INTRONIC | DDR1 |
| rs1049628 | 222 | 1.22E-05 | 6 | 30867106 | 3PRIME_UTR | DDR1 |
| rs8408 | 223 | 1.22E-05 | 6 | 30867666 | 3PRIME_UTR | DDR1 |
| rs9295931 | 224 | 1.22E-05 | 6 | 30869714 | DOWNSTREAM | DDR1 |
| rs9468846 | 225 | 1.22E-05 | 6 | 30870763 | DOWNSTREAM | DDR1 |
| rs12206075 | 226 | 1.22E-05 | 6 | 30871000 | UPSTREAM | DDR1 |
| rs13215409 | 227 | 1.22E-05 | 6 | 30871619 | UPSTREAM | DDR1 |
| rs3095250 | 228 | 1.22E-05 | 6 | 31208340 | INTERGENIC |  |
| rs7515115 | 229 | 1.23E-05 | 1 | 212986554 | INTRONIC | TATDN3 |
| rs2074511 | 230 | 1.24E-05 | 6 | 30889389 | SYNONYMOUS_CODING | VARS2 |
| rs3132584 | 231 | 1.25E-05 | 6 | 30688427 | INTRONIC | TUBB |
| rs5954635 | 232 | 1.31E-05 | X | 141450842 | INTERGENIC |  |
| rs2240803 | 233 | 1.37E-05 | 6 | 30920957 | 3PRIME_UTR | DPCR1 |
| rs2286656 | 234 | 1.38E-05 | 6 | 30899571 | SPLICE_SITE;SYNONYMOUS_CODING | SFTA2 |
| rs3099844 | 235 | 1.41E-05 | 6 | 31448976 | DOWNSTREAM | HCP5 |
| rs2229933 | 236 | 1.42E-05 | 6 | 30857072 | SYNONYMOUS_CODING | DDR1 |
| rs3130688 | 237 | 1.43E-05 | 6 | 31210216 | INTERGENIC |  |
| rs10073652 | 238 | 1.60E-05 | 5 | 12786980 | INTERGENIC |  |
| rs6640729 | 239 | 1.63E-05 | X | 11346716 | INTRONIC | N/A |
| rs2596530 | 240 | 1.64E-05 | 6 | 31387373 | WITHIN_NON_CODING_GENE | MICA |
| rs2280801 | 241 | 1.71E-05 | 6 | 31592064 | NON_SYNONYMOUS_CODING | BAT2 |
| rs3218355 | 242 | 1.74E-05 | 22 | 37532954 | INTRONIC | IL2RB |
| rs1265115 | 243 | 1.74E-05 | 6 | 31117075 | INTRONIC | CCHCR1 |
| rs630379 | 244 | 1.78E-05 | 6 | 31922254 | INTRONIC | RDBP |
| rs9468842 | 245 | 1.81E-05 | 6 | 30852747 | INTRONIC | DDR1 |
| rs486299 | 246 | 1.84E-05 | 13 | 72946009 | INTERGENIC |  |
| rs11753654 | 247 | 1.87E-05 | 6 | 29782621 | UPSTREAM | MICG |
| rs7521639 | 248 | 1.90E-05 | 1 | 227223736 | INTRONIC | CDC42BPA |
| rs3134792 | 249 | 1.90E-05 | 6 | 31312326 | INTERGENIC |  |
| rs9404974 | 250 | 1.94E-05 | 6 | 30776483 | WITHIN_NON_CODING_GENE | C6orf214 |
| rs9380198 | 251 | 1.94E-05 | 6 | 30785886 | WITHIN_NON_CODING_GENE | N/A |
| rs4713382 | 252 | 1.94E-05 | 6 | 30787175 | WITHIN_NON_CODING_GENE | N/A |
| rs4713383 | 253 | 1.94E-05 | 6 | 30787241 | WITHIN_NON_CODING_GENE | N/A |
| rs4713389 | 254 | 1.94E-05 | 6 | 30790604 | WITHIN_NON_CODING_GENE | N/A |
| rs6901761 | 255 | 1.94E-05 | 6 | 30790798 | WITHIN_NON_CODING_GENE | N/A |
| rs4947289 | 256 | 1.94E-05 | 6 | 30791409 | WITHIN_NON_CODING_GENE | N/A |
| rs7751869 | 257 | 1.94E-05 | 6 | 30793314 | WITHIN_NON_CODING_GENE | N/A |
| rs4947290 | 258 | 1.94E-05 | 6 | 30794405 | WITHIN_NON_CODING_GENE | C6orf214 |
| rs4765914 | 259 | 1.99E-05 | 12 | 2420377 | INTRONIC | N/A |
| rs7158298 | 260 | 2.01E-05 | 14 | 96760563 | INTRONIC | ATG2B |
| rs1233397 | 261 | 2.03E-05 | 6 | 29545715 | INTRONIC | TMEM183AP1 |
| rs6441977 | 262 | 2.07E-05 | 3 | 46450072 | NON_SYNONYMOUS_CODING | CCRL2 |
| rs4905480 | 263 | 2.22E-05 | 14 | 96759853 | INTRONIC | ATG2B |
| rs2442719 | 264 | 2.28E-05 | 6 | 31320538 | UPSTREAM | HLA-B |
| rs7652037 | 265 | 2.34E-05 | 3 | 46423063 | WITHIN_NON_CODING_GENE | N/A |
| rs13197574 | 266 | 2.34E-05 | 6 | 28060239 | UPSTREAM | ZSCAN12L1 |
| rs34214527 | 267 | 2.47E-05 | 6 | 32014456 | INTRONIC | TNXB |
| rs34241101 | 268 | 2.49E-05 | 6 | 31936057 | INTRONIC | SKIV2L |
| rs7810813 | 269 | 2.50E-05 | 7 | 2943818 | DOWNSTREAM | CARD11 |
| rs2858331 | 270 | 2.52E-05 | 6 | 32681277 | UPSTREAM | AL662789.1 |
| rs2517403 | 271 | 2.53E-05 | 6 | 31067009 | INTERGENIC |  |
| rs28732150 | 272 | 2.56E-05 | 6 | 31583224 | INTRONIC | AIF1 |
| rs10863997 | 273 | 2.62E-05 | 1 | 212947520 | INTRONIC | N/A |
| rs3095340 | 274 | 2.67E-05 | 6 | 30726939 | INTERGENIC |  |
| rs12033847 | 275 | 2.68E-05 | 1 | 176027978 | INTRONIC | N/A |
| rs9394023 | 276 | 2.71E-05 | 6 | 30947042 | UPSTREAM | MUC21 |
| rs2844635 | 277 | 2.75E-05 | 6 | 31075481 | DOWNSTREAM | C6orf15 |
| rs8101017 | 278 | 2.77E-05 | 19 | 41723887 | UPSTREAM | AXL |
| rs2524074 | 279 | 2.79E-05 | 6 | 31244021 | WITHIN_NON_CODING_GENE | USP8P |
| rs3130467 | 280 | 2.84E-05 | 6 | 31187075 | INTERGENIC |  |
| rs981395 | 281 | 2.91E-05 | 2 | 20346774 | INTERGENIC |  |
| rs4676420 | 282 | 2.95E-05 | 2 | 241527673 | INTRONIC | CAPN10 |
| rs1096699 | 283 | 2.95E-05 | 6 | 43528441 | INTRONIC | XPO5 |
| rs1131904 | 284 | 3.03E-05 | 6 | 31383071 | 3PRIME_UTR | MICA |
| rs9266399 | 285 | 3.04E-05 | 6 | 31335806 | UPSTREAM | AL671883.6 |
| rs2905722 | 286 | 3.10E-05 | 6 | 31449327 | DOWNSTREAM | HCP5 |
| rs2516415 | 287 | 3.14E-05 | 6 | 31459742 | DOWNSTREAM | AL663061.4 |
| rs1064627 | 288 | 3.17E-05 | 6 | 30698541 | INTRONIC | FLOT1 |
| rs11713226 | 289 | 3.18E-05 | 3 | 140970556 | INTRONIC | N/A |
| rs28724903 | 290 | 3.21E-05 | 6 | 32924061 | INTRONIC | HLA-DMA |
| rs3131018 | 291 | 3.21E-05 | 6 | 31143582 | WITHIN_NON_CODING_GENE | PSORS1C3 |
| rs1233579 | 292 | 3.29E-05 | 6 | 28712663 | INTERGENIC |  |
| rs1532951 | 293 | 3.40E-05 | 1 | 212978197 | INTRONIC | TATDN3 |
| rs1233391 | 294 | 3.51E-05 | 6 | 29550618 | INTRONIC | AL645936.2 |
| rs1063478 | 295 | 3.54E-05 | 6 | 32917544 | NON_SYNONYMOUS_CODING | HLA-DMA |
| rs3117143 | 296 | 3.56E-05 | 6 | 29031142 | INTERGENIC |  |
| rs7750641 | 297 | 3.56E-05 | 6 | 31129310 | NON_SYNONYMOUS_CODING | TCF19 |
| rs9595729 | 298 | 3.58E-05 | 13 | 48092853 | INTERGENIC |  |
| rs2734583 | 299 | 3.66E-05 | 6 | 31505480 | INTRONIC | BAT1 |
| rs2256919 | 300 | 3.71E-05 | 6 | 29940750 | UPSTREAM | MICD |
| rs2869525 | 301 | 3.72E-05 | X | 141451485 | INTERGENIC |  |
| rs17218614 | 302 | 3.75E-05 | 9 | 109177377 | WITHIN_NON_CODING_GENE | N/A |
| rs12211410 | 303 | 3.75E-05 | 6 | 32049423 | NON_SYNONYMOUS_CODING | TNXA |
| rs1265099 | 304 | 3.77E-05 | 6 | 31105413 | 3PRIME_UTR | PSORS1C2 |
| rs3918149 | 305 | 3.87E-05 | 6 | 32936373 | INTRONIC | HLA-DMA |
| rs1552553 | 306 | 3.90E-05 | 18 | 64444457 | INTERGENIC |  |
| rs153239 | 307 | 3.90E-05 | 5 | 80425860 | INTRONIC | N/A |
| rs28724890 | 308 | 3.90E-05 | 6 | 32741844 | INTERGENIC |  |
| rs5009448 | 309 | 3.92E-05 | 6 | 29940488 | UPSTREAM | MICD |
| rs2059663 | 310 | 3.96E-05 | 18 | 64527019 | INTERGENIC |  |
| rs4841367 | 311 | 3.98E-05 | 8 | 10388826 | SYNONYMOUS_CODING | UNQ9391 |
| rs4416711 | 312 | 4.00E-05 | 6 | 31221039 | INTERGENIC |  |
| rs12660883 | 313 | 4.09E-05 | 6 | 30764420 | DOWNSTREAM | C6orf214 |
| rs2295663 | 314 | 4.10E-05 | 6 | 31669295 | INTRONIC | BAT5 |
| rs12700978 | 315 | 4.12E-05 | 7 | 29816649 | INTERGENIC |  |
| rs17094001 | 316 | 4.22E-05 | 14 | 96759056 | INTRONIC | ATG2B |
| rs3819299 | 317 | 4.29E-05 | 6 | 31322367 | INTRONIC | HLA-B |
| rs3130453 | 318 | 4.39E-05 | 6 | 31124849 | SYNONYMOUS_CODING | CCHCR1 |
| rs2844623 | 319 | 4.40E-05 | 6 | 31232543 | DOWNSTREAM | HLA-C |
| rs12606152 | 320 | 4.42E-05 | 18 | 64445511 | INTERGENIC |  |
| rs2858870 | 321 | 4.46E-05 | 6 | 32572251 | WITHIN_NON_CODING_GENE | N/A |
| rs3094217 | 322 | 4.48E-05 | 6 | 31083656 | 3PRIME_UTR | CDSN |
| rs1042134 | 323 | 4.48E-05 | 6 | 31083664 | 3PRIME_UTR | CDSN |
| rs1042126 | 324 | 4.48E-05 | 6 | 31084288 | SYNONYMOUS_CODING | CDSN |
| rs3130983 | 325 | 4.48E-05 | 6 | 31084792 | SYNONYMOUS_CODING | CDSN |
| rs3094214 | 326 | 4.48E-05 | 6 | 31085382 | INTRONIC | CDSN |
| rs17034 | 327 | 4.55E-05 | 6 | 32796521 | 3PRIME_UTR | TAP2 |
| rs357708 | 328 | 4.64E-05 | 2 | 180664033 | INTRONIC | ZNF385B |
| rs2394412 | 329 | 4.64E-05 | 6 | 30782235 | WITHIN_NON_CODING_GENE | C6orf214 |
| rs3130564 | 330 | 4.65E-05 | 6 | 31101674 | INTRONIC | PSORS1C2 |
| rs13194504 | 331 | 4.66E-05 | 6 | 28630691 | INTERGENIC |  |
| rs2915287 | 332 | 4.68E-05 | 3 | 87957076 | UPSTREAM | AC119034.1 |
| rs4858927 | 333 | 4.68E-05 | 3 | 88030689 | INTERGENIC |  |
| rs4858970 | 334 | 4.68E-05 | 3 | 88048652 | INTERGENIC |  |
| rs1431154 | 335 | 4.68E-05 | 3 | 88074793 | INTERGENIC |  |
| rs1548842 | 336 | 4.68E-05 | 16 | 6356187 | INTERGENIC |  |
| rs4616669 | 337 | 4.73E-05 | 3 | 88086823 | INTERGENIC |  |
| rs130072 | 338 | 4.73E-05 | 6 | 31112484 | NON_SYNONYMOUS_CODING | CCHCR1 |
| rs7011404 | 339 | 4.82E-05 | 8 | 77604704 | INTRONIC | N/A |
| rs9391734 | 340 | 4.87E-05 | 6 | 32097983 | 5PRIME_UTR | FKBPL |
| rs13211318 | 341 | 4.87E-05 | 6 | 32102680 | UPSTREAM | FKBPL |
| rs9275572 | 342 | 4.92E-05 | 6 | 32678999 | UPSTREAM | MTCO3P1 |
| rs17164981 | 343 | 4.98E-05 | 7 | 130101373 | INTERGENIC |  |
| rs13011089 | 344 | 4.98E-05 | 2 | 123828854 | WITHIN_NON_CODING_GENE | AC062020.1 |
| rs4842868 | 345 | 4.98E-05 | 15 | 85665622 | INTRONIC | N/A |
| rs4869685 | 346 | 5.20E-05 | 5 | 36659084 | INTRONIC | N/A |
| rs7251251 | 347 | 5.23E-05 | 19 | 50294150 | INTRONIC | N/A |
| rs9541976 | 348 | 5.24E-05 | 13 | 35297304 | INTERGENIC |  |
| rs2647012 | 349 | 5.29E-05 | 6 | 32664458 | INTERGENIC |  |
| rs6909321 | 350 | 5.29E-05 | 6 | 31093190 | INTRONIC | CDSN |
| rs2427622 | 351 | 5.31E-05 | 20 | 62860980 | INTRONIC | MYT1 |
| rs929138 | 352 | 5.37E-05 | 6 | 31503698 | INTRONIC | BAT1 |
| rs1983249 | 353 | 5.41E-05 | 18 | 42134447 | INTERGENIC |  |
| rs2856725 | 354 | 5.48E-05 | 6 | 32666738 | INTERGENIC |  |
| rs9264219 | 355 | 5.50E-05 | 6 | 31221914 | INTERGENIC |  |
| rs12207951 | 356 | 5.54E-05 | 6 | -9 | N/A |  |
| rs2894046 | 357 | 5.57E-05 | 6 | 30782105 | WITHIN_NON_CODING_GENE | C6orf214 |
| rs2857101 | 358 | 5.59E-05 | 6 | 32794676 | 3PRIME_UTR | TAP2 |
| rs9295938 | 359 | 5.60E-05 | 6 | 30953105 | INTRONIC | MUC21 |
| rs1419881 | 360 | 5.72E-05 | 6 | 31130593 | 3PRIME_UTR | TCF19 |
| rs9261487 | 361 | 5.74E-05 | 6 | 30109207 | INTRONIC | TRIM40 |
| rs17013364 | 362 | 5.86E-05 | 2 | 77153600 | INTRONIC | N/A |
| rs2164613 | 363 | 5.98E-05 | 1 | 217003419 | INTRONIC | N/A |
| rs1550357 | 364 | 5.99E-05 | 5 | 124477401 | INTERGENIC |  |
| rs10125010 | 365 | 6.01E-05 | 9 | 21216028 | DOWNSTREAM | IFNA16 |
| rs3820416 | 366 | 6.02E-05 | 1 | 170707675 | 3PRIME_UTR | PRRX1 |
| rs17798594 | 367 | 6.03E-05 | 5 | 63793107 | INTERGENIC |  |
| rs1076435 | 368 | 6.06E-05 | 7 | 157278764 | INTRONIC | N/A |
| rs4607472 | 369 | 6.08E-05 | 6 | 29810543 | INTERGENIC |  |
| rs1042147 | 370 | 6.13E-05 | 6 | 31083156 | 3PRIME_UTR | CDSN |
| rs2301753 | 371 | 6.22E-05 | 6 | 30039240 | NON_SYNONYMOUS_CODING | RNF39 |
| rs13214831 | 372 | 6.23E-05 | 6 | 30731505 | UPSTREAM | AL662797.4 |
| rs3132685 | 373 | 6.23E-05 | 6 | 29945949 | WITHIN_NON_CODING_GENE | HCG9 |
| rs560279 | 374 | 6.25E-05 | 11 | 122023511 | UPSTREAM | AP001359.2 |
| rs4723133 | 375 | 6.27E-05 | 7 | 32247043 | INTRONIC | N/A |
| rs1049623 | 376 | 6.30E-05 | 6 | 30864829 | SYNONYMOUS_CODING | DDR1 |
| rs17745496 | 377 | 6.32E-05 | 3 | 57902639 | NON_SYNONYMOUS_CODING | SLMAP |
| rs7331491 | 378 | 6.32E-05 | 13 | 25237504 | INTERGENIC |  |
| rs886424 | 379 | 6.39E-05 | 6 | 30782002 | WITHIN_NON_CODING_GENE | C6orf214 |
| rs2199874 | 380 | 6.45E-05 | 6 | 32769926 | INTERGENIC |  |
| rs3130712 | 381 | 6.46E-05 | 6 | 31209510 | INTERGENIC |  |
| rs1264323 | 382 | 6.49E-05 | 6 | 30855907 | INTRONIC | DDR1 |
| rs2332094 | 383 | 6.64E-05 | 14 | 70069672 | INTERGENIC |  |
| rs3117326 | 384 | 6.65E-05 | 6 | 29240378 | INTRONIC | OR12D3 |
| rs153236 | 385 | 6.65E-05 | 5 | 80427640 | INTRONIC | N/A |
| rs10954043 | 386 | 6.69E-05 | 7 | 124322409 | INTERGENIC |  |
| rs198806 | 387 | 6.70E-05 | 6 | 26133616 | INTRONIC | N/A |
| rs34045601 | 388 | 6.73E-05 | 3 | -9 | N/A |  |
| rs6904596 | 389 | 6.77E-05 | 6 | 27491299 | WITHIN_NON_CODING_GENE | AL021918.1 |
| rs1999722 | 390 | 6.78E-05 | 14 | 70156668 | INTRONIC | N/A |
| rs10076290 | 391 | 6.90E-05 | 5 | 165252035 | INTERGENIC |  |
| rs1617105 | 392 | 6.90E-05 | 6 | 43613180 | INTRONIC | RSPH9 |
| rs1265158 | 393 | 6.92E-05 | 6 | 31140741 | UPSTREAM | PSORS1C3 |
| rs7668282 | 394 | 6.98E-05 | 4 | 69962114 | INTRONIC | UGT2B7 |
| rs2523849 | 395 | 6.99E-05 | 6 | 31025051 | WITHIN_NON_CODING_GENE | AL669830.3 |
| rs9826668 | 396 | 7.02E-05 | 3 | 139271591 | INTERGENIC |  |
| rs5968783 | 397 | 7.05E-05 | X | 85349613 | INTERGENIC |  |
| rs2532934 | 398 | 7.10E-05 | 6 | 30894759 | DOWNSTREAM | VARS2 |
| rs12409048 | 399 | 7.10E-05 | 1 | 170699149 | INTRONIC | PRRX1 |
| rs9368677 | 400 | 7.12E-05 | 6 | 31272321 | DOWNSTREAM | XXbac-BPG248L24.1 |
| rs6739163 | 401 | 7.13E-05 | 2 | 3419011 | INTRONIC | N/A |
| rs4713366 | 402 | 7.25E-05 | 6 | 30756361 | WITHIN_NON_CODING_GENE | AL662797.4 |
| rs7934994 | 403 | 7.29E-05 | 11 | 118005780 | 3PRIME_UTR | SCN4B |
| rs12723353 | 404 | 7.31E-05 | 1 | 216984496 | INTRONIC | N/A |
| rs17055932 | 405 | 7.36E-05 | 13 | 38067143 | INTERGENIC |  |
| rs28895018 | 406 | 7.37E-05 | 6 | 32224378 | WITHIN_NON_CODING_GENE | AL671511.1 |
| rs4324798 | 407 | 7.43E-05 | 6 | 28776117 | INTERGENIC |  |
| rs12080623 | 408 | 7.56E-05 | 1 | 157665064 | INTRONIC | AL356276.1 |
| rs11971511 | 409 | 7.65E-05 | 7 | 3434275 | INTRONIC | N/A |
| rs2621321 | 410 | 7.66E-05 | 6 | 32789480 | INTRONIC | TAP2 |
| rs9662936 | 411 | 7.77E-05 | 1 | 235248412 | INTERGENIC |  |
| rs2361345 | 412 | 7.81E-05 | 3 | 57666571 | INTRONIC | N/A |
| rs12119861 | 413 | 7.83E-05 | 1 | 161985004 | INTRONIC | N/A |
| rs9324755 | 414 | 7.86E-05 | 5 | 153064097 | INTRONIC | N/A |
| rs10035262 | 415 | 7.86E-05 | 5 | 153065746 | INTRONIC | N/A |
| rs9257802 | 416 | 7.88E-05 | 6 | 29343355 | INTRONIC | OR12D3 |
| rs8009171 | 417 | 7.88E-05 | 14 | 81820060 | INTRONIC | N/A |
| rs5978434 | 418 | 7.92E-05 | X | 11433808 | INTRONIC | N/A |
| rs241437 | 419 | 8.01E-05 | 6 | 32797684 | INTRONIC | TAP2 |
| rs7460570 | 420 | 8.16E-05 | 8 | 139261705 | INTRONIC | N/A |
| rs3131093 | 421 | 8.19E-05 | 6 | 28837437 | INTERGENIC |  |
| rs17003021 | 422 | 8.25E-05 | 4 | 79098129 | INTRONIC | N/A |
| rs8233 | 423 | 8.30E-05 | 6 | 30692965 | 3PRIME_UTR | TUBB |
| rs2073724 | 424 | 8.30E-05 | 6 | 31129707 | NON_SYNONYMOUS_CODING | TCF19 |
| rs11741331 | 425 | 8.31E-05 | 5 | 124488902 | INTERGENIC |  |
| rs2596472 | 426 | 8.37E-05 | 6 | 31428967 | WITHIN_NON_CODING_GENE | HCP5 |
| rs4627351 | 427 | 8.45E-05 | 16 | 2429894 | INTRONIC | N/A |
| rs3130837 | 428 | 8.47E-05 | 6 | 28948092 | DOWNSTREAM | AL662791.2 |
| rs2082455 | 429 | 8.47E-05 | 19 | 30007635 | INTERGENIC |  |
| rs9469007 | 430 | 8.56E-05 | 6 | 31409024 | WITHIN_NON_CODING_GENE | AL645933.3 |
| rs10498635 | 431 | 8.78E-05 | 14 | 93103309 | INTRONIC | N/A |
| rs1459084 | 432 | 8.81E-05 | 5 | 116403077 | INTERGENIC |  |
| rs7746199 | 433 | 8.87E-05 | 6 | 27261324 | INTRONIC | AL021808.2 |
| rs241446 | 434 | 8.88E-05 | 6 | 32796967 | INTRONIC | TAP2 |
| rs401775 | 435 | 9.04E-05 | 6 | 31931137 | INTRONIC | SKIV2L |
| rs7758512 | 436 | 9.07E-05 | 6 | 29970589 | WITHIN_NON_CODING_GENE | NCRNA00171 |
| rs241454 | 437 | 9.11E-05 | 6 | 32796144 | 3PRIME_UTR | TAP2 |
| rs10749643 | 438 | 9.14E-05 | 1 | 248264018 | 3PRIME_UTR | OR2L13 |
| rs12458804 | 439 | 9.20E-05 | 18 | 6490913 | INTERGENIC |  |
| rs3130955 | 440 | 9.31E-05 | 6 | 31054511 | DOWNSTREAM | AL662844.2 |
| rs3869068 | 441 | 9.41E-05 | 6 | 30004052 | WITHIN_NON_CODING_GENE | NCRNA00171 |
| rs7713177 | 442 | 9.48E-05 | 5 | 137588059 | DOWNSTREAM | GFRA3 |
| rs3132630 | 443 | 9.66E-05 | 6 | 30345118 | INTERGENIC |  |
| rs2517459 | 444 | 9.80E-05 | 6 | 30897022 | DOWNSTREAM | SFTA2 |
| rs13272882 | 445 | 9.87E-05 | 8 | 57109510 | INTRONIC | N/A |
| rs3132631 | 446 | 9.95E-05 | 6 | 30344645 | INTERGENIC |  |
| rs933688 | 447 | 0.0001 | 5 | 90762748 | INTERGENIC |  |
| rs6874279 | 448 | 0.0001 | 5 | 45393261 | INTRONIC | N/A |
| rs4803919 | 449 | 0.0001 | 19 | 46783256 | INTERGENIC |  |
| rs4346874 | 450 | 0.0001 | 6 | 31401374 | WITHIN_NON_CODING_GENE | N/A |
| rs9823570 | 451 | 0.0001 | 3 | 51833621 | INTERGENIC |  |
| rs28642901 | 452 | 0.0001 | 6 | 31381808 | INTRONIC | MICA |
| rs4711235 | 453 | 0.0001 | 6 | 30768636 | WITHIN_NON_CODING_GENE | C6orf214 |
| rs6692080 | 454 | 0.0001 | 1 | 227390162 | INTRONIC | N/A |
| rs11203501 | 455 | 0.0001 | 8 | 13489891 | INTERGENIC |  |
| rs2071475 | 456 | 0.0001 | 6 | 32782387 | INTRONIC | HLA-DOB |
| rs6708120 | 457 | 0.0001 | 2 | 242012759 | NON_SYNONYMOUS_CODING | SNED1 |
| rs10780513 | 458 | 0.0001 | 9 | 84012054 | INTERGENIC |  |
| rs3130696 | 459 | 0.0001 | 6 | 31243884 | WITHIN_NON_CODING_GENE | USP8P |
| rs7031894 | 460 | 0.0001 | 9 | 109204555 | WITHIN_NON_CODING_GENE | N/A |
| rs1894407 | 461 | 0.0001 | 6 | 32787036 | INTRONIC | HLA-DOB |
| rs241404 | 462 | 0.0001 | 6 | 32865997 | DOWNSTREAM | HLA-Z |
| rs3873380 | 463 | 0.0001 | 6 | 31262438 | INTERGENIC |  |
| rs7749305 | 464 | 0.0001 | 6 | 27446566 | INTERGENIC |  |
| rs10223568 | 465 | 0.0001 | 6 | 31390203 | WITHIN_NON_CODING_GENE | N/A |
| rs4607068 | 466 | 0.0001 | 3 | 139162994 | INTERGENIC |  |
| rs2780623 | 467 | 0.0001 | 1 | 230013938 | WITHIN_NON_CODING_GENE | AL354826.1 |
| rs2719237 | 468 | 0.0001 | 8 | 56926862 | DOWNSTREAM | LYN |
| rs1469357 | 469 | 0.0001 | 2 | 170638407 | DOWNSTREAM | KLHL23 |
| rs241449 | 470 | 0.0001 | 6 | 32796653 | 3PRIME_UTR | TAP2 |
| rs17803470 | 471 | 0.0001 | 2 | 95874133 | WITHIN_NON_CODING_GENE | AC092835.1 |
| rs7830138 | 472 | 0.0001 | 8 | 57084125 | INTRONIC | N/A |
| rs4259391 | 473 | 0.0001 | 8 | 99391285 | INTERGENIC |  |
| rs17040278 | 474 | 0.0001 | 12 | 108578230 | INTRONIC | N/A |
| rs2163558 | 475 | 0.0001 | 13 | 47606460 | INTERGENIC |  |
| rs6872928 | 476 | 0.0001 | 5 | 45047117 | INTERGENIC |  |
| rs7717790 | 477 | 0.0001 | 5 | 45221594 | INTERGENIC |  |
| rs2589170 | 478 | 0.0001 | 5 | 45547278 | INTRONIC | N/A |
| rs10055793 | 479 | 0.0001 | 5 | 49850054 | INTERGENIC |  |
| rs10471915 | 480 | 0.0001 | 5 | 49875076 | INTERGENIC |  |
| rs7710239 | 481 | 0.0001 | 5 | 50017369 | INTRONIC | N/A |
| rs241445 | 482 | 0.0001 | 6 | 32797072 | INTRONIC | TAP2 |
| rs5962226 | 483 | 0.0001 | X | 4605593 | INTERGENIC |  |
| rs2668012 | 484 | 0.0001 | 8 | 56926313 | DOWNSTREAM | LYN |
| rs9841622 | 485 | 0.0001 | 3 | 54120003 | INTERGENIC |  |
| rs4412752 | 486 | 0.0001 | 11 | 21667438 | INTERGENIC |  |
| rs4238910 | 487 | 0.0001 | 16 | 2475787 | 3PRIME_UTR | ABCA17P |
| rs281552 | 488 | 0.0001 | 18 | 65258604 | INTERGENIC |  |
| rs3823417 | 489 | 0.0001 | 6 | 31100869 | INTRONIC | PSORS1C2 |
| rs9674802 | 490 | 0.0001 | 17 | 49979895 | INTRONIC | N/A |
| rs12320036 | 491 | 0.0001 | 12 | 29593932 | INTRONIC | N/A |
| rs389883 | 492 | 0.0001 | 6 | 31947460 | 3PRIME_UTR | STK19 |
| rs1743511 | 493 | 0.0001 | 14 | 96145507 | 3PRIME_UTR | TCL6 |
| rs9263796 | 494 | 0.0001 | 6 | 31132883 | INTRONIC | POU5F1 |
| rs9468992 | 495 | 0.0001 | 6 | 31384578 | WITHIN_NON_CODING_GENE | MICA |
| rs28732164 | 496 | 0.0001 | 6 | 31861207 | INTRONIC | EHMT2 |
| rs9595529 | 497 | 0.0001 | 13 | 47338614 | INTERGENIC |  |
| rs2813969 | 498 | 0.0001 | 1 | 227404082 | INTRONIC | CDC42BPA |
| rs962876 | 499 | 0.0001 | 18 | 42127194 | INTERGENIC |  |
| rs241455 | 500 | 0.0001 | 6 | 32796019 | 3PRIME_UTR | TAP2 |
