# Supplementary material for: Common Genetic Variation and the Control of HIV-1 in Humans
Source: PLoS Genet. 2009 Dec 24;5(12):e1000791. doi: 10.1371/journal.pgen.1000791 (PMC2791220; doi:10.1371/journal.pgen.1000791)
Supplement: Table S10 — Top 500 SNPs in the progression analysis. (0.72 MB DOC) [file pgen.1000791.s014.doc]

**Table S10**: top 500 SNPs in the progression analysis

| SNP | rank | P value | chromosome | coordinate | type | gene |
| --- | --- | --- | --- | --- | --- | --- |
| rs9264942 | 1 | 6.40E-12 | 6 | 31274380 | UPSTREAM | HLA-C |
| rs2395029 | 2 | 1.20E-11 | 6 | 31431780 | WITHIN_NON_CODING_GENE | HCP5 |
| rs7758512 | 3 | 1.80E-08 | 6 | 29970589 | UPSTREAM | ZNRD1 |
| rs3869068 | 4 | 1.80E-08 | 6 | 30004052 | UPSTREAM | ZNRD1 |
| rs9261174 | 5 | 1.80E-08 | 6 | 29996855 | UPSTREAM | ZNRD1 |
| rs2074479 | 6 | 1.80E-08 | 6 | 30041009 | NON_SYNONYMOUS_CODING | RNF39 |
| rs2074480 | 7 | 1.80E-08 | 6 | 30040810 | INTRONIC | RNF39 |
| rs2301753 | 8 | 1.80E-08 | 6 | 30039240 | NON_SYNONYMOUS_CODING | RNF39 |
| rs9261129 | 9 | 1.80E-08 | 6 | 29979579 | UPSTREAM | ZNRD1 |
| rs3093662 | 10 | 5.00E-08 | 6 | 31544189 | INTRONIC | TNF |
| rs17291045 | 11 | 5.10E-08 | 4 | 161506897 | INTERGENIC |  |
| rs9368699 | 12 | 5.20E-08 | 6 | 31802541 | 5PRIME_UTR | SNORD52 |
| rs9497975 | 13 | 6.70E-08 | 6 | 148547802 | INTERGENIC |  |
| rs38152 | 14 | 6.80E-08 | 7 | 20047575 | WITHIN_NON_CODING_GENE | AC005062.2 |
| rs8013190 | 15 | 7.20E-08 | 14 | 58355048 | INTERGENIC |  |
| rs10484554 | 16 | 7.60E-08 | 6 | 31274555 | UPSTREAM | HLA-C |
| rs3815087 | 17 | 8.00E-08 | 6 | 31093587 | 5PRIME_UTR | CDSN |
| rs9263715 | 18 | 8.00E-08 | 6 | 31095801 | INTRONIC | CDSN |
| rs13207315 | 19 | 8.00E-08 | 6 | 31241127 | UPSTREAM | HLA-C |
| rs2249742 | 20 | 9.60E-08 | 6 | 31240721 | UPSTREAM | HLA-C |
| rs13191519 | 21 | 1.10E-07 | 6 | 31265752 | INTERGENIC |  |
| rs9367630 | 22 | 1.20E-07 | 6 | 55148103 | DOWNSTREAM | HCRTR2 |
| rs17324272 | 23 | 1.70E-07 | X | 132137156 | WITHIN_NON_CODING_GENE | N/A |
| rs2248462 | 24 | 2.50E-07 | 6 | 31446796 | DOWNSTREAM | HCP5 |
| rs16914280 | 25 | 2.70E-07 | 11 | 88321724 | INTRONIC | N/A |
| rs10501678 | 26 | 2.70E-07 | 11 | 88442102 | INTRONIC | N/A |
| rs2516509 | 27 | 3.20E-07 | 6 | 31449994 | DOWNSTREAM | HCP5 |
| rs2524123 | 28 | 3.60E-07 | 6 | 31265314 | INTERGENIC |  |
| rs1777672 | 29 | 3.80E-07 | 13 | 36177819 | INTRONIC | N/A |
| rs2516513 | 30 | 3.90E-07 | 6 | 31447588 | DOWNSTREAM | HCP5 |
| rs1051794 | 31 | 4.00E-07 | 6 | 31379109 | NON_SYNONYMOUS_CODING | MICA |
| rs12274302 | 32 | 4.30E-07 | 11 | 90586900 | INTERGENIC |  |
| rs13394720 | 33 | 4.50E-07 | 2 | 234502121 | INTERGENIC |  |
| rs2844795 | 34 | 5.10E-07 | 6 | 30073847 | INTRONIC | AL669914.2 |
| rs13210132 | 35 | 5.60E-07 | 6 | 31001143 | WITHIN_NON_CODING_GENE | AL669830.2 |
| rs9468932 | 36 | 9.30E-07 | 6 | 31264823 | INTERGENIC |  |
| rs12122100 | 37 | 9.80E-07 | 1 | 146508934 | WITHIN_NON_CODING_GENE | N/A |
| rs16948255 | 38 | 1.00E-06 | 16 | 74881820 | INTERGENIC |  |
| rs6751715 | 39 | 1.10E-06 | 2 | 56363377 | INTERGENIC |  |
| rs2894207 | 40 | 1.10E-06 | 6 | 31263751 | INTERGENIC |  |
| rs7772549 | 41 | 1.30E-06 | 6 | 31407643 | WITHIN_NON_CODING_GENE | AL645933.3 |
| rs3763313 | 42 | 1.50E-06 | 6 | 32376471 | INTRONIC | BTNL2 |
| rs4838508 | 43 | 1.50E-06 | 10 | 50561507 | INTERGENIC |  |
| rs7756521 | 44 | 1.60E-06 | 6 | 30848253 | UPSTREAM | DDR1 |
| rs3734905 | 45 | 1.70E-06 | 6 | 169958982 | INTRONIC | N/A |
| rs10517719 | 46 | 1.90E-06 | 4 | 161441065 | INTERGENIC |  |
| rs4151664 | 47 | 2.50E-06 | 6 | 31920873 | INTRONIC | RDBP |
| rs12198173 | 48 | 3.20E-06 | 6 | 32026808 | INTRONIC | TNXB |
| rs13199524 | 49 | 3.20E-06 | 6 | 32066765 | INTRONIC | TNXB |
| rs3830041 | 50 | 3.30E-06 | 6 | 32191339 | INTRONIC | NOTCH4 |
| rs4129945 | 51 | 3.40E-06 | 2 | 234493048 | UPSTREAM | UGT1A12P |
| rs11818629 | 52 | 3.40E-06 | 10 | 130137105 | INTERGENIC |  |
| rs17027625 | 53 | 3.60E-06 | 3 | 31445580 | INTERGENIC |  |
| rs3807035 | 54 | 3.60E-06 | 6 | 30044827 | UPSTREAM | RNF39 |
| rs1385351 | 55 | 3.90E-06 | 2 | 193333903 | INTERGENIC |  |
| rs3873334 | 56 | 4.00E-06 | 6 | 30896147 | DOWNSTREAM | VARS2 |
| rs3094212 | 57 | 4.10E-06 | 6 | 31085770 | INTRONIC | CDSN |
| rs13201769 | 58 | 4.90E-06 | 6 | 30756066 | WITHIN_NON_CODING_GENE | AL662797.4 |
| rs6665411 | 59 | 5.10E-06 | 1 | 146547095 | WITHIN_NON_CODING_GENE | AL596177.3 |
| rs3873332 | 60 | 5.20E-06 | 6 | 30895990 | DOWNSTREAM | VARS2 |
| rs9266845 | 61 | 5.50E-06 | 6 | 31384792 | WITHIN_NON_CODING_GENE | MICA |
| rs9295928 | 62 | 5.70E-06 | 6 | 30823630 | INTERGENIC |  |
| rs12697941 | 63 | 6.00E-06 | 6 | 30904714 | UPSTREAM | DPCR1 |
| rs2069084 | 64 | 6.30E-06 | 1 | 234984988 | INTERGENIC |  |
| rs1405262 | 65 | 6.40E-06 | 2 | 6134940 | WITHIN_NON_CODING_GENE | AC073479.1 |
| rs3807034 | 66 | 6.40E-06 | 6 | 30043992 | UPSTREAM | RNF39 |
| rs4713380 | 67 | 6.40E-06 | 6 | 30785273 | WITHIN_NON_CODING_GENE | C6orf214 |
| rs4713385 | 68 | 6.40E-06 | 6 | 30787593 | WITHIN_NON_CODING_GENE | C6orf214 |
| rs9258866 | 69 | 6.50E-06 | 6 | 29836524 | INTERGENIC |  |
| rs16936455 | 70 | 6.50E-06 | 8 | 70724754 | INTRONIC | N/A |
| rs12012519 | 71 | 6.50E-06 | X | 87190349 | INTERGENIC |  |
| rs11143609 | 72 | 6.60E-06 | 9 | 76158682 | INTERGENIC |  |
| rs7741100 | 73 | 6.90E-06 | 6 | 29768123 | WITHIN_NON_CODING_GENE | AL645939.5 |
| rs13198118 | 74 | 6.90E-06 | 6 | 30770732 | WITHIN_NON_CODING_GENE | C6orf214 |
| rs3873380 | 75 | 7.00E-06 | 6 | 31262438 | INTERGENIC |  |
| rs17868297 | 76 | 7.20E-06 | 2 | 234498651 | DOWNSTREAM | UGT1A12P |
| rs1120787 | 77 | 7.30E-06 | 18 | 54983554 | INTERGENIC |  |
| rs2304069 | 78 | 7.40E-06 | 5 | 149406733 | INTRONIC | AC011406.1 |
| rs2340462 | 79 | 7.40E-06 | 5 | 149415219 | INTRONIC | AC011406.2 |
| rs9391858 | 80 | 7.60E-06 | 6 | 32341398 | UPSTREAM | C6orf10 |
| rs10928302 | 81 | 7.80E-06 | 2 | 147372024 | INTERGENIC |  |
| rs6705842 | 82 | 7.80E-06 | 2 | 147393723 | INTERGENIC |  |
| rs1463132 | 83 | 8.00E-06 | 16 | 10183087 | INTRONIC | N/A |
| rs9266825 | 84 | 8.90E-06 | 6 | 31382882 | NON_SYNONYMOUS_CODING | MICA |
| rs2834812 | 85 | 9.10E-06 | 21 | 36533662 | WITHIN_NON_CODING_GENE | N/A |
| rs13437082 | 86 | 9.20E-06 | 6 | 31354560 | UPSTREAM | XXbac-BPG248L24.8 |
| rs10159302 | 87 | 9.40E-06 | 1 | 57805550 | INTRONIC | N/A |
| rs2637496 | 88 | 9.40E-06 | 5 | 124743267 | INTERGENIC |  |
| rs1230704 | 89 | 9.40E-06 | 18 | 55016718 | REGULATORY_REGION;UPSTREAM | ST8SIA3 |
| rs11971426 | 90 | 9.60E-06 | 7 | 20104908 | WITHIN_NON_CODING_GENE | N/A |
| rs9911992 | 91 | 9.70E-06 | 17 | 55917919 | INTRONIC | MRPS23 |
| rs6925061 | 92 | 1.00E-05 | 6 | 29992286 | WITHIN_NON_CODING_GENE | NCRNA00171 |
| rs9261285 | 93 | 1.00E-05 | 6 | 30036083 | 5PRIME_UTR | PPP1R11 |
| rs9295924 | 94 | 1.00E-05 | 6 | 30782361 | WITHIN_NON_CODING_GENE | C6orf214 |
| rs12114442 | 95 | 1.00E-05 | 8 | 15168644 | INTERGENIC |  |
| rs1653032 | 96 | 1.10E-05 | 8 | 13086762 | INTRONIC | N/A |
| rs11135875 | 97 | 1.10E-05 | 8 | 25477344 | INTERGENIC |  |
| rs7316626 | 98 | 1.10E-05 | 12 | 6691452 | SPLICE_SITE;INTRONIC | SCARNA11 |
| rs12099908 | 99 | 1.10E-05 | 12 | 6713636 | INTRONIC | CHD4 |
| rs17310951 | 100 | 1.10E-05 | X | 7770460 | INTERGENIC |  |
| rs12090529 | 101 | 1.20E-05 | 1 | 101707126 | DOWNSTREAM | S1PR1 |
| rs7597939 | 102 | 1.20E-05 | 2 | 39814690 | WITHIN_NON_CODING_GENE | AC007246.1 |
| rs9258525 | 103 | 1.20E-05 | 6 | 29806341 | INTERGENIC |  |
| rs6923832 | 104 | 1.20E-05 | 6 | 30062058 | INTERGENIC |  |
| rs2523619 | 105 | 1.20E-05 | 6 | 31318144 | DOWNSTREAM | HLA-B |
| rs9266830 | 106 | 1.20E-05 | 6 | -9 | N/A | N/A |
| rs10272685 | 107 | 1.20E-05 | 7 | 677650 | INTRONIC | N/A |
| rs11240053 | 108 | 1.30E-05 | 1 | 146923557 | INTERGENIC |  |
| rs16885750 | 109 | 1.30E-05 | 5 | 55874809 | INTRONIC | N/A |
| rs4711269 | 110 | 1.30E-05 | 6 | 31354819 | UPSTREAM | XXbac-BPG248L24.8 |
| rs6932542 | 111 | 1.30E-05 | 6 | 32380262 | UPSTREAM | BTNL2 |
| rs5744081 | 112 | 1.30E-05 | X | 12938471 | SYNONYMOUS_CODING | TLR8 |
| rs11263872 | 113 | 1.50E-05 | 1 | 35446830 | INTRONIC | ZMYM6 |
| rs12407202 | 114 | 1.50E-05 | 1 | 35462487 | INTRONIC | ZMYM6 |
| rs2540923 | 115 | 1.50E-05 | 2 | 37094945 | INTRONIC | AC007382.2 |
| rs2691111 | 116 | 1.50E-05 | 2 | 37102129 | INTRONIC | STRN |
| rs13430716 | 117 | 1.50E-05 | 2 | 37136517 | INTRONIC | STRN |
| rs13340131 | 118 | 1.60E-05 | 3 | 21794174 | WITHIN_NON_CODING_GENE | ZNF385D |
| rs6798138 | 119 | 1.60E-05 | 3 | 21816589 | WITHIN_NON_CODING_GENE | ZNF385D |
| rs17112462 | 120 | 1.60E-05 | 14 | 42455373 | INTERGENIC |  |
| rs9856731 | 121 | 1.70E-05 | 3 | 64782724 | WITHIN_NON_CODING_GENE | AC132007.1 |
| rs4142325 | 122 | 1.70E-05 | 3 | 64794743 | WITHIN_NON_CODING_GENE | AC132007.1 |
| rs7624656 | 123 | 1.70E-05 | 3 | 142585802 | INTRONIC | PCOLCE2 |
| rs6905949 | 124 | 1.70E-05 | 6 | 30140525 | DOWNSTREAM | TRIM15 |
| rs2302331 | 125 | 1.70E-05 | 7 | 6078376 | INTRONIC | EIF2AK1 |
| rs9655492 | 126 | 1.70E-05 | 7 | 6082211 | INTRONIC | EIF2AK1 |
| rs7811160 | 127 | 1.70E-05 | 7 | 85709526 | INTERGENIC |  |
| rs9378109 | 128 | 1.80E-05 | 6 | 30774474 | WITHIN_NON_CODING_GENE | C6orf214 |
| rs4711268 | 129 | 1.80E-05 | 6 | 31354504 | UPSTREAM | XXbac-BPG248L24.8 |
| rs10947207 | 130 | 1.80E-05 | 6 | 31361485 | INTERGENIC |  |
| rs9378200 | 131 | 1.80E-05 | 6 | 31572927 | INTERGENIC |  |
| rs10047299 | 132 | 1.80E-05 | 10 | 114701410 | INTERGENIC |  |
| rs3814570 | 133 | 1.80E-05 | 10 | 114708510 | UPSTREAM | TCF7L2 |
| rs721526 | 134 | 1.90E-05 | 18 | 25795214 | INTERGENIC |  |
| rs4800293 | 135 | 1.90E-05 | 18 | 25813277 | INTERGENIC |  |
| rs1472002 | 136 | 2.00E-05 | 1 | 188390606 | INTERGENIC |  |
| rs3796856 | 137 | 2.00E-05 | 4 | 15984033 | INTRONIC | N/A |
| rs10132770 | 138 | 2.00E-05 | 14 | 92243283 | DOWNSTREAM | TC2N |
| rs7158030 | 139 | 2.00E-05 | 14 | 92257063 | INTRONIC | N/A |
| rs1639123 | 140 | 2.10E-05 | 12 | 6714668 | INTRONIC | CHD4 |
| rs4128858 | 141 | 2.10E-05 | 12 | 118501598 | 3PRIME_UTR | AC131238.1 |
| rs9317355 | 142 | 2.20E-05 | 13 | 64151347 | INTERGENIC |  |
| rs12522255 | 143 | 2.30E-05 | 5 | 161761003 | INTERGENIC |  |
| rs8099301 | 144 | 2.30E-05 | 18 | 25838851 | INTERGENIC |  |
| rs153682 | 145 | 2.40E-05 | 5 | 52477252 | INTERGENIC |  |
| rs4713366 | 146 | 2.40E-05 | 6 | 30756361 | WITHIN_NON_CODING_GENE | AL662797.4 |
| rs9348876 | 147 | 2.40E-05 | 6 | 31575276 | DOWNSTREAM | AL662801.1 |
| rs17135086 | 148 | 2.40E-05 | 7 | 4805376 | 3PRIME_UTR | AC092610.3 |
| rs4294218 | 149 | 2.40E-05 | 8 | 137226607 | INTERGENIC |  |
| rs11115281 | 150 | 2.40E-05 | 12 | 77152232 | INTERGENIC |  |
| rs12369907 | 151 | 2.40E-05 | 12 | 77159952 | INTRONIC | ZDHHC17 |
| rs11115312 | 152 | 2.40E-05 | 12 | 77165609 | INTRONIC | ZDHHC17 |
| rs11115453 | 153 | 2.40E-05 | 12 | 77188745 | INTRONIC | N/A |
| rs11115513 | 154 | 2.40E-05 | 12 | 77205235 | INTRONIC | ZDHHC17 |
| rs12368947 | 155 | 2.40E-05 | 12 | 77252971 | INTRONIC | CSRP2 |
| rs10147227 | 156 | 2.40E-05 | 14 | 41875090 | INTERGENIC |  |
| rs16975363 | 157 | 2.40E-05 | 15 | 96121835 | INTERGENIC |  |
| rs9951414 | 158 | 2.40E-05 | 18 | 55018872 | UPSTREAM | ST8SIA3 |
| rs12888769 | 159 | 2.50E-05 | 14 | 39219051 | INTERGENIC |  |
| rs12893313 | 160 | 2.50E-05 | 14 | 39225303 | INTERGENIC |  |
| rs12893659 | 161 | 2.50E-05 | 14 | 39255581 | INTERGENIC |  |
| rs10084008 | 162 | 2.50E-05 | 18 | 4390081 | INTERGENIC |  |
| rs12734338 | 163 | 2.60E-05 | 1 | 202469723 | INTRONIC | PPP1R12B |
| rs4373258 | 164 | 2.60E-05 | 5 | 104745584 | INTERGENIC |  |
| rs720465 | 165 | 2.60E-05 | 6 | 31125777 | SPLICE_SITE;5PRIME_UTR | CCHCR1 |
| rs3130473 | 166 | 2.60E-05 | 6 | 31199208 | INTERGENIC |  |
| rs837466 | 167 | 2.60E-05 | 12 | 125010447 | INTRONIC | NCOR2 |
| rs16965248 | 168 | 2.60E-05 | 18 | 31940611 | INTERGENIC |  |
| rs6424271 | 169 | 2.70E-05 | 1 | 233176596 | INTRONIC | N/A |
| rs7728604 | 170 | 2.80E-05 | 5 | 168220102 | INTRONIC | AC011365.2 |
| rs12153855 | 171 | 2.80E-05 | 6 | 32074804 | INTRONIC | TNXB |
| rs17577039 | 172 | 2.90E-05 | 1 | 84204198 | WITHIN_NON_CODING_GENE | N/A |
| rs6683294 | 173 | 2.90E-05 | 1 | 84218373 | WITHIN_NON_CODING_GENE | N/A |
| rs250975 | 174 | 2.90E-05 | 2 | 234981061 | INTRONIC | SPP2 |
| rs3130424 | 175 | 2.90E-05 | 6 | 31218239 | INTERGENIC |  |
| rs1365080 | 176 | 2.90E-05 | 11 | 125603535 | INTERGENIC |  |
| rs723393 | 177 | 3.00E-05 | 4 | 23473797 | INTERGENIC |  |
| rs6829223 | 178 | 3.00E-05 | 4 | 23474539 | INTERGENIC |  |
| rs3823418 | 179 | 3.00E-05 | 6 | 31100942 | INTRONIC | PSORS1C1 |
| rs9366778 | 180 | 3.00E-05 | 6 | 31269173 | INTERGENIC |  |
| rs8192591 | 181 | 3.00E-05 | 6 | 32185796 | NON_SYNONYMOUS_CODING | NOTCH4 |
| rs4444435 | 182 | 3.00E-05 | 19 | -9 | N/A | N/A |
| rs982130 | 183 | 3.00E-05 | X | 104037621 | INTRONIC | N/A |
| rs29232 | 184 | 3.10E-05 | 6 | 29611431 | INTERGENIC |  |
| rs9261154 | 185 | 3.10E-05 | 6 | 29990937 | WITHIN_NON_CODING_GENE | NCRNA00171 |
| rs2844509 | 186 | 3.10E-05 | 6 | 31510924 | INTRONIC | ATP6V1G2 |
| rs10091480 | 187 | 3.10E-05 | 8 | 19129651 | INTRONIC | N/A |
| rs17000676 | 188 | 3.10E-05 | X | 133763174 | INTRONIC | N/A |
| rs1564041 | 189 | 3.20E-05 | 4 | 188215556 | INTERGENIC |  |
| rs6553055 | 190 | 3.20E-05 | 4 | 188219193 | INTERGENIC |  |
| rs4938943 | 191 | 3.20E-05 | 11 | 60174896 | INTRONIC | MS4A14 |
| rs12584939 | 192 | 3.20E-05 | 13 | 76510046 | INTERGENIC |  |
| rs2120577 | 193 | 3.30E-05 | 3 | 58551800 | 3PRIME_UTR | FAM107A |
| rs12525796 | 194 | 3.30E-05 | 6 | 23785745 | INTERGENIC |  |
| rs2844513 | 195 | 3.30E-05 | 6 | 31388214 | WITHIN_NON_CODING_GENE | MICA |
| rs11539542 | 196 | 3.30E-05 | 12 | 6709059 | SYNONYMOUS_CODING | CHD4 |
| rs2072038 | 197 | 3.40E-05 | 1 | 175105778 | INTRONIC | N/A |
| rs6432315 | 198 | 3.40E-05 | 2 | 12720311 | DOWNSTREAM | AC096559.2 |
| rs4669910 | 199 | 3.40E-05 | 2 | 13289018 | DOWNSTREAM | AC093912.1 |
| rs7907819 | 200 | 3.40E-05 | 10 | 73408096 | INTRONIC | CDH23 |
| rs1925757 | 201 | 3.40E-05 | 13 | 46186976 | INTRONIC | RP11-351K3.1 |
| rs11850521 | 202 | 3.40E-05 | 14 | 39279490 | INTERGENIC |  |
| rs2395471 | 203 | 3.50E-05 | 6 | 31240692 | UPSTREAM | HLA-C |
| rs872580 | 204 | 3.60E-05 | 2 | 95722909 | UPSTREAM | MAL |
| rs3112996 | 205 | 3.60E-05 | 2 | 95812372 | WITHIN_NON_CODING_GENE | MRPS5 |
| rs3105104 | 206 | 3.60E-05 | 2 | 95816365 | INTRONIC | ZNF514 |
| rs3112997 | 207 | 3.60E-05 | 2 | 95818151 | INTRONIC | ZNF514 |
| rs3112228 | 208 | 3.60E-05 | 2 | 95835614 | INTRONIC | ZNF2 |
| rs3112230 | 209 | 3.60E-05 | 2 | 95846158 | INTRONIC | ZNF2 |
| rs3105105 | 210 | 3.60E-05 | 2 | 95848018 | 3PRIME_UTR | ZNF2 |
| rs1875402 | 211 | 3.60E-05 | 2 | 95848354 | 3PRIME_UTR | ZNF2 |
| rs2320624 | 212 | 3.60E-05 | 2 | 95856611 | INTERGENIC |  |
| rs6725821 | 213 | 3.60E-05 | 2 | 95897767 | INTERGENIC |  |
| rs6748967 | 214 | 3.60E-05 | 2 | 95914095 | INTERGENIC |  |
| rs1966272 | 215 | 3.60E-05 | 2 | 95926154 | INTERGENIC |  |
| rs4854249 | 216 | 3.60E-05 | 2 | 95930630 | INTERGENIC |  |
| rs17013251 | 217 | 3.60E-05 | 2 | 126728027 | INTERGENIC |  |
| rs6586674 | 218 | 3.60E-05 | 8 | 17810464 | INTRONIC | N/A |
| rs12880269 | 219 | 3.60E-05 | 14 | 39210739 | INTERGENIC |  |
| rs2857106 | 220 | 3.70E-05 | 6 | 32787570 | INTRONIC | TAP2 |
| rs9792437 | 221 | 3.70E-05 | 9 | 123964901 | INTRONIC | AL137068.2 |
| rs10517724 | 222 | 3.80E-05 | 4 | 161685456 | INTERGENIC |  |
| rs166327 | 223 | 3.80E-05 | 6 | 30002881 | WITHIN_NON_CODING_GENE | NCRNA00171 |
| rs2109630 | 224 | 3.80E-05 | 18 | 25818973 | INTERGENIC |  |
| rs6039505 | 225 | 3.80E-05 | 20 | 9547288 | INTRONIC | AL353612.1 |
| rs4670135 | 226 | 3.90E-05 | 2 | 36270756 | INTERGENIC |  |
| rs9309403 | 227 | 3.90E-05 | 2 | 67911513 | UPSTREAM | AC007422.1 |
| rs6555421 | 228 | 3.90E-05 | 5 | 6990363 | INTERGENIC |  |
| rs12417691 | 229 | 3.90E-05 | 11 | 22015682 | INTERGENIC |  |
| rs16909097 | 230 | 3.90E-05 | 11 | 22078582 | INTERGENIC |  |
| rs7924927 | 231 | 3.90E-05 | 11 | 22086159 | INTERGENIC |  |
| rs2824816 | 232 | 3.90E-05 | 21 | 19791091 | INTRONIC | N/A |
| rs2270537 | 233 | 4.00E-05 | 1 | 32829281 | SYNONYMOUS_CODING | TSSK3 |
| rs3131003 | 234 | 4.00E-05 | 6 | 31093482 | 5PRIME_UTR | CDSN |
| rs864211 | 235 | 4.00E-05 | 7 | 16780932 | INTERGENIC |  |
| rs7012434 | 236 | 4.00E-05 | 8 | 67465588 | INTERGENIC |  |
| rs16990000 | 237 | 4.00E-05 | 20 | 10948813 | INTERGENIC |  |
| rs2523809 | 238 | 4.10E-05 | 6 | 29849619 | INTERGENIC |  |
| rs8022685 | 239 | 4.10E-05 | 14 | 39201336 | INTERGENIC |  |
| rs250977 | 240 | 4.20E-05 | 2 | 234978363 | INTRONIC | SPP2 |
| rs10402423 | 241 | 4.20E-05 | 19 | 1546180 | INTERGENIC |  |
| rs3112986 | 242 | 4.30E-05 | 2 | 95729338 | INTERGENIC |  |
| rs6713179 | 243 | 4.30E-05 | 2 | 193345479 | INTERGENIC |  |
| rs17775669 | 244 | 4.30E-05 | 3 | 78012759 | INTERGENIC |  |
| rs9261277 | 245 | 4.30E-05 | 6 | 30031091 | INTRONIC | ZNRD1 |
| rs2098874 | 246 | 4.30E-05 | 8 | 68607109 | INTRONIC | N/A |
| rs2154639 | 247 | 4.50E-05 | 8 | 101590470 | INTRONIC | SNX31 |
| rs12291186 | 248 | 4.50E-05 | 11 | 27696318 | INTRONIC | N/A |
| rs1364427 | 249 | 4.60E-05 | 7 | 134061955 | INTERGENIC |  |
| rs10509967 | 250 | 4.60E-05 | 10 | 114695932 | INTERGENIC |  |
| rs9261371 | 251 | 4.70E-05 | 6 | 30058988 | INTERGENIC |  |
| rs355220 | 252 | 4.70E-05 | 11 | 41064232 | INTERGENIC |  |
| rs17085438 | 253 | 4.70E-05 | 13 | 70394997 | INTRONIC | N/A |
| rs2911506 | 254 | 4.70E-05 | 13 | 70419939 | INTRONIC | N/A |
| rs9469003 | 255 | 4.80E-05 | 6 | 31407828 | WITHIN_NON_CODING_GENE | AL645933.3 |
| rs1028688 | 256 | 4.80E-05 | 20 | 41370400 | INTRONIC | N/A |
| rs4144871 | 257 | 4.80E-05 | X | 152837731 | INTRONIC | ATP2B3 |
| rs2111469 | 258 | 4.90E-05 | 2 | 56376308 | INTERGENIC |  |
| rs17148932 | 259 | 4.90E-05 | 11 | 55686109 | UPSTREAM | OR5W2 |
| rs4689148 | 260 | 5.00E-05 | 4 | 7637297 | INTRONIC | N/A |
| rs35148638 | 261 | 5.00E-05 | 5 | 86610989 | INTRONIC | N/A |
| rs9261394 | 262 | 5.00E-05 | 6 | 30064562 | INTERGENIC |  |
| rs2888830 | 263 | 5.00E-05 | 7 | 90915354 | INTERGENIC |  |
| rs11028502 | 264 | 5.00E-05 | 11 | 25208921 | INTERGENIC |  |
| rs805540 | 265 | 5.00E-05 | 20 | 37166358 | INTRONIC | AL049868.2 |
| rs2864932 | 266 | 5.00E-05 | 20 | 37186009 | INTRONIC | KIAA1219 |
| rs12694242 | 267 | 5.10E-05 | 2 | 212438861 | INTRONIC | N/A |
| rs7840440 | 268 | 5.10E-05 | 8 | 137379939 | INTERGENIC |  |
| rs17535055 | 269 | 5.10E-05 | 13 | 42512412 | INTRONIC | N/A |
| rs1303567 | 270 | 5.20E-05 | 20 | 37106157 | INTRONIC | KIAA1219 |
| rs8007195 | 271 | 5.30E-05 | 14 | 92276821 | INTRONIC | AL121839.1 |
| rs10432656 | 272 | 5.40E-05 | 2 | 33375032 | INTRONIC | N/A |
| rs11758336 | 273 | 5.40E-05 | 6 | 133939843 | INTERGENIC |  |
| rs1806507 | 274 | 5.40E-05 | 13 | 98038577 | INTRONIC | MBNL2 |
| rs7891633 | 275 | 5.50E-05 | X | 93349655 | INTERGENIC |  |
| rs9989786 | 276 | 5.60E-05 | 2 | 95967746 | INTRONIC | KCNIP3 |
| rs9257936 | 277 | 5.60E-05 | 6 | 29639776 | 3PRIME_UTR | MOG |
| rs2394734 | 278 | 5.60E-05 | 6 | 30046246 | UPSTREAM | RNF39 |
| rs7579823 | 279 | 5.70E-05 | 2 | 8160356 | WITHIN_NON_CODING_GENE | N/A |
| rs6752439 | 280 | 5.70E-05 | 2 | 8162049 | WITHIN_NON_CODING_GENE | N/A |
| rs11765705 | 281 | 5.70E-05 | 7 | 39106942 | INTRONIC | N/A |
| rs13227656 | 282 | 5.70E-05 | 7 | 128972202 | INTRONIC | N/A |
| rs9410964 | 283 | 5.70E-05 | 9 | 90405210 | INTRONIC | CTSL3 |
| rs1326818 | 284 | 5.70E-05 | 9 | 119639180 | INTRONIC | N/A |
| rs17512996 | 285 | 5.70E-05 | 14 | 102517132 | 3PRIME_UTR | DYNC1H1 |
| rs3848147 | 286 | 5.70E-05 | 15 | 64636754 | INTRONIC | N/A |
| rs10152936 | 287 | 5.70E-05 | 15 | 64656242 | DOWNSTREAM | KIAA0101 |
| rs17364090 | 288 | 5.80E-05 | 6 | 128832425 | INTRONIC | PTPRK |
| rs7783211 | 289 | 5.90E-05 | 7 | 82771395 | INTRONIC | PCLO |
| rs504896 | 290 | 5.90E-05 | 9 | 32596503 | INTERGENIC |  |
| rs2893745 | 291 | 5.90E-05 | 10 | 59327130 | INTERGENIC |  |
| rs10769108 | 292 | 5.90E-05 | 11 | 45071979 | INTERGENIC |  |
| rs12872080 | 293 | 5.90E-05 | 13 | 103576820 | INTERGENIC |  |
| rs6631214 | 294 | 5.90E-05 | X | 30975047 | INTRONIC | N/A |
| rs6631215 | 295 | 5.90E-05 | X | 30975509 | INTRONIC | N/A |
| rs216132 | 296 | 6.00E-05 | 5 | 149431259 | INTRONIC | HMGXB3 |
| rs313396 | 297 | 6.10E-05 | 11 | 103193087 | INTRONIC | N/A |
| rs6077567 | 298 | 6.10E-05 | 20 | 9569769 | INTRONIC | N/A |
| rs5935372 | 299 | 6.10E-05 | X | 12570439 | INTRONIC | N/A |
| rs6805138 | 300 | 6.20E-05 | 3 | 45071538 | INTRONIC | CLEC3B |
| rs10935162 | 301 | 6.20E-05 | 3 | 135018895 | INTERGENIC |  |
| rs17251434 | 302 | 6.30E-05 | 10 | 3932531 | INTERGENIC |  |
| rs12232754 | 303 | 6.30E-05 | 18 | -9 | N/A | N/A |
| rs17007727 | 304 | 6.40E-05 | 2 | 72303473 | INTERGENIC |  |
| rs13406776 | 305 | 6.40E-05 | 2 | 72305463 | INTERGENIC |  |
| rs10038824 | 306 | 6.40E-05 | 5 | 149369776 | DOWNSTREAM | SLC26A2 |
| rs35672116 | 307 | 6.40E-05 | 19 | 7991334 | INTRONIC | SNAPC2 |
| rs11542189 | 308 | 6.40E-05 | 19 | 8000001 | SYNONYMOUS_CODING | TIMM44 |
| rs1265159 | 309 | 6.50E-05 | 6 | 31140047 | REGULATORY_REGION;UPSTREAM | PSORS1C3 |
| rs3924923 | 310 | 6.50E-05 | 8 | 136162227 | INTERGENIC |  |
| rs6642287 | 311 | 6.50E-05 | X | 154780283 | INTRONIC | TMLHE |
| rs7539069 | 312 | 6.60E-05 | 1 | 187940867 | INTERGENIC |  |
| rs4671826 | 313 | 6.60E-05 | 2 | 67908949 | WITHIN_NON_CODING_GENE | AC007422.1 |
| rs8013239 | 314 | 6.60E-05 | 14 | 80803763 | INTERGENIC |  |
| rs7408605 | 315 | 6.60E-05 | 19 | 42337718 | DOWNSTREAM | AC020956.1 |
| rs902253 | 316 | 6.60E-05 | X | 39261018 | WITHIN_NON_CODING_GENE | AC091808.2 |
| rs3094165 | 317 | 6.70E-05 | 6 | 29833541 | INTERGENIC |  |
| rs540370 | 318 | 6.70E-05 | 11 | 123965631 | UPSTREAM | OR10D4P |
| rs10206235 | 319 | 6.80E-05 | 2 | 45389301 | DOWNSTREAM | AC009236.1 |
| rs6909253 | 320 | 6.80E-05 | 6 | 30055643 | INTERGENIC |  |
| rs6571086 | 321 | 6.80E-05 | 6 | 96606573 | INTRONIC | N/A |
| rs11955258 | 322 | 6.90E-05 | 5 | 37369958 | INTRONIC | NUP155 |
| rs17039750 | 323 | 7.00E-05 | 4 | 161477815 | INTERGENIC |  |
| rs2844680 | 324 | 7.00E-05 | 6 | 30946496 | UPSTREAM | MUC21 |
| rs12121525 | 325 | 7.10E-05 | 1 | 68223227 | INTRONIC | N/A |
| rs12207535 | 326 | 7.10E-05 | 6 | 111277745 | UPSTREAM | GTF3C6 |
| rs9719226 | 327 | 7.10E-05 | 7 | 670161 | INTRONIC | N/A |
| rs2109895 | 328 | 7.20E-05 | 9 | 123677827 | INTRONIC | N/A |
| rs9468925 | 329 | 7.30E-05 | 6 | 31258837 | UPSTREAM | AL671883.7 |
| rs10946255 | 330 | 7.30E-05 | 6 | 170356783 | INTERGENIC |  |
| rs1475386 | 331 | 7.30E-05 | 10 | 36134109 | INTERGENIC |  |
| rs10187471 | 332 | 7.40E-05 | 2 | 160289911 | INTRONIC | BAZ2B |
| rs13417010 | 333 | 7.40E-05 | 2 | 160295732 | INTRONIC | BAZ2B |
| rs2052455 | 334 | 7.50E-05 | 5 | 141025581 | INTRONIC | FCHSD1 |
| rs10985140 | 335 | 7.50E-05 | 9 | 123822837 | INTRONIC | N/A |
| rs10850845 | 336 | 7.50E-05 | 12 | 118017851 | INTRONIC | N/A |
| rs6561237 | 337 | 7.50E-05 | 13 | 46197823 | INTERGENIC |  |
| rs2162979 | 338 | 7.70E-05 | 5 | 152854349 | INTERGENIC |  |
| rs6547967 | 339 | 7.80E-05 | 2 | 29880877 | INTRONIC | N/A |
| rs10202670 | 340 | 7.80E-05 | 2 | 160310246 | NON_SYNONYMOUS_CODING | BAZ2B |
| rs9870669 | 341 | 7.80E-05 | 3 | 182625519 | INTRONIC | ATP11B |
| rs2868993 | 342 | 7.90E-05 | 2 | 56335979 | INTERGENIC |  |
| rs2209688 | 343 | 7.90E-05 | 10 | 36136605 | INTERGENIC |  |
| rs6438298 | 344 | 8.00E-05 | 3 | 115841684 | INTRONIC | N/A |
| rs7816685 | 345 | 8.00E-05 | 8 | 79658906 | INTRONIC | IL7 |
| rs34109485 | 346 | 8.00E-05 | 16 | 70450739 | INTRONIC | N/A |
| rs2804834 | 347 | 8.10E-05 | 10 | 36129521 | INTERGENIC |  |
| rs1123242 | 348 | 8.10E-05 | 13 | 46208065 | INTERGENIC |  |
| rs17056307 | 349 | 8.10E-05 | 18 | 72799796 | INTERGENIC |  |
| rs4671821 | 350 | 8.20E-05 | 2 | 67890323 | WITHIN_NON_CODING_GENE | N/A |
| rs9320354 | 351 | 8.20E-05 | 6 | 111336435 | INTRONIC | BXDC1 |
| rs10428324 | 352 | 8.30E-05 | 4 | 15983317 | INTRONIC | N/A |
| rs9313097 | 353 | 8.30E-05 | 5 | 5060591 | INTRONIC | AC010451.1 |
| rs12573587 | 354 | 8.30E-05 | 10 | 73413824 | INTRONIC | CDH23 |
| rs12417948 | 355 | 8.30E-05 | 11 | 124060875 | DOWNSTREAM | OR10D3P |
| rs12887714 | 356 | 8.30E-05 | 14 | 39294566 | INTERGENIC |  |
| rs10733651 | 357 | 8.40E-05 | 9 | 123858194 | INTRONIC | CEP110 |
| rs10081760 | 358 | 8.40E-05 | 9 | 123884306 | INTRONIC | CEP110 |
| rs1407912 | 359 | 8.40E-05 | 9 | 123906001 | INTRONIC | CEP110 |
| rs7023214 | 360 | 8.40E-05 | 9 | 123908345 | INTRONIC | CEP110 |
| rs2273988 | 361 | 8.40E-05 | 9 | 123928486 | INTRONIC | CEP110 |
| rs6906662 | 362 | 8.50E-05 | 6 | 32266506 | INTRONIC | C6orf10 |
| rs6480305 | 363 | 8.50E-05 | 10 | 69879709 | INTRONIC | N/A |
| rs1005913 | 364 | 8.50E-05 | 16 | 55504521 | INTERGENIC |  |
| rs2070334 | 365 | 8.50E-05 | 20 | 13433707 | INTRONIC | N/A |
| rs6525768 | 366 | 8.50E-05 | X | 145965406 | INTERGENIC |  |
| rs10049124 | 367 | 8.60E-05 | 3 | 34149458 | INTERGENIC |  |
| rs13067639 | 368 | 8.60E-05 | 3 | 135018070 | INTERGENIC |  |
| rs16905615 | 369 | 8.70E-05 | 8 | 137328144 | INTERGENIC |  |
| rs10849492 | 370 | 8.70E-05 | 12 | 6720795 | UPSTREAM | CHD4 |
| rs1005741 | 371 | 8.80E-05 | 2 | 59859867 | WITHIN_NON_CODING_GENE | N/A |
| rs16853737 | 372 | 8.90E-05 | 1 | 204425826 | INTRONIC | PIK3C2B |
| rs34484624 | 373 | 8.90E-05 | 5 | 133698224 | INTRONIC | CDKL3 |
| rs4758576 | 374 | 8.90E-05 | 11 | 2973880 | INTRONIC | NAP1L4 |
| rs7940694 | 375 | 8.90E-05 | 11 | 2979798 | INTRONIC | NAP1L4 |
| rs10499001 | 376 | 9.00E-05 | 6 | 96598066 | INTRONIC | N/A |
| rs565947 | 377 | 9.00E-05 | 11 | 123966117 | UPSTREAM | OR10D4P |
| rs7143791 | 378 | 9.00E-05 | 14 | 22238121 | DOWNSTREAM | AE000659.1 |
| rs9938941 | 379 | 9.00E-05 | 16 | 76972558 | INTERGENIC |  |
| rs6918160 | 380 | 9.10E-05 | 6 | 131154822 | INTRONIC | AL109938.1 |
| rs1056417 | 381 | 9.10E-05 | 11 | 125619578 | 3PRIME_UTR | PATE1 |
| rs7132776 | 382 | 9.10E-05 | 12 | 92652482 | INTERGENIC |  |
| rs16939961 | 383 | 9.10E-05 | 17 | 43401748 | DOWNSTREAM | AC003070.1 |
| rs6432163 | 384 | 9.20E-05 | 2 | 11159080 | INTERGENIC |  |
| rs17146951 | 385 | 9.20E-05 | 9 | 133299131 | INTRONIC | HMCN2 |
| rs4944707 | 386 | 9.20E-05 | 11 | 87154097 | INTERGENIC |  |
| rs7321462 | 387 | 9.20E-05 | 13 | 35448362 | INTERGENIC |  |
| rs17647647 | 388 | 9.20E-05 | 13 | 46749087 | INTRONIC | LCP1 |
| rs10141939 | 389 | 9.20E-05 | 14 | 39138477 | INTERGENIC |  |
| rs10789496 | 390 | 9.30E-05 | 1 | 47416441 | INTERGENIC |  |
| rs12086216 | 391 | 9.30E-05 | 1 | 104800294 | INTERGENIC |  |
| rs12527197 | 392 | 9.30E-05 | 6 | 154107836 | INTERGENIC |  |
| rs6980851 | 393 | 9.30E-05 | 8 | 68588027 | INTRONIC | N/A |
| rs7011473 | 394 | 9.30E-05 | 8 | 68598907 | INTRONIC | N/A |
| rs2239657 | 395 | 9.30E-05 | 9 | 123671520 | SYNONYMOUS_CODING | TRAF1 |
| rs10111409 | 396 | 9.40E-05 | 8 | 133118482 | INTRONIC | OC90 |
| rs3790529 | 397 | 9.50E-05 | 1 | 65280620 | 3PRIME_UTR | RAVER2 |
| rs9625159 | 398 | 9.50E-05 | 22 | 27552564 | WITHIN_NON_CODING_GENE | AL008638.3 |
| rs6973042 | 399 | 9.60E-05 | 7 | 52752010 | INTERGENIC |  |
| rs12660883 | 400 | 9.70E-05 | 6 | 30764420 | DOWNSTREAM | C6orf214 |
| rs16908768 | 401 | 9.70E-05 | 11 | 21948400 | INTERGENIC |  |
| rs1235694 | 402 | 9.70E-05 | 18 | 54998867 | INTERGENIC |  |
| rs11727760 | 403 | 9.80E-05 | 4 | 6352060 | INTRONIC | N/A |
| rs2999482 | 404 | 0.0001 | 1 | 204436640 | INTRONIC | PIK3C2B |
| rs1124777 | 405 | 0.0001 | 1 | 204438334 | SYNONYMOUS_CODING | PIK3C2B |
| rs1553921 | 406 | 0.0001 | 1 | 204438643 | SYNONYMOUS_CODING | PIK3C2B |
| rs2999479 | 407 | 0.0001 | 1 | 204446313 | INTRONIC | PIK3C2B |
| rs1980050 | 408 | 0.0001 | 1 | 204446688 | INTRONIC | PIK3C2B |
| rs7415479 | 409 | 0.0001 | 1 | 248621163 | DOWNSTREAM | OR2T2 |
| rs6735537 | 410 | 0.0001 | 2 | 88310990 | UPSTREAM | AC108479.4 |
| rs2695642 | 411 | 0.0001 | 3 | 22130392 | WITHIN_NON_CODING_GENE | N/A |
| rs26887 | 412 | 0.0001 | 5 | 53553740 | INTRONIC | N/A |
| rs244684 | 413 | 0.0001 | 5 | 133427110 | INTERGENIC |  |
| rs3904925 | 414 | 0.0001 | 6 | 111316885 | INTRONIC | BXDC1 |
| rs12670364 | 415 | 0.0001 | 7 | 8328880 | WITHIN_NON_CODING_GENE | N/A |
| rs2553651 | 416 | 0.0001 | 8 | 68615391 | INTRONIC | AC022861.7 |
| rs7019406 | 417 | 0.0001 | 9 | 130628024 | DOWNSTREAM | AK1 |
| rs12249419 | 418 | 0.0001 | 10 | 20272573 | INTRONIC | N/A |
| rs11064289 | 419 | 0.0001 | 12 | 6727749 | DOWNSTREAM | LPAR5 |
| rs11047814 | 420 | 0.0001 | 12 | 25227628 | INTRONIC | N/A |
| rs1941075 | 421 | 0.0001 | 18 | 12774848 | INTERGENIC |  |
| rs11585932 | 422 | 0.0001 | 1 | 65335095 | SYNONYMOUS_CODING | JAK1 |
| rs6717323 | 423 | 0.0001 | 2 | 82769739 | UPSTREAM | AC010105.1 |
| rs7584969 | 424 | 0.0001 | 2 | 82828982 | INTERGENIC |  |
| rs11886368 | 425 | 0.0001 | 2 | 82839226 | DOWNSTREAM | AC109638.1 |
| rs12623098 | 426 | 0.0001 | 2 | 139406360 | INTERGENIC |  |
| rs13393435 | 427 | 0.0001 | 2 | 180982120 | INTERGENIC |  |
| rs12487286 | 428 | 0.0001 | 3 | 56040203 | INTRONIC | N/A |
| rs6537286 | 429 | 0.0001 | 4 | 145310209 | INTERGENIC |  |
| rs4610282 | 430 | 0.0001 | 4 | 145320355 | INTERGENIC |  |
| rs4692675 | 431 | 0.0001 | 4 | 169808612 | INTRONIC | PALLD |
| rs9266409 | 432 | 0.0001 | 6 | 31336568 | UPSTREAM | AL671883.4 |
| rs9480703 | 433 | 0.0001 | 6 | 107190424 | WITHIN_NON_CODING_GENE | AL080314.2 |
| rs16905627 | 434 | 0.0001 | 9 | 2389032 | INTERGENIC |  |
| rs12269391 | 435 | 0.0001 | 10 | 117963418 | INTRONIC | N/A |
| rs3912124 | 436 | 0.0001 | 12 | 28861022 | INTERGENIC |  |
| rs11833187 | 437 | 0.0001 | 12 | 55944999 | 5PRIME_UTR | OR6C4 |
| rs12431698 | 438 | 0.0001 | 14 | 24526593 | INTRONIC | LRRC16B |
| rs7166529 | 439 | 0.0001 | 15 | 66924849 | INTERGENIC |  |
| rs7264662 | 440 | 0.0001 | 20 | 12511885 | INTERGENIC |  |
| rs17148074 | 441 | 0.0001 | X | 45663838 | INTERGENIC |  |
| rs584323 | 442 | 0.0001 | 1 | 146976471 | INTERGENIC |  |
| rs1547587 | 443 | 0.0001 | 2 | 12619109 | WITHIN_NON_CODING_GENE | N/A |
| rs11681312 | 444 | 0.0001 | 2 | 67904632 | WITHIN_NON_CODING_GENE | AC007422.1 |
| rs11675630 | 445 | 0.0001 | 2 | 67904808 | WITHIN_NON_CODING_GENE | AC007422.1 |
| rs11687980 | 446 | 0.0001 | 2 | 112991098 | INTRONIC | ZC3H8 |
| rs1379715 | 447 | 0.0001 | 3 | 56041187 | INTRONIC | N/A |
| rs4386675 | 448 | 0.0001 | 4 | 6351958 | INTRONIC | N/A |
| rs13117245 | 449 | 0.0001 | 4 | 151681337 | INTRONIC | N/A |
| rs446568 | 450 | 0.0001 | 5 | 14009188 | INTERGENIC |  |
| rs1619379 | 451 | 0.0001 | 6 | 29785235 | UPSTREAM | MICG |
| rs2256919 | 452 | 0.0001 | 6 | 29940750 | UPSTREAM | MICD |
| rs11967472 | 453 | 0.0001 | 6 | 31271285 | DOWNSTREAM | XXbac-BPG248L24.1 |
| rs2844529 | 454 | 0.0001 | 6 | 31353593 | UPSTREAM | XXbac-BPG248L24.8 |
| rs9501106 | 455 | 0.0001 | 6 | 31388109 | WITHIN_NON_CODING_GENE | MICA |
| rs3778504 | 456 | 0.0001 | 6 | 44189200 | INTRONIC | SLC29A1 |
| rs4723632 | 457 | 0.0001 | 7 | 1070748 | INTRONIC | AC073957.3 |
| rs852154 | 458 | 0.0001 | 7 | 6090727 | INTRONIC | EIF2AK1 |
| rs685415 | 459 | 0.0001 | 7 | 6092639 | INTRONIC | EIF2AK1 |
| rs581706 | 460 | 0.0001 | 7 | 6098301 | INTRONIC | EIF2AK1 |
| rs34161 | 461 | 0.0001 | 7 | 31727749 | INTRONIC | C7orf16 |
| rs12537425 | 462 | 0.0001 | 7 | 90888070 | INTERGENIC |  |
| rs10252923 | 463 | 0.0001 | 7 | 90905041 | INTERGENIC |  |
| rs2163085 | 464 | 0.0001 | 7 | 90907134 | INTERGENIC |  |
| rs10223928 | 465 | 0.0001 | 7 | 101045580 | INTRONIC | N/A |
| rs16938934 | 466 | 0.0001 | 8 | 75580419 | INTERGENIC |  |
| rs2067766 | 467 | 0.0001 | 8 | 136170673 | INTERGENIC |  |
| rs11166646 | 468 | 0.0001 | 8 | 137281470 | INTERGENIC |  |
| rs10509684 | 469 | 0.0001 | 10 | 97003001 | INTRONIC | PDLIM1 |
| rs313403 | 470 | 0.0001 | 11 | 103192532 | INTRONIC | N/A |
| rs17106909 | 471 | 0.0001 | 11 | 107286943 | NON_SYNONYMOUS_CODING | N/A |
| rs7302661 | 472 | 0.0001 | 12 | 68651459 | UPSTREAM | IL22 |
| rs8031461 | 473 | 0.0001 | 15 | 62876777 | INTERGENIC |  |
| rs5745909 | 474 | 0.0001 | 15 | 75641714 | INTRONIC | NEIL1 |
| rs2072077 | 475 | 0.0001 | 15 | 91449654 | SYNONYMOUS_CODING | MAN2A2 |
| rs4238618 | 476 | 0.0001 | 16 | 12946129 | INTERGENIC |  |
| rs7222425 | 477 | 0.0001 | 17 | 1474982 | DOWNSTREAM | SLC43A2 |
| rs9905214 | 478 | 0.0001 | 17 | 54501912 | INTRONIC | N/A |
| rs7815 | 479 | 0.0001 | 19 | 281360 | 3PRIME_UTR | PPAP2C |
| rs308033 | 480 | 0.0001 | 19 | 3099982 | INTRONIC | GNA11 |
| rs1110277 | 481 | 0.0001 | 20 | 4854682 | NON_SYNONYMOUS_CODING | SLC23A2 |
| rs7891547 | 482 | 0.0001 | X | 48420741 | 3PRIME_UTR | TBC1D25 |
| rs7878739 | 483 | 0.0001 | X | 48467186 | DOWNSTREAM | WDR13 |
| rs17148245 | 484 | 0.0001 | X | 48469670 | INTERGENIC |  |
| rs2275758 | 485 | 0.0001 | 1 | 53377125 | INTRONIC | ECHDC2 |
| rs3130517 | 486 | 0.0001 | 6 | 31190303 | INTERGENIC |  |
| rs3869129 | 487 | 0.0001 | 6 | 31410649 | WITHIN_NON_CODING_GENE | AL645933.3 |
| rs12663103 | 488 | 0.0001 | 6 | 32161324 | INTRONIC | GPSM3 |
| rs6910046 | 489 | 0.0001 | 6 | 111324495 | INTRONIC | AL357515.4 |
| rs9402428 | 490 | 0.0001 | 6 | 132889034 | UPSTREAM | TAAR6 |
| rs2717536 | 491 | 0.0001 | 8 | 79701876 | INTRONIC | N/A |
| rs10971038 | 492 | 0.0001 | 9 | 32592503 | INTERGENIC |  |
| rs7862059 | 493 | 0.0001 | 9 | 79282119 | INTRONIC | N/A |
| rs4447076 | 494 | 0.0001 | 10 | 88696381 | 3PRIME_UTR | MMRN2 |
| rs11024581 | 495 | 0.0001 | 11 | 2939705 | INTRONIC | SLC22A18 |
| rs2919034 | 496 | 0.0001 | 11 | 96607458 | INTERGENIC |  |
| rs1734117 | 497 | 0.0001 | 12 | 6721707 | INTERGENIC |  |
| rs11170062 | 498 | 0.0001 | 12 | 52650590 | INTRONIC | AC021066.1 |
| rs17111803 | 499 | 0.0001 | 12 | 73307641 | INTERGENIC |  |
| rs4964274 | 500 | 0.0001 | 12 | 109125988 | UPSTREAM | CORO1C |
