# Supplementary material for: Common Genetic Variation and the Control of HIV-1 in Humans
Source: PLoS Genet. 2009 Dec 24;5(12):e1000791. doi: 10.1371/journal.pgen.1000791 (PMC2791220; doi:10.1371/journal.pgen.1000791)
Supplement: Table S11 — Number of SNPs discarded during quality control procedures. (0.03 MB DOC) [file pgen.1000791.s015.doc]

**Table S11**: Number of SNPs that were discarded during quality control procedures (*see Text S1*) for all 4 genotyping chips that were used in the study

|  |  | **Illumina Beadchip** | | | |
| --- | --- | --- | --- | --- | --- |
|  |  | **550K v1** | **550K v3** | **1M v1** | **1M duo** |
| **Number of SNPs on chip** |  | 555352 | 561466 | 1072820 | 1199187 |
| ***Elimination criteria:*** | ***<99% call rate*** | *41211* | *20633* | *111050* | *77495* |
|  | ***insufficient MAF*** | *720* | *6791* | *53104* | *69807* |
| **Final number of SNPs for association study** |  | 513421 | 534042 | 908666 | 1051885 |
| **Total number of non-overlapping SNPs** |  | 1087002 | | | |
