# Supplementary material for: Common Genetic Variation and the Control of HIV-1 in Humans
Source: PLoS Genet. 2009 Dec 24;5(12):e1000791. doi: 10.1371/journal.pgen.1000791 (PMC2791220; doi:10.1371/journal.pgen.1000791)
Supplement: Text S1 — Supplementary text. (0.09 MB DOC) [file pgen.1000791.s016.doc]

**Common genetic variation and the control of HIV-1 in humans**

**Supplementary Text**

1. Cohorts
   1. Euro-CHAVI *2-4*
   2. MACS *5*
2. Determination of set point *6*
3. Quality control of the genotyping data *7-8*
4. Modified EIGENSTRAT method to control for stratification *9*
5. Simulation study for independent MHC associations *10-11*
6. Two-way interaction testing *11-12*
7. Gene Set Enrichment Analysis *13-14*
8. Distribution of functional variants *15*

**I. Cohorts**

**A. Euro-CHAVI**

The Center for HIV-AIDS Vaccine Immunology (CHAVI) is led by Barton Haynes (Duke University, Durham NC, USA). Its Host Genetics Core is led by David Goldstein (Duke University, Durham NC, USA). CHAVI is founded by the National Institute of Allergy and Infectious Diseases (USA), grant AI067854. The Euro-CHAVI Consortium is coordinated by A Telenti (University of Lausanne, Switzerland), with S Colombo (University of Lausanne, Switzerland) and B Ledergerber (University of Zurich, Switzerland).

Participating Cohorts/Studies (Principal Investigators / Number of subjects included in the study) are: Swiss HIV Cohort Study (SHCS), Switzerland (P Francioli / 925); Icona Foundation Study, Rome, Italy (A De Luca / 252); IrsiCaixa Foundation, Barcelona, Spain (J Martinez-Picado, with the help of J. Dalmau / 71); San Raffaele del Monte Tabor Foundation, Milan, Italy (A Castagna / 40); Danish Cohort, Denmark (N Obel / 35); Royal Perth Hospital, Perth, Australia (S Mallal / 32); Guy’s, King’s and St. Thomas’ Hospitals, United Kingdom (P Easterbrook / 19); Modena Cohort, Modena, Italy (A Cossarizza / 14); Hospital Clinic-IDIBAPS cohort, Barcelona, Spain (JM Gatell / 9).

**Swiss HIV Cohort Study (SHCS):**

FUNDING: The SHCS is funded by the Swiss National Science Foundation (3345–062041) and the SHCS research foundation.

MEMBERS: M. Battegay, E. Bernasconi, J. Böni, HC Bucher, Ph. Bürgisser, A. Calmy, S. Cattacin, M. Cavassini, R. Dubs, M. Egger, L. Elzi, M. Fischer, M. Flepp, A. Fontana, P. Francioli (President of the SHCS, Centre Hospitalier Universitaire Vaudois, CH-1011- Lausanne), H. Furrer (Chairman of the Clinical and Laboratory Committee), C. Fux, M. Gorgievski, H. Günthard (Chairman of the Scientific Board), H. Hirsch, B. Hirschel, I. Hösli, Ch. Kahlert, L. Kaiser, U. Karrer, C. Kind, Th. Klimkait, B. Ledergerber, G. Martinetti, B. Martinez, N. Müller, D. Nadal, F. Paccaud, G. Pantaleo, A. Rauch, S. Regenass, M. Rickenbach (Head of Data Center), C. Rudin (Chairman of the Mother & Child Substudy), P. Schmid, D. Schultze, J. Schüpbach, R. Speck, P. Taffé, A. Telenti, A. Trkola, P. Vernazza, R. Weber, S. Yerly.

**Icona Foundation Study, Italy:**

FUNDING: The Icona Foundation Study is supported by unrestricted educational grants of Abbott, Boehringer Ingelheim, Bristol-Myers Squibb, Gilead, GSK, Pfizer and Janssen-Cilag.

GOVERNING BODY: M Moroni (Chair), A Antinori, G Carosi, R Cauda, A d’Arminio Monforte, G Di Perri, M Galli, F Ghinelli, R Iardino, G Ippolito, A Lazzarin, F Mazzotta, R Panebianco, G Pastore, CF Perno.

STEERING COMMITTEE: A Ammassari, A Antinori, C Arici, C Balotta, P Bonfanti, MR Capobianchi, A Castagna, F Ceccherini-Silberstein, A Cozzi-Lepri, A d’Arminio Monforte, A De Luca, C Gervasoni, E Girardi, S Lo Caputo, F Maggiolo, R Murri, C Mussini, M Puoti, C Torti.

EPIDEMIOLOGY, BIOSTATISTICS AND DATA MANAGING GROUP: A Cozzi-Lepri, I Fanti, T Formenti, MCF Prosperi.

PARTICIPATING PHYSICIANS AND CENTERS IN ITALY: M Montroni, A Giacometti, A Costantini, A Riva (Ancona); U Tirelli, F Martellotta (Aviano-PN); G Pastore, N Ladisa, A Pierri (Bari); F Suter, F Maggiolo (Bergamo); M Borderi, G Verucchi, B Piergentili, (Bologna); G Carosi, G Cristini, C Torti, C Minardi, D Bertelli (Brescia); T Quirino, C Abeli (Busto Arsizio); PE Manconi, P Piano (Cagliari); J Vecchiet, K Falasca (Chieti); G Carnevale, S Lorenzotti (Cremona); F Ghinelli, L Sighinolfi (Ferrara); F Leoncini, F Mazzotta, M Pozzi, S Lo Caputo (Firenze); G Pagano, G Cassola, G Viscoli, A Alessandrini, R Piscopo, G Mazzarello (Genova); F Soscia, L Tacconi (Latina); A Orani, R Rossotti (Lecco); D Tommasi, P Congedo (Lecce); A Chiodera, P Castelli (Macerata); M Galli, A Lazzarin, G Rizzardini, I Schlacht, A d’Arminio Monforte, AL Ridolfo, A Foschi, A Castagna, S Salpietro, S Merli, S Melzi, MC Moioli, P Cicconi, T Formenti (Milano); R Esposito, C Mussini (Modena); A Gori, M Fiorino (Monza), N Abrescia, A Chirianni, CM Izzo, M De Marco, R Viglietti, E Manzillo (Napoli); C Ferrari, P Pizzaferri (Parma); F Baldelli, B Belfiori (Perugia); G Magnani, MA Ursitti (Reggio Emilia); M Arlotti, P Ortolani (Rimini); R Cauda, M Andreoni, A Antinori, G Antonucci, P Narciso, V Tozzi, V Vullo, A De Luca, M Zaccarelli, R Acinapura, P De Longis, MP Trotta, M Calbi, L Gallo, A Miccoli, F Carletti, (Roma); MS Mura, G Madeddu (Sassari); P Caramello, G Di Perri, GC Orofino, M Sciandra (Torino); E Raise, F Ebo (Venezia); G Pellizzer, D Buonfrate (Vicenza).

**IrsiCaixa Foundation** **Acute/Recent HIV-1 Infection cohort, Barcelona, Spain:**

FUNDING: The irsiCaixa cohort was supported in part by the Spanish Network for AIDS Research (RIS; ISCIII-RETIC RD06/006).

INVESTIGATORS: Judith Dalmau (1), Bonaventura Clotet (1,2), Eulàlia Grau (1), Rafaela Ayen (1), Rocío Bellido (1), José Moltó (2), Robert Muga (2), Guillem Sirera (2), Eugènia Negredo (2), José Ramón Santos (2), Jordi Puig (2), Cristina Miranda (2), Ingrid Martínez (2), Pere Domingo (3), Maria del Mar Gutiérrez (3), Maria Gràcia Mateo (3), David Dalmau (4).

(1) irsiCaixa Foundation, Badalona, Spain

(2) Hospital Germans Trias i Pujol, Badalona, Spain

(3) Hospital de la Santa Creu i Sant Pau, Barcelona, Spain

(4) Hospital Mútua de Terrassa, Terrassa, Spain

**San Raffaele Hospital, Milan, Italy:**

FUNDING: Fondazione Centro San Raffaele del Monte Tabor

COLLABORATORS: Adriano Lazzarin (1), Giuliana Fusetti (2), Salpietro Stefania (3)

(1) Vita-Salute San Raffaele University

(2) Clinic of Infectious Diseases, IRCCS San Raffaele Hospital, 20127 Milano, Italy

**Danish HIV Cohort Study, Denmark:**

FUNDING: The Danish HIV Cohort Study is funded mainly by Copenhagen University, University of Southern Denmark and the Danish AIDS Foundation.

COLLABORATORS: Jan Gerstoft (1), Gitte Kronborg (2), Court Pedersen (3)

(1) Copenhagen University Hospital, Rigshospitalet, Denmark

(2) Copenhagen University Hospital, Hvidovre, Denmark

(3) Odense University Hospital, Denmark

**Royal Perth Hospital, Perth, Australia:**

COLLABORATORS: Mina John, Larry Park, Susan Hermann

Centre for Clinical Immunology and Biomedical Statistics, Royal Perth Hospital and Murdoch University, Western Australia

**Guy’s, King’s and St. Thomas’ Hospitals, United Kingdom:**

COLLABORATORS: Kristin Kuldanek, Fatimah Karim

Kings College, London, United Kingdom

**Modena Cohort, Modena, Italy:**

FUNDING: Cristina Mussini funding comes from the Istituto Superiore di Sanità, VI Programma Nazionale di ricerca sull’AIDS 2006, Italy, Projects: “Eziopatogenesi, studi immunologici e virologici dell’HIV/AIDS” - Grant: 40G.43

COLLABORATORS: Milena Nasi (1), Vanni Borghi (2), Marcello Pinti (1), Cristina Mussini (2)

(1) Department of Biomedical Sciences, Section of General Pathology, University of Modena and Reggio Emilia, School of Medicine, Modena, Italy

(2) University Hospital, Modena

**Hospital Clinic-IDIBAPS Acute/Recent HIV-1 Infection cohort, Barcelona, Spain:**

FUNDING: The Hospital Clinic-IDIBAPS cohort is supported in part by the “Ministerio de Sanidad y Consumo, Instituto de Salud Carlos III, Madrid (Spain)”, Spanish Network for AIDS Research (RIS; ISCIII-RETIC RD06/006). Jose M. Miro receives a Research Grant from the “Institut d’Investigacions Biomèdiques August Pi i Sunyer (IDIBAPS)”.

INVESTIGATORS: J.M. Miro, M López-Dieguez, F. Agüero, T. Pumarola, M. Plana, M.C. Ligero, C. Gil, V. Sanchez, T. Gallart and J.M. Gatell.

**B. MACS**

The Multicenter AIDS Cohort Study (MACS) includes the following:

**Baltimore**: The Johns Hopkins University Bloomberg School of Public Health: Joseph B. Margolick (Principal Investigator), Barbara Crain, Adrian Dobs, Homayoon Farzadegan, Joel Gallant, Lisette Johnson, Shenghan Lai, Ned Sacktor, Ola Selnes, James Shepherd, Chloe Thio.

**Chicago**: Howard Brown Health Center, Feinberg School of Medicine, Northwestern University, and Cook County Bureau of Health Services: John P. Phair and Steven Wolinsky (Multiple Principal Investigators), Sheila Badri, Bruce Cohen, Craig Conover, Maurice O'Gorman, David Ostrow, Frank Palella.

**Los** **Angeles**: University of California, UCLA Schools of Public Health and Medicine: Roger Detels (Principal Investigator), Barbara R. Visscher (Co-Principal Investigator), Aaron Aronow, Robert Bolan, Elizabeth Breen, Anthony Butch, Thomas Coates, Rita Effros, John Fahey, Beth Jamieson, Otoniel Martínez-Maza, Eric N. Miller, John Oishi, Paul Satz, Harry Vinters, Dorothy Wiley, Mallory Witt, Otto Yang, Stephen Young, Zuo Feng Zhang.

**Pittsburgh**: University of Pittsburgh, Graduate School of Public Health: Charles R. Rinaldo (Principal Investigator), Lawrence Kingsley (Co-Principal Investigator), James T. Becker, Robert W. Evans, John Mellors, Sharon Riddler, Anthony Silvestre.

**Data Coordinating Center**: The Johns Hopkins University Bloomberg School of Public Health: Lisa P. Jacobson (Principal Investigator), Alvaro Muñoz (Co-Principal Investigator), Keri Althoff, Christopher Cox, Gypsyamber D'Souza, Stephen J. Gange, Elizabeth Golub, Janet Schollenberger, Eric C. Seaberg, Sol Su.

FUNDING: NIH: National Institute of Allergy and Infectious Diseases: Robin E. Huebner; National Cancer Institute: Geraldina Dominguez; National Heart, Lung and Blood Institute: Cheryl McDonald. UO1-AI-35042, 5-M01-RR-00052 (GCRC), UO1-AI-35043, UO1-AI-37984, UO1-AI-35039, UO1-AI-35040, UO1-AI-37613, and UO1-AI-35041.

Website located at <http://www.statepi.jhsph.edu/macs/macs.html>.

**II. Determination of set point**

First step: Identification of all eligible patients that had 3 or more stable plasma HIV RNA results in the absence of antiretroviral treatment, and met one of the following criteria: [A] a valid seroconversion date estimation proven by documents or biological markers; or [B], for seroprevalent patients, VL data over a period of at least 3 years, diverging by no more than 0.5 log.

Second step: elimination of outlier VL on the basis of clinical or biological arguments

1. (only for the Euro-CHAVI cohort) Visual inspection of all the viral load data in relation to other clinical documentation to determine if any VL should be excluded because it was coincident with vaccination, immune-modulating treatment, major trauma or laboratory problems.
2. (for both cohorts) Elimination of VL data that was coincident with and subsequent to disease progression (defined as a CD4 count below 350 cells) or initiation of cART.

Third step: elimination of VL not reflecting the steady-state: 3 types of outliers were identified, corresponding to the 3-phasic evolution of HIV-1 viremia:

1. (for seroconverters only) VL measured before the set point has been reached, part of the initial peak of viremia observed during primary HIV infection: they have to be measured during the first year after seroconversion and have a value >0.25 log10 higher than average of subsequent VL.
2. VL measured during the late phase of the disease: for patients with a significantly ascending VL slope, we kept only the first 3 results for calculation of the set point.
3. VL measured during the set point period, but conflicting with other available results; possibly linked to unreported interfering conditions, laboratory errors, transcription or data-management errors: defined as VL >0.5log higher or lower than average of all remaining points.

Fourth step: calculation of the set point as the average of all remaining VL results.

**III. Quality control of the genotyping data**

The following quality control steps were taken to make sure genotypes were correctly called.

1. Infinium BeadStudio Raw Data Analysis

The samples were brought into BeadStudio files using standard Illumina cluster files. Any sample that had very low intensity or a very low call rate using the Illumina cluster (<95%) was deleted. All SNPs that had a call frequency below 100% were then reclustered. Any sample that was below a 98% call rate after the reclustering was deleted. A total of 115 samples were deleted during this procedure. Next, a “1% rule” was applied where all SNPs that had a call frequency below 99% (i.e. more than 1% of samples were not called or were ambiguously called) were deleted. The number of SNPs that were discarded at this step for each genotyping chip is indicated in Table S11. We have seen (unpublished data) that SNPs with many samples not called (or potentially miscalled) can lead to false positives in statistical associations.

The reclustering step creates SNP calling errors, but we have identified a procedure to prevent the errant calls from being released in the final report. The SNP data is screened within BeadStudio by looking at two criteria. First, all SNPs with a cluster separation value below 0.3 are manually checked to ensure correct calls. Many of these SNPs can be manually fixed, but some have to be deleted. Next, any SNP (excluding X chromosome SNPs) with a Het Excess value between -1.0 to -0.1 and 0.1 to 1.0 are evaluated to determine if the raw and normalized data show a clean call. Any SNP cluster that doesn’t appear normal is deleted. This includes SNPs that appear to show a deletion (hemizygotes and homozygous deletion). The rationale behind is to avoid artifacts from either the chemistry or an interfering SNP during hybridization.

2. Minor allele frequency (MAF) check for data handling accuracy

This step performs a basic check of the data accuracy on the data flow pipeline from the output of the Illumina genotyping facility to the analytical process. We checked the MAF report from PLINK software (http://pngu.mgh.harvard.edu/~purcell/plink/) against the original locus report generated by genotyping facility. We checked that the two MAF reports match exactly.

3. Specification of gender

This step performs a check on the gender specification obtained from the phenotype database, using the observed genotypes of SNPs on chromosome X and Y. All individuals who were marked as “male” but with significant amount of heterozygous X genotypes (>=1%), or who were marked as “female” but with high frequency of homozygous X genotypes (>=80%) or Y genotype readings, were individually inspected against original data source. If no satisfactory correction could be obtained these individuals were excluded from further analyses. Five subjects were excluded at this step.

4. Cryptic relatedness

This step performs a check on the cryptic relatedness between study participants. We estimated the sharing of genetic information by estimating identity by descent (IBD) using the PLINK software. All pairs of DNA samples showing >=0.125 (estimated proportion of alleles IBD) were individually inspected and one sample in each pair was excluded from further analyses. Seventeen subjects were excluded at this step.

5. Genotype missing

This step performs a check whether the genotype missing is skewed towards high or low phenotype values and hence may give rise to spurious association P-values. We used PLINK software to perform this check on the top SNPs discussed in the paper.

6. Low MAF

We removed all SNPs with a MAF<0.0013. This criterion ensured that at least 6 individuals of the rare genotype are present in the dataset, to control for error in the estimation of asymptotic P-values (as alleles with MAF this low or lower have no chance of approaching significance). The number of SNPs that were discarded at this step for each genotyping chip is indicated in Table S11.

7. Hardy-Weinberg Equilibrium (HWE)

This step performs a check whether the observed genotype data deviate from HWE. We defined a deviation from HWE with a criterion of P-value less than 0.05. We performed this check using PLINK software on the top SNPs showing genome-wide significant association, and none of those SNPs deviated from HWE.

8. Recheck of the genotyping quality

The top SNPs showing genome-wide significant association were subject to a double check for their genotyping quality. This is an individual recheck on the raw and normalized data to be sure that it is called correctly as described in “Infinium BeadStudio Raw Data Analysis” process.

**IV. Modified EIGENSTRAT method to control for stratification**

This method derives the principal components (PC) of the correlations among gene variants and corrects for those correlations in the association tests. In principle, the principal components in the analyses should reflect population ancestry. We have noticed however that some of the leading axes appear to depend on other sources of correlation, such as sets of variants near one another that show extended association. We have documented the potential for inversions to create this effect and it may be created by other causes of extended LD as well. For this reason we inspected the SNP ‘loadings’ for each of the leading axes to determine if they depended on many or relatively few SNPs, as would be expected if the given axis reflected population ancestry or a more localized LD effect respectively.

We selected EIGENSTRAT axes for use as covariates to adjust for ancestry in subsequent linear regression analyses as follows (procedure repeated within each ancestry group).

1. To find EIGENSTRAT axes, we started with autosomal SNPs with MAF>0.01.

2. On inspection of SNP loadings for each PC axis, we found several of the top axes to be dominated by a small number of SNPs all mapping to the same region of the genome. For example, one axis was found to be dominated by SNPs mapping to a region of chr8p22-23.1 coinciding with a known inversion polymorphism.

3. To correct for these LD effects, and ensure that EIGENSTRAT axes reflected only effects that applied equally across the whole genome (as ancestry effects should), we re-applied principal components analysis to a reduced SNP set in which (i) certain known high LD regions were excluded (chr8:8000000..12000000, chr6:25000000..33500000, chr11:45000000..57000000, chr5:44000000..51500000); (ii) SNPs were thinned using the “--indep-pairwise” option in PLINK, such that all SNPs within a window size of 1500 (step size of 150) were required to have r2<0.2; (iii) Each SNP was regressed on the previous 5 SNPs, and the residual entered into the PC analysis.

4. Inspection of SNP loadings on all axes deemed significant by the Tracy-Widom method, using Q-Q plots against Normal expectation, revealed no axes dominated by single high-LD regions of the genome.

5. Tracy Widom tests nominated the first 12 resulting PC axes as significant (p<0.05). We then included the PC values emerging from the EIGENSTRAT analyses as covariates in all the regression models.

**V. Simulation study to investigate the properties of the statistical approach applied for searching independent SNP associations in the MHC region**

***Simulation Goal***

We have designed and applied a statistical approach to search independent associations in the MHC region. This approach starts with a forward model selection algorithm within a linear regression framework, and follows with a permutation procedure to control the potential inflated false positive rate due to selection over a large number of SNPs. By design, this approach is expected to be conservative, i.e., we asked if there is ANY independent association in the MHC region; we did not plan to find ALL independent associations.

We here present a simple simulation study designed to test whether our approach is indeed conservative under various scenarios, including different sample sizes and different linkage disequilibrium (LD) strengths between SNPs.

***Simulation Design***

1. A total of 295 SNPs were generated by simulation.

(A) 15 SNPs that show different strengths of association with HIV-1 load at set point (r^2^ ranging from 0.001% to 3.6%). By design, those 15 SNPs only depend on set point, and are independent of the previously identified genome-wide significant SNPs rs2395029 and rs9264942. Still, the 15 SNPs are somehow correlated because they all associate with set point.

(B) 30 SNPs that are in various LD with rs2395029 and rs9264942, with LD measures (r^2^) ranging from 0 to 0.95. By design, those 30 SNPs should be independent of viral set point. However, they will show some degree of (even significant) association with set point just because they are in LD with the very highly associated variants rs2395029 and rs9264942.

(C) 250 randomly generated SNPs, independent of set point and of rs2395029/ rs9264942.

2. Sample sizes: 3 different sample sizes were tested: 400, 800 and 2000 samples.

3. 1000 simulation runs were performed and results were averaged over all runs.

***Data Analyses and Results***

The same statistical approach that we used in the original dataset was applied to the simulated data (forward model selection algorithm within a linear regression framework). The statistical power was calculated from the 15 SNPs described above in (A). The power is defined as the percentage of simulation runs in which SNPs that are truly associated with the phenotype are selected at each association strength level. The false positive rate is estimated from SNPs in (B) and (C), and is defined as the percentage of the simulation runs in which null SNPs are selected.

**Observed statistical power:**

S1~S15 are the 15 SNPs that associate with set point independently of rs2395029 and rs9264942, as described in (A). The statistical power of detecting each one of them at various sample sizes is reported. At sample size = 400, the power for the SNP that explains the largest fraction of the phenotypic variability, i.e., 3.6% (r^2^=0.036), is 0.465. With sample size = 2000, the power is over 0.9 for a SNP that explains 0.9% (r^2^=0.009) of the variability in set point.

| Design SNP | S1 | S2 | S3 | S4 | S5 | S6 | S7 | S8 | S9 | S10 | S11 | S12 | S13 | S14 | S15 |
| --- | --- | --- | --- | --- | --- | --- | --- | --- | --- | --- | --- | --- | --- | --- | --- |
| r^2^ | 0 | 0.0001 | 0.002 | 0.005 | 0.006 | 0.0065 | 0.007 | 0.008 | 0.009 | 0.011 | 0.014 | 0.018 | 0.022 | 0.029 | 0.036 |
| Size=400 | 0 | 0 | 0 | 0.01 | 0 | 0.005 | 0.005 | 0.045 | 0.025 | 0.115 | 0.140 | 0.180 | 0.275 | 0.335 | 0.465 |
| Size=800 | 0 | 0.0100 | 0.010 | 0.025 | 0.020 | 0.050 | 0.120 | 0.175 | 0.230 | 0.405 | 0.480 | 0.665 | 0.795 | 0.860 | 0.920 |
| Size=2000 | 0 | 0.0000 | 0.030 | 0.119 | 0.208 | 0.366 | 0.505 | 0.871 | 0.931 | 0.990 | 0.990 | 1.000 | 1.000 | 1.000 | 1.000 |

**Observed average false positive rate over all null SNPs and all simulations:**

L_SNP1~L_SNP30 are the 30 SNPs that are described in (B); R_SNP1~R_SNP250 are the 250 SNPs described in (C). For both categories, the false positive rate at sample size = 2000 is much lower than the designed cut-off, i.e., the 5^th^ percentile of the test statistics in the permutation runs. This result suggests that our approach is conservative, as expected. It is observed, and expected, that our approach is more accurate in eliminating false SNPs in (B), i.e., SNPs in LD with rs2395029 and rs9264942, because it is known that a forward selection algorithm will only keep variables that have an additional, independent effect.

| Design SNP | L_SNP1~L_SNP30 | R_SNP1~R_SNP250 |
| --- | --- | --- |
| Size=400 | 0.01 | 0.075 |
| Size=800 | 0.0050 | 0.0250 |
| Size=2000 | 0.0000 | 0.0207 |

**VI. Two-way interaction testing**

***Rationale***

The essential problems for a systemic or genome-wide scan for interactions are that the greatly expanded search space implies a greater correction for multiple testing, that within this space the a priori probability of a true interaction per test is likely to be low, and that even if a true interaction is present the power to detect this is less than for a main effect of equivalent size. All these factors conspire to reduce power relative to a scan for single-locus effects. One solution to the first two of these problems is to adopt a directed search strategy by focusing on interactions involving a large main effect for one of the loci involved. While the existence of large interaction effects with little or no main effect in either locus remains a theoretical possibility, and while our knowledge of known interactions in the human genome is too fragmentary to exclude such cases, on a priori grounds it seems biologically more plausible that interactions are unlikely to achieve higher significance values than the stronger of the two interacting variants. This implies that in a genome-wide setting, the observed main effects must be substantially below the threshold for genome-wide significance to allow the subsequent identification of any interaction.

***Method***

Two-way interactions were investigated using multivariate linear models. The four variants, rs2395029 (HCP5/B*5701), rs9264942 (HLA-C) rs9261174 (ZNRD1/RNF39) and CCR5-Δ32, were fixed in the respective interaction models. All other SNPs were screened for significant interactions with each top-associated variant. For SNP rs2395029 (T/G), a dominant genetic model was used, since there are very limited patients with G/G genotypes, and there is no evidence that two copies of allele G are more protective than one in order to restrict HIV-1. For all other variants, an additive model was assumed. As an example of our method, the interaction between each SNP and rs2395029 can be interpreted as the difference of the additive patterns of setpoint variation on a specific SNP with different rs2395029 genotype (TT vs. TG+GG). The covariates adjusted in this multivariate linear model then include the main effect terms of the SNP screened and rs2395029, and other variables, such as age, gender and population stratification variables for controlling variations caused by those factors. And the P-value for the interaction term (SNP screened x rs2395029) was retained and reported. A conservative Bonferroni correction was used to correct for multiple testing. The tests were performed using both in-house C++ codes and the PLINK software.

**VII. Gene Set Enrichment Analysis (GSEA)**

A GSEA searches for groups of genes or pathways that are enriched in genetic variants with low association p-values and has the possibility to explore SNPs/genes other than those at the top significant level. A total of 425K SNPs, present on all genotyping chips, were mapped to 17K different genes and used in the analysis. Five gene sets were significant, with an FDR q-value < 0.25 (*Table S4*). Three of them have clear relevance to HIV-1 control: The “inflampathway” incorporates molecules involved in the coordination of the inflammatory response, notably cytokines; the “cskpathway” includes molecules involved in the negative regulation of T-cell receptor signaling; the “HSA04514 cell adhesion molecules” pathway contains molecules involved in T cell receptor signaling, leucocyte migration, complement cascade, as well as tight and adherens junctions. The other 2 identified pathways have no evidence of involvement in HIV-1 pathogenesis and could be false positives.

***GSEA with permutation test for continuous phenotype.*** Following the GSEA method for GWA (Wang et al., AJHG 2007) and the original GSEA method (Subramanian et al., PNAS 2005), we used the Illumina HumanHap550v3 gene annotation to map each SNP to a nearest gene, and also required the distance between the SNP and the mapped gene to be less than 500 kb. The maximum absolute value of the t-statistic (for linear regression) of all the SNPs mapped to a gene was used to represent the gene. For a total ***N*** genes, we ranked the absolute t-statistics by decreasing *t_(1)_ , t_(2)_ ,…, t_(i)_ ,…, t_(N)_* , and sorted genes according to the rank. For each given gene set ***S*** with set size ***N_s_***, we construct a weighted Kolmogorov-Smirnov like statistic, i.e. the enrichment score, ***ES(S)*** with parameter *p* = 1 (Formula 1).

$ES(S)=\max_{1\leq i\leq N} \left\{\sum_{\begin{matrix} g_{j}\in S \\ j\leq i \end{matrix}} \frac{\left|t_{(j)}\right|^{p}}{\sum_{g_{k}\in S} \left|t_{(k)}\right|^{p}}-\sum_{\begin{matrix} g_{j}\notin S \\ j\leq i \end{matrix}} \frac{1}{N-N_{S}}\right\}$ (1)

A permutation approach for continuous phenotype was implemented to estimate nominal p-values and correct set variation. In this work, we performed 1000 permutations on the data. Here the phenotype is viral load setpoint, a continuous variable; so the model used for detecting association is a linear regression model. We therefore swapped the label of phenotype, covariates and SNPs as following: keeping the association among phenotype, gender, age at seroconversion (clinical part); keeping the association among the eigen axes and SNPs (genetic part); only breaking the association between above two parts. For each combination between clinical part and genetic part, we performed linear regression to scan all the SNPs and obtain the corresponding *t*-statistics. The weighted Kolmogorov-Smirnov like statistics (enrichment score) for each permutation ***π*** and gene set ***S***, ***ES(π, S)***, were calculated as above. Given enrichment score ***ES(S_0_)***, nominal p value was the percentage of ***π*** with ***ES(π, S_0_)*** >= ***ES(S_0_)***.

We calculated the normalized enrichment score ***NES(S)*** by using ***ES(S)*** to substrate the mean of all ***ES(π, S)*** over ***π***, then divided by the standard deviation of all ***ES(π, S)*** over ***π*** (Formula 2). The normalized enrichment score ***NES(π***, ***S)*** was obtained in the same way.

$NES(S)=\frac{ES(S)-mean(ES(\pi,S))}{std(ES(\pi,S))}$ (2)

Multiple testing correction was assessed by the False Discovery Rate (FDR). For normalized enrichment score ***NES(S_0_)***, FDR q-value was calculated by the percentage of ***(π, S)*** with ***NES(π, S)*** >= ***NES(S_0_)***, dividing the percentage of ***S*** with ***NES(S)*** >= ***NES(S_0_)***. We retained as candidate gene sets those with a FDR q-value < 0.25.

**VIII. P-value distribution of functional variants**

A permutation procedure was developed to test whether functional genetic variants are more likely to have lower p-values. We firstly determined the functional category for each genetic variants we tested by using the Ensembl database version 50_36i. We derived the observed sum –Log_10_(P) for 12,535 functional polymorphisms that fell into one or more of these categories: stop-gained, stop-lost, fame-shift coding, non-synonymous coding, and essential splicing site genetic variants. To preserve the minor allele frequency (MAF) distributions of each of the 14 functional categories, we divided minor allele frequency into 10 groups and performed 10,000 permutations on functions within each MAF group. An empirical probability density distribution was then derived from these permutations, and the empirical probability for achieving the observed sum –Log_10_(P) by chance was calculated. We performed this analysis for functional genetic variants, as well as for 12,535 randomly selected intergenic (presumptively non-functional) genetic variants for better illustration of the results.

The distribution of the functional SNPs p-values was significantly skewed toward enrichment of low p-values *(Figure S4)*. To elucidate whether this signal was exclusively driven by functional variants in the MHC region, which contributes disproportionally to low p-values results, we then repeated the same analysis for that region only (318 functional variants) and for the rest of the genome (12217 functional variants). These analyses showed that functional variants in the MHC region, compared with the non-functional variants in the same region, were more likely to associate with differences in HIV-1 control (p=1E-03), while functional variants outside the MHC region were not (p=0.56).
